# The Periostin/Integrin-αv Axis Regulates the Size of Hematopoietic Stem Cell Pool in the Fetal Liver

**DOI:** 10.1016/j.stemcr.2020.06.022

**Published:** 2020-07-30

**Authors:** Atreyi Biswas, Irene M. Roy, Prathibha C. Babu, Javed Manesia, Sarah Schouteden, Vinod Vijayakurup, Ruby John Anto, Joerg Huelsken, Adam Lacy-Hulbert, Catherine M. Verfaillie, Satish Khurana

**Affiliations:** 1 School of Biology, Indian Institute of Science Education and Research Thiruvananthapuram, Thiruvananthapuram, Kerala 695551, India; 2 Inter-Departmental Stem Cell Institute, KU Leuven, 3000 Leuven, Belgium; 3 Rajiv Gandhi Centre for Biotechnology, Poojappura, Thiruvananthapuram, Kerala, India; 4 École Polytechnique Fédérale de Lausanne (EPFL), Lausanne, Switzerland; 5 Benaroya Research Institute at Virginia Mason, Seattle, WA 98101, USA

**Keywords:** hematopoietic stem cells, POSTN-ITGAV interaction, fetal liver, DNA damage responses, outside-in integrin signaling, fetal liver niche

## Abstract

We earlier showed that outside-in integrin signaling through POSTN-ITGAV interaction plays an important role in regulating adult hematopoietic stem cell (HSC) quiescence. Here, we show that *Itgav* deletion results in increased frequency of phenotypic HSCs in fetal liver (FL) due to faster proliferation. Systemic deletion of *Postn* led to increased proliferation of FL HSCs, albeit without any loss of stemness, unlike *Vav-Itgav*^*−/−*^ HSCs. Based on RNA sequencing analysis of FL and bone marrow HSCs, we predicted the involvement of DNA damage response pathways in this dichotomy. Indeed, proliferative HSCs from *Postn*-deficient FL tissues showed increased levels of DNA repair, resulting in lesser double-strand breaks. Thus POSTN, with its expression majorly localized in the vascular endothelium of FL tissue, acts as a regulator of stem cell pool size during development. Overall, we demonstrate that the duality of response to proliferation in HSCs is developmental stage dependent and can be correlated with DNA damage responses.

## Highlights

• Interruption of POSTN-ITGAV interaction leads to HSC expansion in fetal liver
• HSC from fetal liver in comparison with adult BM excel in their DNA damage responses
• POSTN is a potential component of the vascular niche for HSCs in the fetal liver

## Graphical Abstract

**Figure:**
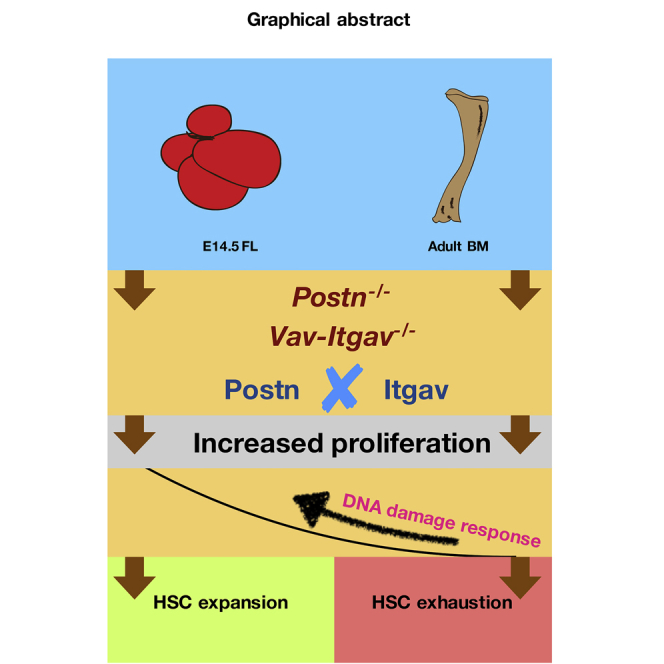

## Teaser

Khurana and colleagues demonstrate here a role of outside-in integrin signaling in regulating the size of hematopoietic stem cell (HSC) pool in mouse fetal liver. Using *Postn*^*−/−*^ and *Vav-Itgav*^*−/−*^ mice, they show that HSCs lacking POSTN-ITGAV interaction proliferate faster without any functional loss. The study proposes a possible role of increased levels of DNA damage responses in tolerating proliferation stress during fetal development.

## Introduction

Emergence and maturation of hematopoietic stem cells (HSCs) takes place during embryonic development where fetal liver (FL) plays a central role (Muller et al., 1994). Whereas bone marrow (BM)-resident adult HSCs are largely quiescent, fetal HSCs show robust proliferation to create the required stem cell pool (Bowie et al., 2006; Haas et al., 2018). It has been shown conclusively that HSCs differ majorly in their transcriptional profile across developmental stages (Manesia et al., 2015; McKinney-Freeman et al., 2012). The appearance of transient hematopoietic sites is a hallmark feature of the hematopoietic system during ontogeny and involves major changes in the HSC microenvironment across developmental stages (Boulais and Frenette, 2015). Little is known about fetal HSC niches, but recent evidence from Paul Frenette's group has shown the importance of cells associated with portal vessels in supporting HSCs in the FL tissue (Khan et al., 2016). This study follows closely another report from the same group wherein the arteriolar niche was shown to play host to the most primitive HSCs (Kunisaki et al., 2013). Significant genetic differences were observed in the cells that supported proliferative HSCs in FL tissue and the niche cells of the adult BM-derived HSCs (Khan et al., 2016). These differences notwithstanding, it has been sufficiently well established that association with their niches is crucial to the optimal functioning of fetal as well as adult HSCs (Gao et al., 2018).

Disruption of the interaction between HSCs and their microenvironment leads to deregulated proliferation, skewed differentiation, and decreased engraftment potential (Kiel and Morrison, 2008). Loss of integrin-α4, important for physical retention of HSCs in their niche, resulted in cell-cycle entry and spontaneous differentiation (Arroyo et al., 1999). Maintenance of quiescence in adult HSCs has been correlated with preservation of their long-term potential (Khurana, 2016). Repeated induction of proliferation in HSCs resulting from infection or chronic bleeding led to DNA damage and, ultimately, functional decline (Walter et al., 2015). However, as DNA damage responses (DDRs) are also linked to DNA replication process, Mohrin et al. (2010) showed that error-prone DDR pathways in quiescent HSCs lead to accumulation of DNA damage. By contrast, Beerman et al. (2014) showed that proliferative events in aged HSCs may lead to repair of damaged DNA. Thus, the precise link between proliferative events and stemness in the hematopoietic system remains unclear.

Integrins are some of the most crucial cell surface receptors that mediate retention of HSCs in the BM niche (Crane et al., 2017; Hoggatt et al., 2016; Khurana et al., 2013a). In addition, these receptors also mediate signaling cascades induced by growth factors or cytokines through outside-in signaling (Hynes, 2002). Whereas the importance of inside-out integrin signaling that regulates cell-cell or cell-extracellular matrix (ECM) adhesion is well worked out, the role of ligand-mediated outside-in signaling in hematopoiesis is not very well studied. Osteopontin, which elicits outside-in signaling cascades through integrin-αv (ITGAV; CD51) binding (Urtasun et al., 2012), acts as a negative regulator of HSC expansion (Nilsson et al., 2005; Stier et al., 2005). Integrin-αvβ3 (ITGAV-B3) also has a synergistic negative effect with the pro-inflammatory cytokine interferon-γ on HSC function (Umemoto et al., 2017). ITGB3 expression has been correlated with HSC quiescence (Umemoto et al., 2006) and ITGB3^hi^CD34^−^LSK cells were shown to be enriched for long-term repopulating HSCs (Umemoto et al., 2008). Periostin (POSTN) (Gillan et al., 2002) and Del1 (Mitroulis et al., 2017; Penta et al., 1999) have also been shown to mediate outside-in integrin signaling via binding to the ITGAV-B3 heterodimer. We recently reported that systemic deletion of *Postn* or *Vav-iCre* mediated conditional deletion of *Itgav* leads to the loss of quiescence in primitive HSCs, ultimately resulting in functional decline (Khurana et al., 2016).

Here, we report that the interruption of POSTN-ITGAV interaction causes increased proliferation of FL HSCs without any loss of stemness, resulting in their efficient expansion. This was unlike the effect of increased HSC proliferation on adult HSC function, indicating a developmental stage-specific response to proliferation rate. Our results linked better DDR in fetal HSCs with enhanced tolerance to proliferation stress. Overall, we show that the effect of proliferation on stemness is developmental stage dependent and is linked with DDR pathways.

## Results

### Expression of αv and β3 Integrin Chains in FL-Derived Primitive HSCs

**Figure 1:**
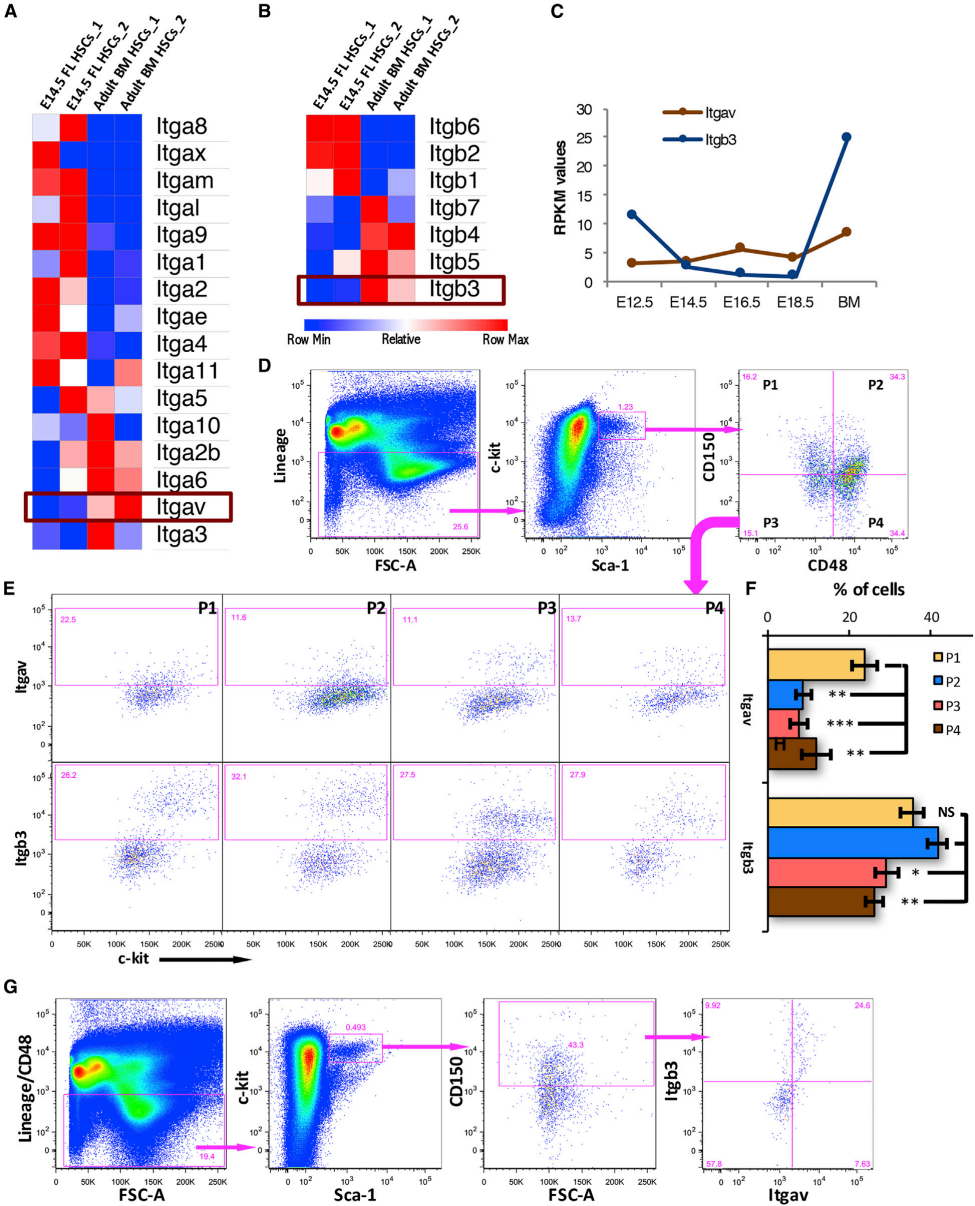
Expression of ITGAV and ITGB3 in FL HSCs Gene and protein expression for both α- and β-integrin chains that make a heterodimeric receptor for POSTN, analyzed using RNA-seq and flow cytometry, respectively. (A and B) Heatmaps showing differential expression of all known α-integrin (A) and β-integrin (B) chains analyzed by RNA-seq of primitive HSCs from E14.5 FL and adult BM. CD150^+^CD48^*−*^ LSK cells were sorted out from the two stages to perform paired end sequencing, reported in our earlier study. (C) *Itgav* and *Itgb3* expression in HSCs sorted from different developmental stages. Raw reads were subjected to quality control and high quality reads were aligned to mouse reference genome mm9. Reads per kilobase per million (RPKM) values obtained for *Itgav* and *Itgb3* expression across developmental stages were plotted. (D) E14.5 FL cells were analyzed for the cell surface expression of ITGAV and ITGB3 on various HSC sub-populations. Lin^*−*^c-kit^+^Sca-1^+^ (LSK) population was further divided into four sub-populations on the basis of expression of SLAM markers CD48 and CD150. (E) All four sub-populations of the LSK cells were examined for the expression of ITGAV (upper panel) and ITGB3 (lower panel), separately. (F) Proportion of different HSC sub-populations that expressed ITGAV and ITGB3. n = 4, one-way ANOVA followed by Tukey-Kramer post hoc test: ^∗^p < 0.05, ^∗∗^p ≤ 0.01, ^∗∗∗^p ≤ 0.005, NS, not significant. (G) Co-expression analysis of ITGAV and ITGB3 on the primitive FL HSCs. The E14.5 FL-derived HSCs were identified as lin^−^CD48^−^c-kit^+^Sca-1^+^CD150^+^ cells and were examined for the expression of ITGAV and ITGB3 based on flow cytometry. See also Figure S1.

We first examined the expression of ITGAV and its binding partner ITGB3 in embryonic day 14.5 (E14.5) FL HSCs (lin^−^c-kit^+^Sca-1^+^CD48^−^CD150^+^ cells; Figure 1). We analyzed our previously published RNA sequencing (RNA-seq) data (Manesia et al., 2015) to compare the expression of all known α-integrin (Figure 1A) and β-integrin (Figure 1B) chains. The heatmap analysis showed lower expression of both *Itgav* and *Itgb3* in E14.5 FL-derived HSCs. In fact, the expression of *Itgav* and *Itgb3* was observed to be low in HSCs from all embryonic stages (Figure 1C, S1A, and S1B), consistent with our earlier published results that established POSTN-ITGAV interaction as a negative regulator of BM-HSC proliferation. Importantly, we detected high levels of expression of integrins, such as *Itga4*, *Itga5*, *Itga6*, *Itgb1*, which are known to play an important role in HSC function, in FL-derived HSCs (Figures S1A and S1B). To confirm the relative abundance of *Itgav* and *Itgb3* in BM versus E14.5 FL HSCs, we performed qRT-PCR using freshly sorted cells (Figure S1C). We confirmed that the transcript levels of both *Itgav* and *Itgb3* were significantly higher in the BM versus FL HSCs.

We next performed flow cytometry analysis of FL-derived mononuclear cells (MNCs) to examine the expression of ITGAV and ITGB3 in different sub-populations within the stem cell compartment (Figures 1D–1F, S1D, and S1E). On the basis of expression of SLAM markers CD150 and CD48, the lin^−^c-kit^+^Sca-1^+^ (LSK) population (isotype antibody controls are shown in Figure S1D) was subdivided into four sub-populations; CD150^+^CD48^*−*^ (P1), CD150^+^CD48^+^ (P2), CD150^−^CD48^*−*^ (P3), and CD150^−^CD48^+^ (P4) (Figure 1D). Subsequently, the expression of ITGAV (upper panel) as well as ITGB3 (lower panel) in each of these populations was assessed (Figure 1E; details of gating strategies with isotype antibody and FMO controls in Figures S1D and S1E). Results showed that 23.80% ± 3.21% of the most primitive HSCs (P1) from E14.5 FL MNCs expressed Itgav (Figure 1F). The expression of ITGAV in all other sub-populations was lower than the primitive HSC population (p = 0.002; one-way ANOVA followed by Tukey-Kramer test). As Itgav has been shown to partner with ITGB3 to form a heterodimeric integrin receptor in a variety of cells (Savill et al., 1990), including a number of cancers (Eliceiri and Cheresh, 2001), we investigated the expression of ITGB3 in the stem cell sub-populations. The CD150^+^CD48^*−*^ (P1; 35.3% ± 2.72%) and CD150^+^CD48^+^ sub-populations (P2; 41.57% ± 6.17%) expressed significantly higher levels of ITGB3 than CD150^−^CD48^*−*^ (P3; 29.06% ± 2.90%) and CD150^−^CD48^+^ (P4; 26.13% ± 1.97%) sub-populations (Figure 1F, p = 0.008; one-way ANOVA followed by Tukey-Kramer test). We then examined the level of co-expression of ITGAV and ITGB3 on the most primitive HSC population (CD150^+^CD48^*−*^ LSK population). We observed that 76.25% ± 6.8% of the primitive HSCs from E14.5 FL that expressed ITGAV also expressed ITGB3 (Figure 1G; details of gating strategies with isotype antibody controls in Figures S1D–S1F). Therefore, notwithstanding lower transcript levels of *Itgav* and *Itgb3* in primitive HSCs all across FL stages when compared with the adult BM-derived cells, we observed a significant level of cell surface expression of both ITGAV and ITGB3.

### Loss of ITGAV in the Hematopoietic System Leads to Increased Frequency of Phenotypic FL Hematopoietic Stem and Progenitor Cells

To understand the role of POSTN-ITGAV interaction, we conditionally deleted *Itgav* in the hematopoietic system by crossing *Itgav*^*fl/fl*^ (Lacy-Hulbert et al., 2007) with *Vav-*iCre mice (de Boer et al., 2003) (Figure S2A). Tissue from the hindlimb bud of each E14.5 fetus was kept for genotyping to detect the wild-type (WT) or floxed *Itgav* allele (Figure S2B) and the *Vav-*iCre (Figure S2C) transgene. Genotyping confirmed normal monohybrid Mendelian genotypic ratios between *Vav-Itgav*^*+/+*^ (WT; 26.67%), *Vav-Itgav*^*+/−*^ (heterozygous [HT]; 48.33%), and *Vav-Itgav*^*−/−*^ (conditional knockout [cKO]; 25%) fetuses (χ^2^_df=2_ = 0.049, p = 0.98) (Figure S2D) that appeared healthy with no gross change in morphology (Figure S2E). We did observe modest but significant increase in total FL cellularity upon biallelic deletion of *Itgav* (Figure S2F). We then confirmed the lack of *Itgav* expression in the sorted LSK population by qRT-PCR (Figures S3A and S3B). In addition, the absence of cell surface expression of ITGAV in the primitive HSC population (SLAM LSK cells) was confirmed by flow cytometry (Figure S3C).

**Figure 2:**
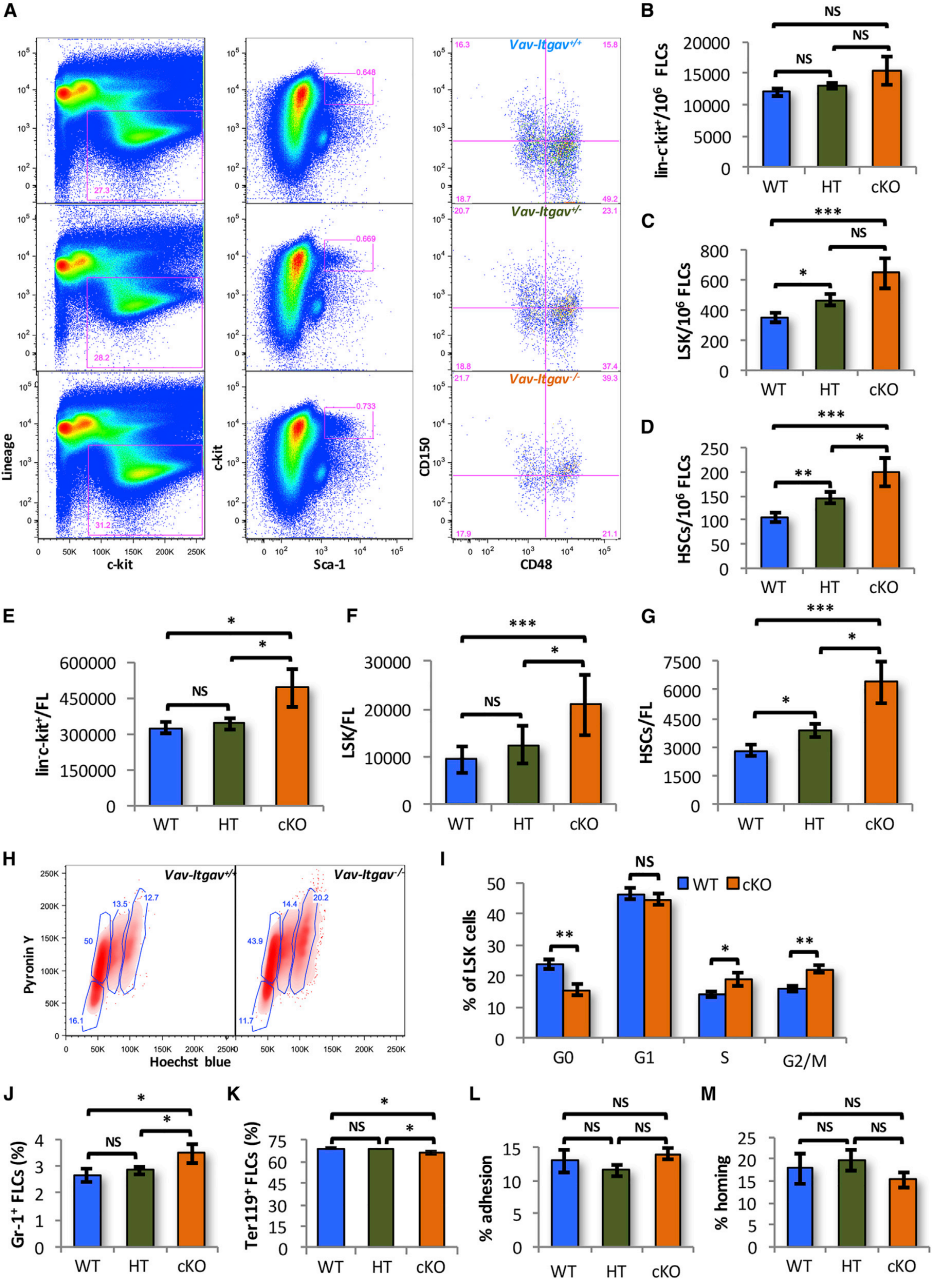
Increased Frequency of Phenotypic HSCs in E14.5 *Vav-Itgav*^*−/−*^ FL Tissues (A) *Vav-*iCre and *Itgav*^*fl/fl*^ mice were crossed to conditionally delete *Itgav* in hematopoietic system. The embryos were harvested at E14.5 and genotyped to identify *Vav-Itgav*^*+/+*^ (WT), *Vav-Itgav*^*+/−*^ (HT), *Vav-Itgav*^*−/−*^ (cKO) embryos. FL tissue was used for analysis of HSC frequency in by flow cytometry using specific antibodies. (B–D) Quantification of frequency of various hematopoietic stem and progenitor cell populations in the FL tissue from WT, HT, and cKO embryos at E14.5; (B) lin^*−*^c-kit^+^ cells, (C) LSK cells, (D) primitive HSCs. (E–G) Comparison of the total number of hematopoietic stem and progenitor cells per FL from WT, HT, and cKO embryos at E14.5; (E) lin^*−*^c-kit^+^ cells, (F) LSK cells, (G) primitive HSCs. (H) Cell-cycle analysis of the LSK cells harvested from E14.5 FL tissues by Hoechst 33342/pyronin Y staining (n = 6). (I) Comparison of the proportion of FL-derived LSK cells in various stages of cell cycle. (J and K) Flow cytometry-based analysis performed to compare the frequency of lineage-committed cells in E14.5 FL tissue from WT, HT, and cKO embryos. (J) Granulocytes identified as Gr-1^+^ cells, (K) erythrocytes identified as Ter119^+^ cells. (L) Adhesion potential of E14.5 *Vav;Itgav*^*+/+*^ (WT), *Vav;Itgav*^*+/-*^ (HT), and *Vav;Itgav*^*−/−*^ (cKO) FL-derived LSK cells was compared through *in vitro* adhesion assays. Freshly isolated LSK cells were allowed to adhere on ST2 cell feeder. The percentage of cells that adhered to the feeder after 3 h was plotted for each condition (n = 4). (M) Whole FL cells from *Vav;Itgav*^*+/+*^ (WT), *Vav;Itgav*^*+/−*^ (HT), and *Vav;Itgav*^*−/−*^ (cKO) mice, were infused in lethally irradiated animals. The percentage of transplanted colony-forming cells that homed into the BM within 16 h was plotted (n = 2, N = 8). An unpaired two-tailed Student's t test was performed. n = 2–4, N = 8–14, t test: ^∗^p < 0.05, ^∗∗^p ≤ 0.01, ^∗∗∗^p ≤ 0.005. See also Figures S2 and S3.

Next, we compared the frequency of hematopoietic stem and progenitor cells (HSPCs) (lin^−^c-kit^+^ cells), LSK cells, and primitive HSCs in E14.5 FL tissues (Figures 2A–2D). Although we did not observe any significant change in the frequency of lin^−^c-kit^+^ cells (Figure 2B), we did observe a significant increase in the frequency of LSK cells (Figure 2C) as well as primitive HSCs (Figure 2D) following *Itgav* deletion. Interestingly, even monoallelic deletion of *Itgav* (HT) resulted in an increase in the frequency of LSK and primitive HSCs (Figures 2C and 2D). As we observed increased total FL cellularity upon *Itgav* deletion, we compared the total numbers of HSPCs, LSK cells, and HSCs (SLAM LSK cells) per FL (Figures 2E–2G). We observed an increase in the number of LSK cells and primitive HSC population in *Vav-Itgav*^*−/−*^ FL tissues. Following monoallelic deletion of *Itgav*, however, we could see an increase in the primitive HSC population only (Figure 2G). We then addressed if this increase in the frequency of HSPC populations was accompanied by an altered proliferation status. Cell-cycle analysis of the FL cells using Hoechst and Pyronin Y staining (Figures 2H and 2I) showed only 23.63% ± 1.57% of the LSK cells to be quiescent. In *Vav-Itgav*^*−/−*^ fetuses, we observed a significant decrease in the proportion (15.58% ± 1.74%) of quiescent LSK cells (Figure 2I). This was accompanied by an increase in the proportion of LSK cells in S phase from 13.96% ± 1.03% in WT to 19.06% ± 2.26% in *Vav-Itgav*^*−/−*^ fetuses. Similar increase in the percentage of FL LSK cells in G2/M phase of cell cycle was also observed (16.02% ± 1.06% in WT to 22.23% ± 1.42% in cKO). We did not detect any changes in the proportion of these cells in the G1 phase of cell cycle (Figure 2I). These results indicated that the loss of *Itgav* expression in the hematopoietic cells led to increased proliferation rates.

We then performed flow cytometry-based analysis of FL-derived MNCs to detect any differences in the lineage-committed cell populations upon *Itgav* deletion (Figures 2J, 2K, and S3D–S3G). Results demonstrated a modest but significant increase in the proportion of Gr-1^+^ cells in *Vav-Itgav*^*−/−*^ FL tissues (Figures 2J and S3D, upper panel). We did not observe any change in the macrophage population identified by F4/80 expression (Figure S3E). Evaluation of B and T cell lineages identified as CD19^+^ (Figure S3F) and CD3e^+^ cells (Figure S3G) respectively, showed no change upon *Itgav* deletion. We also examined whether the proportion of erythroid population that makes the bulk of FL cellularity is changed upon *Itgav* deletion (Figures 2K and S3D, lower panel). We observed that biallelic deletion of *Itgav* led to a modest but significant decrease in the erythroid cell population from 69.03% ± 0.58% to 65.08% ± 2.21%.

As integrins are known for their role as cell surface adhesion receptors, we examined if the lack of ITGAV had any effect on stem cell attachment that could affect their homing and engraftment. We performed *in vitro* adhesion assays using ST2 cell feeders and PKH26-labeled LSK cells. We observed no effect of *Itgav* deletion, on the proportion of the cells that adhered to the feeder layer after a 3-h incubation (Figure 2L). We also performed short-term *in vivo* assays to examine if there was any defect in their homing potential. Total E14.5 FL-derived cells were transplanted in lethally irradiated animals and the proportion of colony-forming unit cells that homed in the BM within 16 h of transplantation was quantified (Figure 2M). Comparison showed no effect of loss of *Itgav* on the homing potential of the FL-derived hematopoietic stem and progenitor population.

### Loss of *Itgav* in FL HSCs Results in Poor Long-Term Engraftment

**Figure 3:**
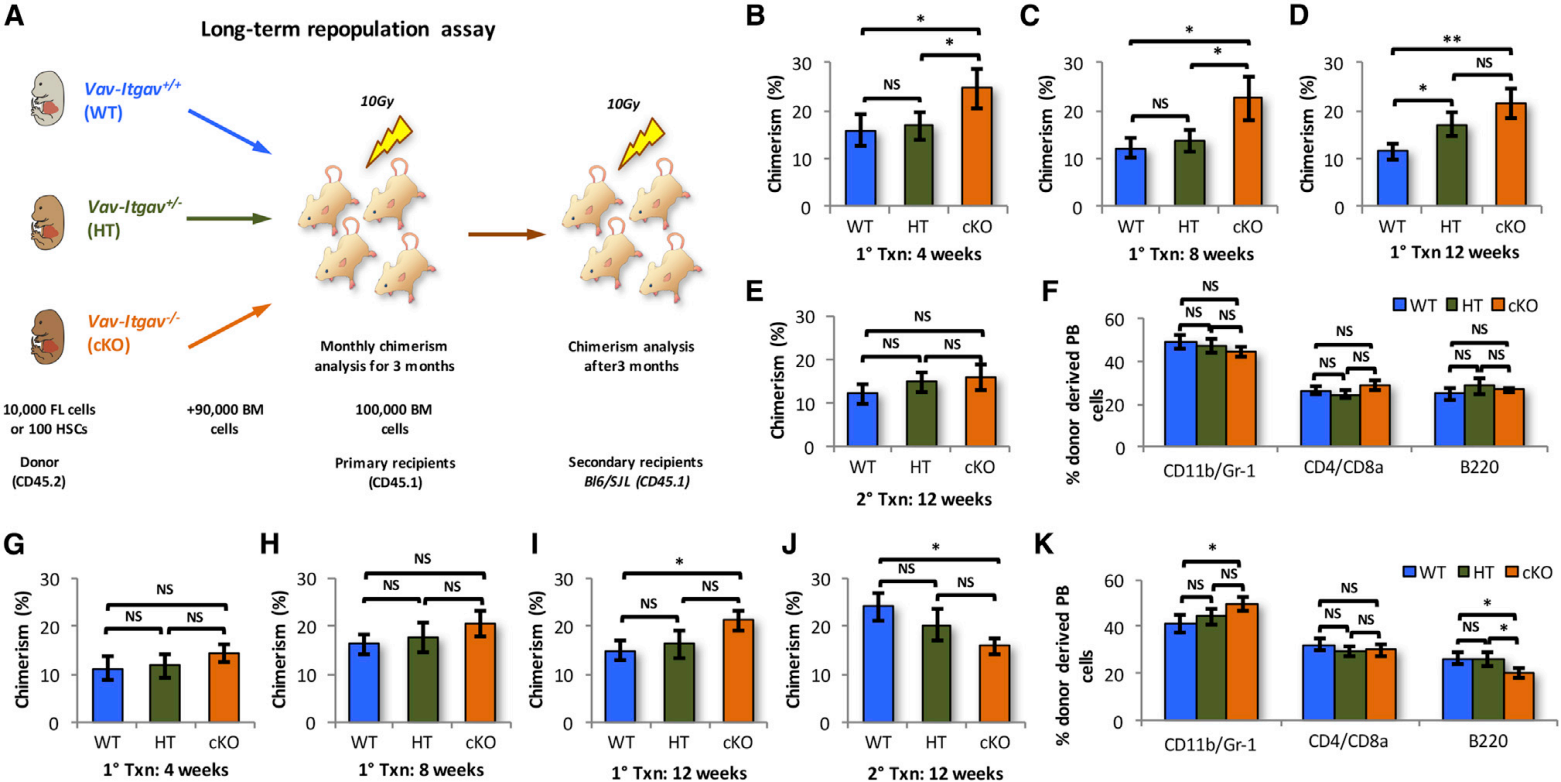
*Vav-Itgav*^*−/−*^ HSCs Acquire Adult Phenotype During Engraftment and Show Functional Decline (A) Schematic representation of the competitive repopulation assay performed to assess the function and/or frequency of HSCs in FL tissue following *Itgav* deletion. A total of 10,000 whole FL cells from WT, HT, and cKO embryos along with 90,000 whole BM competitor cells (CD45.1) were transplanted into lethally irradiated WT CD45.1 mice. Alternatively, the assays were performed using sorted primitive HSCs. One hundred CD150^+^CD48^−^KLS cells together with 100,000 whole BM competitor cells (CD45.1) were transplanted into sub-lethally irradiated WT CD45.1 mice. Peripheral blood (PB) chimerism and multi-lineage engraftment was followed for 12 weeks, after which secondary transplantations were performed. Secondary recipients were analyzed after 3 months of transplantation. (B–D) PB chimerism in primary recipients compared after 4 weeks (B), 8 weeks (C), and 12 weeks (D). (E) PB chimerism in secondary recipients after 12 weeks of transplantation. (F) Multi-lineage engraftment for myeloid (CD11b/Gr-1), T cell (CD4/CD8), and B cell (B220) lineage in secondary recipients after 12 weeks of transplantation. (G–I) Chimerism analysis of primary recipients transplanted with 100 sorted HSCs from WT, HT, and KO embryos, together with 100,000 competitor cells. PB chimerism was analyzed after (G) 4 weeks, (H) 8 weeks, and (I) 12 weeks of transplantation. (J) Chimerism analysis of the secondary recipients transplanted with the BM cells of primary recipients that received freshly sorted HSCs. PB chimerism was analyzed after 12 weeks of transplantation. (K) Multi-lineage engraftment in secondary recipients after 12 weeks of transplantation. Analysis was performed for myeloid (CD11b/Gr-1), T cell (CD4/CD8), and B cell (B220) lineages. An unpaired two-tailed Student's t test was performed. n = 3, N = 15–18, t test: ^∗^p < 0.05, ^∗∗^p ≤ 0.01, NS, not significant.

After confirming that *Itgav* deficiency led to an increase in the frequency of phenotypic HSCs in FL tissue, we examined their functional properties using competitive repopulation assays. First, 10,000 WT, HT, or cKO FL MNCs together with 90,000 supporting CD45.1 whole BM cells were transplanted into lethally irradiated CD45.1 recipients (Figure 3A). The primary recipients that were transplanted with *Vav-Itgav*^*−/−*^ FL cells consistently showed increased chimerism compared with the animals that received WT cells (Figures 3B–3D). Monoallelic deletion of *Itgav* did not result in any change in donor-derived chimerism 8 weeks after transplantation (Figures 3B and 3C). After 12 weeks of transplantation, there was no difference in the level of chimerism between animals that received *Vav-Itgav*^*−/−*^ or *Vav-Itgav*^*+/−*^ FL cells (Figure 3D). To test the long-term engraftment potential, we transplanted BM cells from primary recipients into lethally irradiated secondary recipients. Surprisingly, 12 weeks after secondary transplantation, we did not observe any differences between the donor-derived chimerism in animals that received BM cells from primary recipients of the WT, HT, or cKO cells (Figure 3E). We did not observe any significant change in the multi-lineage engraftment potential following *Itgav* deletion (Figure 3F). Thus, the results from *in vivo* repopulation studies indicated an increase in the frequency of only short-term repopulating FL HSCs resulting from the loss of *Itgav*.

To test the function of the HSC population in response to *Itgav* deletion, we repeated the long-term repopulation assays using sorted HSCs. We transplanted 100 fluorescence-activated cell sorting (FACS)-sorted FL HSCs (CD150^+^CD48^*−*^ LSK cells) from *Vav-Itgav*^*+/+*^, *Vav-Itgav*^*+/−*^, or *Vav-Itgav*^*−/−*^ fetuses, together with 100,000 CD45.1 WBMCs cells into lethally irradiated CD45.1 recipient mice (Figure 3A). No differences were observed among the three groups of primary recipients for up to 8 weeks of transplantation (Figures 3G and 3H). However, there was a significant increase in the donor-derived chimerism from *Vav-Itgav*^*−/−*^ FL HSCs at 12 weeks after transplantation in primary recipients (Figure 3I). After 12 weeks of transplantation, 10 × 10^5^ BM cells from the primary recipients were transplanted into lethally irradiated secondary recipients. Interestingly, we observed that donor-derived chimerism was lower in the cohort that received BM cells from primary recipients transplanted with the *Vav-Itgav*^*−/−*^ HSCs (Figure 3J). These results indicated that the FL HSCs showed poor long-term engraftment in the absence of *Itgav* expression, consistent with our previously published findings on adult BM HSCs (Khurana et al., 2016). Importantly, multi-lineage engraftment potential was also affected in HSCs; however, only following biallelic deletion of *Itgav* (Figure 3K). While there was an increase in the myeloid population within the donor-derived peripheral blood (PB) cells, there was an almost proportionate decrease in the cells from B cell lineage. We observed no difference in the donor-derived contribution to T cell differentiation (Figure 3K).

### Systemic Deletion of *Postn* Leads to Expansion of Functional HSCs in FL

**Figure 4:**
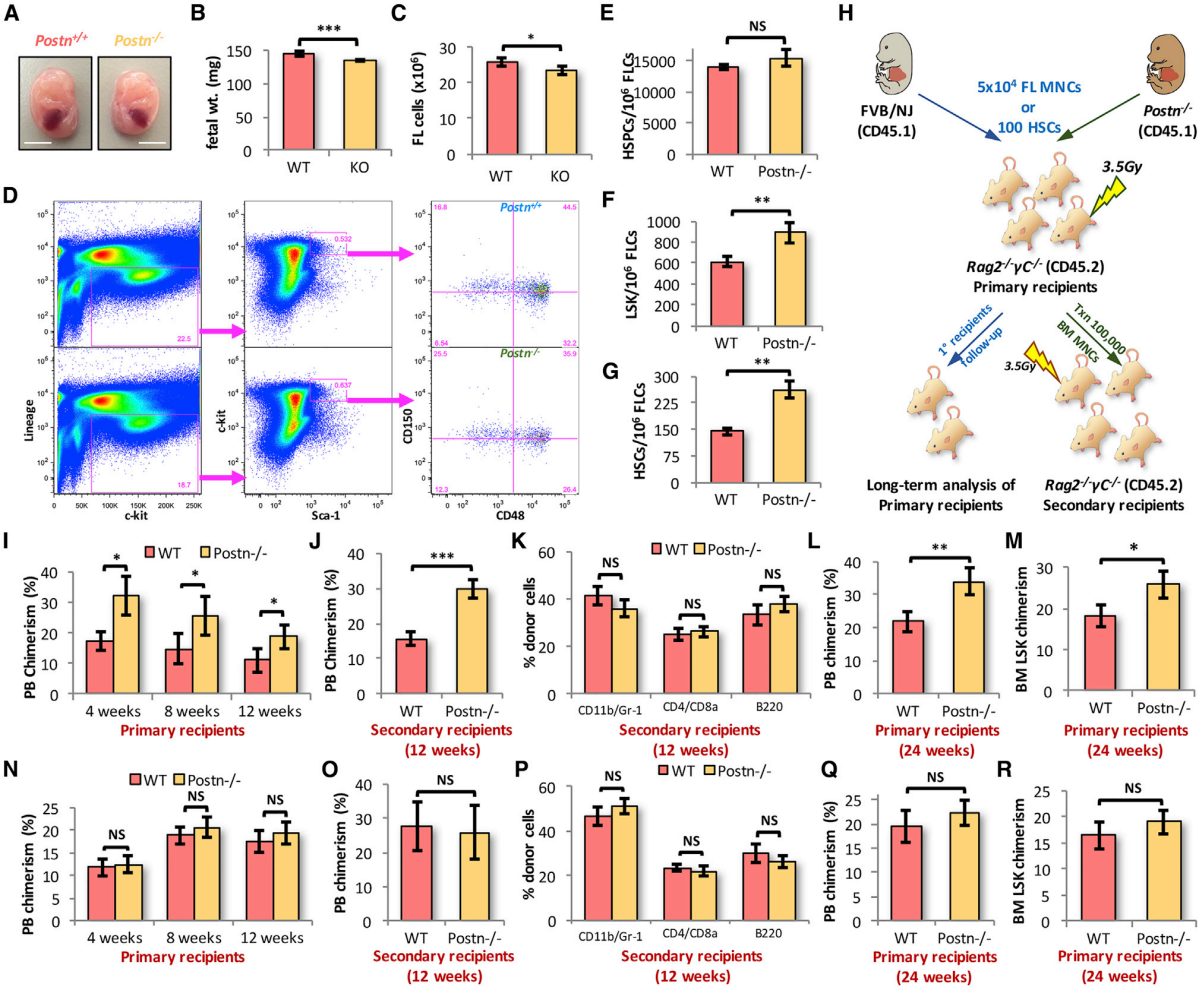
POSTN Deficiency Leads to Expansion of Functional HSCs in FL Tissue (A) *Postn*^*+/+*^ (WT) and *Postn*^*−/−*^ (KO) embryos were harvested at E14.5, cleared of the extra-embryonic layers and gross morphology was compared. Scale bars, 5 mm. (B) Individual embryos were weighed and overall weight of the WT and KO embryos was compared. (C) Mononuclear cells from the E14.5 FL tissues were harvested and counted for the WT and KO embryos. (D–G) The mononuclear cells harvested from the FL tissue of WT and KO embryos were analyzed for the frequency of HSC sub-populations (D) by flow cytometry. The frequency of lin^*−*^c-kit^+^ cells (E), LSK cells (F), and primitive HSCs (G) in E14.5 FL mononuclear cells was compared between WT and *Postn*^*−/−*^ embryos. n = 6–13. (H) Schematic representation of the competitive repopulation assays. A total of 50,000 total E14.5 FL cells or 100 E14.5 FL-derived FACS-sorted SLAM LSK cells from *Postn*^*+/+*^ (WT) or *Postn*^*−/−*^ embryos (CD45.1) was transplanted into sub-lethally irradiated *Rag2*^*−/−*^*γC*^*−/−*^ mice (CD45.2). PB chimerism was followed for 12 weeks, after which secondary transplantations were performed using BM cells from half of the primary recipients. The secondary recipients were analyzed for PB chimerism after 12 weeks of transplantation. The PB chimerism in the rest of the primary recipients was analyzed for up to 24 weeks of transplantation. Donor-derived chimerism in BM LSK population was analyzed in moribund mice. n = 3, N = 15–18. (I) Donor-derived PB chimerism in primary recipients analyzed after 4, 8, and 12 weeks of transplantation. (J) After 12 weeks of transplantation of BM cells from primary recipients, secondary recipients were analyzed for PB chimerism. (K) Multi-lineage engraftment in secondary recipients after 12 weeks of transplantation. (L) After 24 weeks of transplantation, donor-derived PB chimerism in primary recipients that received whole FL cells from WT or KO embryos. (M) The primary recipients analyzed after 24 weeks of transplantation for donor-derived chimerism in BM LSK population. Proportion of BM-derived cells in the total BM LSK cells was compared between the primary recipients that received whole FL cells from E14.5 WT or KO embryos (n = 3, N = 9–12). (N) PB chimerism in primary recipients transplanted with 100 SLAM LSK cells from the E14.5 FL tissues. (O) Secondary recipients analyzed for donor-derived PB chimerism 12 weeks after transplantation. (P) Donor-derived multi-lineage engraftment in secondary recipients 12 weeks after transplantation. (Q) Sorted HSCs from E14.5 WT or KO embryos were transplanted in sub-lethally irradiated animals. After 24 weeks of transplantation, donor-derived PB chimerism in primary recipients was compared between the two groups (n = 3, N = 7–11). (R) The primary recipients analyzed after 24 weeks of transplantation for donor-derived chimerism in BM LSK population. Proportion of BM-derived cells in the total BM LSK cells was compared between the primary recipients that received freshly sorted HSCs from E14.5 WT or KO embryos (n = 3, N = 9–12). An unpaired two-tailed Student's t test was performed. n = 3, N = 15–18, t test: ^∗^p < 0.03, ^∗∗^p ≤ 0.01, ^∗∗∗^p ≤ 0.005, NS indicates not significant. See also Figure S4.

The fetal HSCs transition to an adult phenotype within a few weeks after engraftment, masking any effects of *Itgav* deletion on fetal HSCs. Therefore, we examined the effects of loss of POSTN-ITGAV interaction on HSC function using cells from *Postn*^*−/−*^ FL tissues. While, we did not observe any major developmental defects upon *Postn* deletion at E14.5, we did observe a modest decrease in the overall size of the embryos (Figure 4A). This was also reflected in a marginal, but significant decrease in the weight of *Postn*-deficient fetuses (KO) at E14.5 (Figure 4B). Before the characterization of the hematopoietic system in *Postn*^*−/−*^ FL tissues, we confirmed the lack of *Postn* at transcript and protein levels using qRT-PCR (Figure S4A) and immunohistochemistry (Figure S4B), respectively. We could not detect *Postn* expression in the FL tissue either at the transcript or at the protein level. We observed a modest decrease in the total cellularity of *Postn*^*−/−*^ FL tissue as compared with the WT (Figure 4C). Thereafter, we examined if there was any effect of *Postn* deletion on lineage-committed cell populations (Figures S4C–S4F). We observed no significant change in the proportion of granulocyte (Gr-1^+^; Figure S4C), macrophage (F4/80^+^; Figure S4D), B cell (B220^+^; Figure S4E), or T cell (CD3e^+^; Figure S4F) populations in the FL tissues from *Postn*^*−/−*^ fetuses. We next tested if there was any effect of *Postn* deficiency on the frequency of HSCs in the FL tissue (Figures 4D–4G). We did not observe any difference in the number of lin^−^c-kit^+^ HSPC population between WT and KO fetuses (Figure 4E). However, analysis showed increase in the frequency of LSK cells (Figure 4F) as well as the primitive HSCs (Figure 4G). As there was a modest change in the FL cellularity, we also analyzed the total number of cells in each of these populations, namely lin^−^c-kit^+^ HSPCs (Figure S4G), LSK (Figure S4H), and HSCs (Figure S4I), and observed similar effects upon *Postn* deletion.

To assess hematopoietic function, we performed *in vivo* repopulation assays (Figure 4H). Due to non-availability of suitable CD45.2 congenic mouse line in FVB/NJ background, immune-deficient *Rag2*^*−/−*^*γC*^*−/−*^ mice were used as recipients. Fifty thousand E14.5 FL-derived cells were transplanted into sub-lethally irradiated *Rag2*^*−/−*^*γC*^*−/−*^ mice. No competitor cells were transplanted, as the residual hematopoietic cells (CD45.2^+^ cells against CD45.1^+^ donor-derived cells) following a sub-lethal dose of radiation provided competition to the donor cells. The primary recipients that received FL cells from *Postn*^*−/−*^ embryos showed higher levels of donor-derived chimerism (Figures 4I and S4J). After 12 weeks, the BM cells from half of the primary recipients were transplanted into sub-lethally irradiated secondary recipients and the donor-derived chimerism was followed for another 3 months. We observed a similar increase in donor-derived chimerism in secondary recipients as well (Figure 4J). However, we did not observe any change in the multi-lineage engraftment of the transplanted cells (Figure 4K). There was no observable difference in the proportion of cells from myeloid, T and B cell lineage within the donor-derived PB cells (Figure 4K). Analyses were continued for the second half of the primary recipients for long-term engraftment analysis. We observed a similar increase in donor-derived PB chimerism in the animals that received FL cells from *Postn*^*−/−*^ embryos (Figure 4L). We also examined the donor-derived chimerism in the BM LSK population in moribund mice (Figures 4M and S4K). On expected lines, we observed a higher proportion of donor-derived BM LSK cells in the KO FL cell-transplanted group, although less pronounced than PB chimerism.

To confirm if the increased repopulation potential was due to increased hematopoietic function or HSC frequency, we performed long-term repopulation assays using sorted HSCs (Figure 4H). One hundred FACS-sorted primitive HSCs from WT or *Postn*^*−/−*^ FL tissues were transplanted into sub-lethally irradiated *Rag2*^*−/−*^*γC*^*−/−*^ mice. The results showed no change in donor-derived chimerism in primary (Figure 4N) or secondary recipients (Figure 4O) that were transplanted with HSCs from *Postn*^*−/−*^ FL tissues as compared with WT. Importantly, multi-lineage engraftment potential of the transplanted HSCs also remained intact (Figure 4P). To assess the long-term repopulation potential of the HSCs from *Postn*-deficient FL tissues, we followed the primary recipients for up to 24 weeks. As indicated by the PB chimerism after 24 weeks, we did not observe any effect of *Postn* deletion on the long-term repopulation potential of the FL HSCs (Figure 4Q). In addition, we observed no change in the donor-derived chimerism in the BM LSK cell population after 24 weeks of transplantation (Figure 4R). These results indicated that the HSCs did not differ in their repopulation potential. Therefore, the increased level of chimerism after transplantation of total *Postn*^*−/−*^ FL cells is likely due to increased frequency of HSCs and HSPCs.

### FL HSCs Tolerate Culture-Induced DNA Damage Better Than BM HSCs

**Figure 5:**
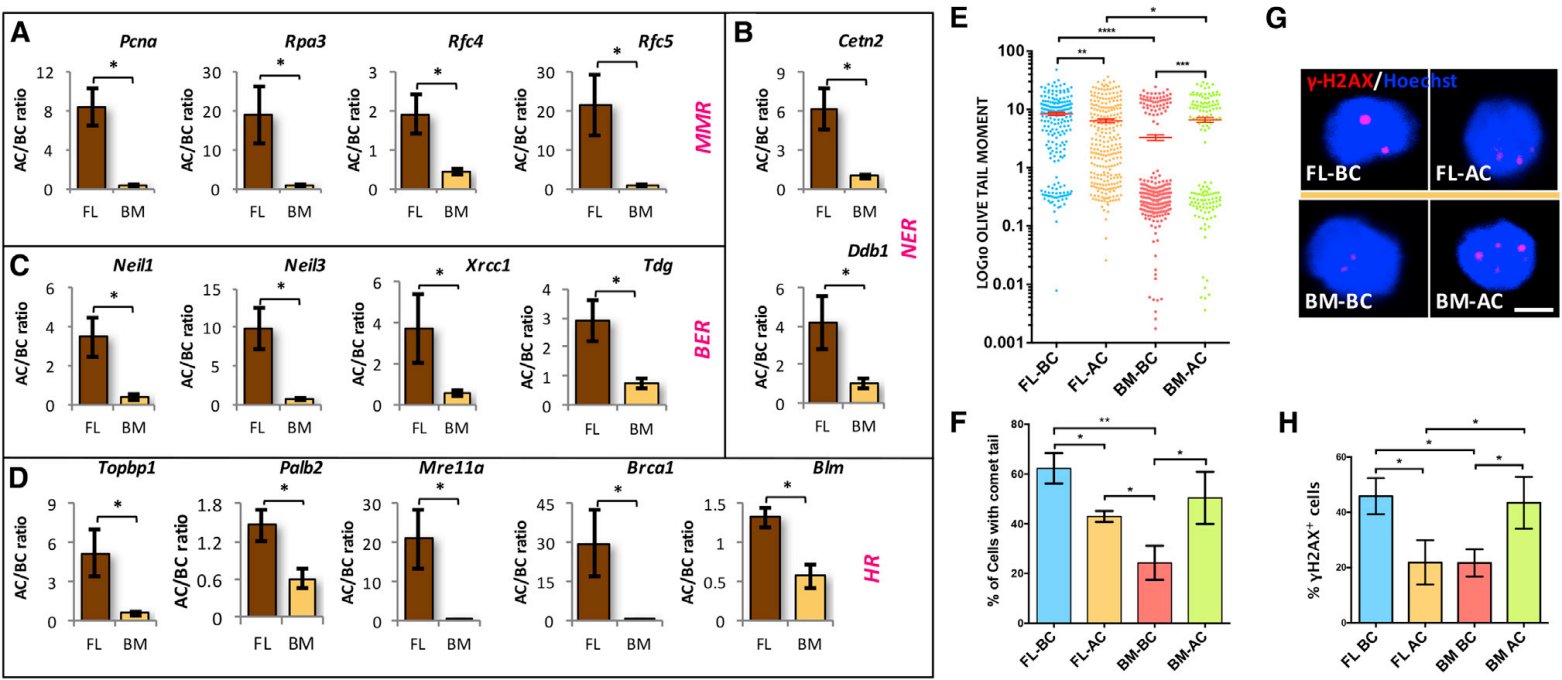
Elevated DNA Damage Response in FL-Derived HSCs against Culture-Induced Stress (A–D) Bone marrow and E14.5 FL-derived KLS cells were cultured in serum-free medium in the presence of SCF and Tpo for 5 days. Expression for DDR genes from the harvested cells (after culture [AC]) was compared with the freshly isolated (before culture [BC]) LSK cells by performing qRT-PCR. Culture-induced change in the expression of genes that belong to different DDR pathways was plotted. (A) mismatch repair, *Pcna*, *Rpa3*, *Rfc4*, *Rfc5*; (B) nucleotide excision repair, *Cetn2*, *Ddb1*; (C) base excision repair, Neil1, Neil3, Xrcc1, Tdg; (D) homologous recombination, *Topbp1*, *Palb2*, *Mre11a*, *Brca1*, *Blm*. n = 4–6, t test: ^∗^p < 0.05. (E and F) Neutral comet assay was performed using freshly isolated and cultured KLS cells from BM and FL tissues. Olive tail moment (E) and proportion of cells with comet tail (F) was compared for different samples. n = 4, N > 223, one-way ANOVA followed by Tukey's multiple comparisons test: ^∗^p < 0.05, ^∗∗^p ≤ 0.01, ^∗∗∗^p ≤ 0.005. (G) Immunostaining for γ-H2AX was performed on freshly isolated and cultured LSK cells from BM and E14.5 FL tissues. Scale bar, 5 μm. (H) Proportion of cells with γ-H2AX for was compared between different samples. n = 4, N > 173, one-way ANOVA followed by Tukey's multiple comparisons test: ^∗^p < 0.05, ^∗∗^p ≤ 0.01. See also Figure S5.

We hypothesized that the intrinsic differences between fetal and adult HSCs were the underlying reason for the difference in their tolerance to proliferation stress. Interestingly, from our earlier published RNA-seq data, we observed that mismatch repair (MMR), homologous recombination (HR), nucleotide excision repair (NER), and base excision repair (BER) were among the top 20 highly expressed pathways in E14.5 FL-derived HSCs compared with the adult BM-derived HSCs (Manesia et al., 2015). Heatmap analysis also showed increased expression levels of genes involved in MMR (Figure S5A), HR (Figure S5B), NER (Figure S5C), and BER (Figure S5D). Next, we aimed to examine the functional relevance of these pathways in the repair of culture-induced DNA damage (Figure 5). We sorted LSK cells from E14.5 FL as well as adult BM and cultured them in serum-free medium along with stem cell factor (SCF) and thrombopoietin for 5 days. This expansion medium induces proliferation of HSCs as reported previously (Khurana et al., 2013b; Sitnicka et al., 1996; Zhang and Lodish, 2005) and expectedly; we observed faster proliferation rates in FL-derived LSK cells (Figure S5E). The proliferative events also led to increase in the single- and double-strand DNA breaks, which are repaired during the replication process. We explored if the LSK cells isolated from FL and BM showed differences in the expression of key genes involved in DDR pathways before and after culture-induced proliferation (Figures 5A–5D). We observed clear upregulation of DDR pathway genes in FL HSCs upon culture. Among the genes involved in the MMR pathway, we observed increased expression of *Pcna*, *Rpa3*, *Rfc4*, and *Rfc5* (Figure 5A). From the NER pathway, *Cetn2* and *Ddb1* (Figure 5B), and from the BER pathway, *Neil1*, *Neil3*, *Xrcc1*, and *Tdg* (Figure 5C), showed similar increase in expression. Among HR-related genes, we tested *Topbp1*, *Palb2*, *Mre11a*, *Brca1*, and *Blm*, and all showed significantly higher levels of expression (Figure 5D). Importantly, when we examined BM-derived LSK cells for DDR gene expression following culture, the increase in the expression of all these DDR genes was significantly lower than what was observed for FL-derived cells.

Gene expression may not clearly reflect the status of functional activity of the repair pathways tested; therefore, we next examined if there was any improvement in DNA damage repair in these cells. Freshly sorted as well as cultured BM- and FL-derived LSK cells were used to perform the neutral comet assay (Figures 5E and 5F) to measure double-strand breaks. Olive tail moment is a measure of DNA damage that takes into consideration both the length of the comet tail and the proportion of total DNA it contains. We observed lower levels of DNA damage in freshly isolated BM-derived cells than their FL counterparts, likely due to higher proliferation rates in FL-derived cells. In contrast, the olive tail moment was significantly decreased when FL-derived LSK cells were cultured for a period of 5 days (Figure 5E). Contrary to this, we observed an increase in double-strand breaks in BM LSK cells upon culture. We also examined the proportion of cells with a comet tail and observed similar results (Figure 5F). The proportion of FL-derived LSK cells with comet tail decreased after 5 days of culture while the BM-derived LSK cells showed a significant increase, indicating functional differences in DDR pathways. We confirmed these observations by performing immunostaining for γ-H2AX, a marker for DNA double-strand breaks (Figure 5G). Both the proportion of cells with γ-H2AX foci (Figure 5H) and number of the γ-H2AX foci per cell (Figure S5F) decreased in FL-derived cells after culture. BM-derived HSPCs on the other hand accumulated more DNA damage per cell. The results also showed that the proportion of cells with a higher number of foci (3–5 and >5) increased significantly when BM-derived cells were cultured (Figure S5G). Contrary to this, the DNA damage was more efficiently cleared upon culture of FL-derived HSPCs. These experiments confirmed an increased level of DDR activity in FL-derived HSPCs than BM.

### Better DDR in HSCs from *Postn*^*−/−*^ FL Tissues

**Figure 6:**
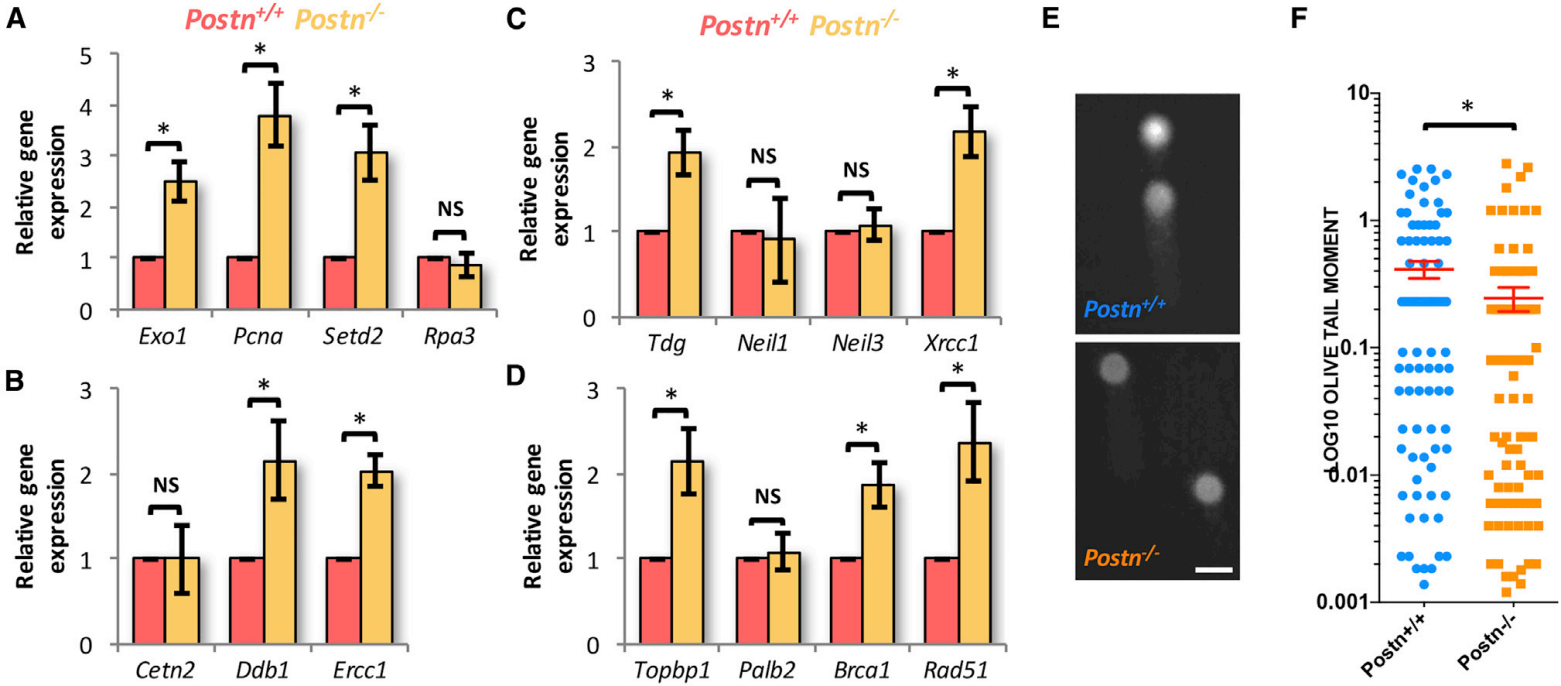
Better DNA Damage Repair in HSCs from *Postn*^*−/−*^ FL Tissues (A–D) *Postn*^*+/+*^ and *Postn*^*−/−*^ FL-derived LSK cells were used for assessing the expression of DNA damage response genes through qRT-PCR. Expression of genes that belong to MMR, such as *Exo1*, *Pcna*, *Setd2*, and *Rpa3* (A), NER, such as *Cetn2*, *Ddb1*, and *Ercc1* (B), BER, such as *Tdg*, *Neil1*, *Neil3*, and *Xrcc1* (C), and HR pathways, such as *Topbp1*, *Palb2*, *Brca1*, and *Rad51* (D) was examined. Gene transcript changes were normalized to *β-actin* levels and relative expression of respective genes was examined in LSK cells from *Postn*^*−/−*^ FL tissues as compared with the *Postn*^*+/+*^ FL-derived cells. Unpaired two-tailed Student's t test. n = 4–6. ^∗^p < 0.0055. (E and F) Freshly sorted primitive HSCs (CD150^+^CD48^*−*^ LSK cells) from E14.5 *Postn*^*+/+*^ and *Postn*^*−/−*^ FL tissues were used to perform neutral comet assay. (E) Representative comets from the HSCs from *Postn*^*+/+*^ (upper panel) and *Postn*^*−/−*^ (lower panel) FL tissues. Scale bar, 10 μm. (F) Olive tail moment compared between *Postn*^*+/+*^ (N = 379) and *Postn*^*−/−*^ (N = 307) FL HSCs. n = 4–6, Mann-Whitney U test: ^∗^p = 0.0407.

Our results demonstrated that the proliferative FL HSCs were significantly better in their DDRs. These intrinsic differences resulted in increased levels of tolerance to proliferative stress in fetal HSCs as compared with their adult BM equivalents. We hypothesized that the expansion of functional HSCs in the FL tissues of *Postn*-deficient embryos could also be linked to better DNA damage repair. To test this hypothesis, we first checked the expression of some of the DDR genes, earlier identified in our screen, where HSCs isolated from FL and adult BM were compared (Figures S5A–S5D). We sorted LSK cells from FL tissues of *Postn*^*+/+*^ and *Postn*^*−/−*^ fetuses and performed qRT-PCR to examine the expression of genes that belong to MMR (*Exo1*, *Pcna*, *Setd2*, and *Rpa3*; Figure 6A), NER (*Cetn2*, *Ddb1*, and *Ercc1*; Figure 6B), BER (*Tdg*, *Neil1*, *Neil3*, and *Xrcc1*; Figure 6C), and HR pathways (*Topbp1*, *Palb2*, *Brca1*, and *Rad51*; Figure 6D). Some of these genes (*Rpa3* from MMR; *Cetn2* from NER; *Neil1*and *Neil3* from BER; and *Palb2* from HR) did not show any change in expression upon *Postn* deletion. However, several of them from MMR (*Exo1*, *Pcna*, and *Setd2*; Figure 6A), NER (*Ddb1* and *Ercc1*; Figure 6B), BER (*Tdg* and *Xrcc1*; Figure 6C), and HR pathways (*Palb2*, *Brca1*, and *Rad51*; Figure 6D) showed significant increase in LSK cells sorted from *Postn*^*−/−*^ FL tissues. These results indicated a possible functional improvement in DNA damage repair. To test this, we performed neutral comet assays using HSCs (SLAM LSK cells) from *Postn*^*+/+*^ and *Postn*^*−/−*^ FL tissues (Figures 6E and 6F). Comparison of the olive tail moment of individual cells showed clear decrease in the DNA damage in the HSCs derived from *Postn*^*−/−*^ FL tissues (Figure 6F; n = 3, N = 105–132, ^∗^p = 0.04). We concluded that, during embryonic development, proliferative stress is tolerated by increased levels of DDRs.

### POSTN Is Expressed in Vascular Endothelium of the FL

**Figure 7:**
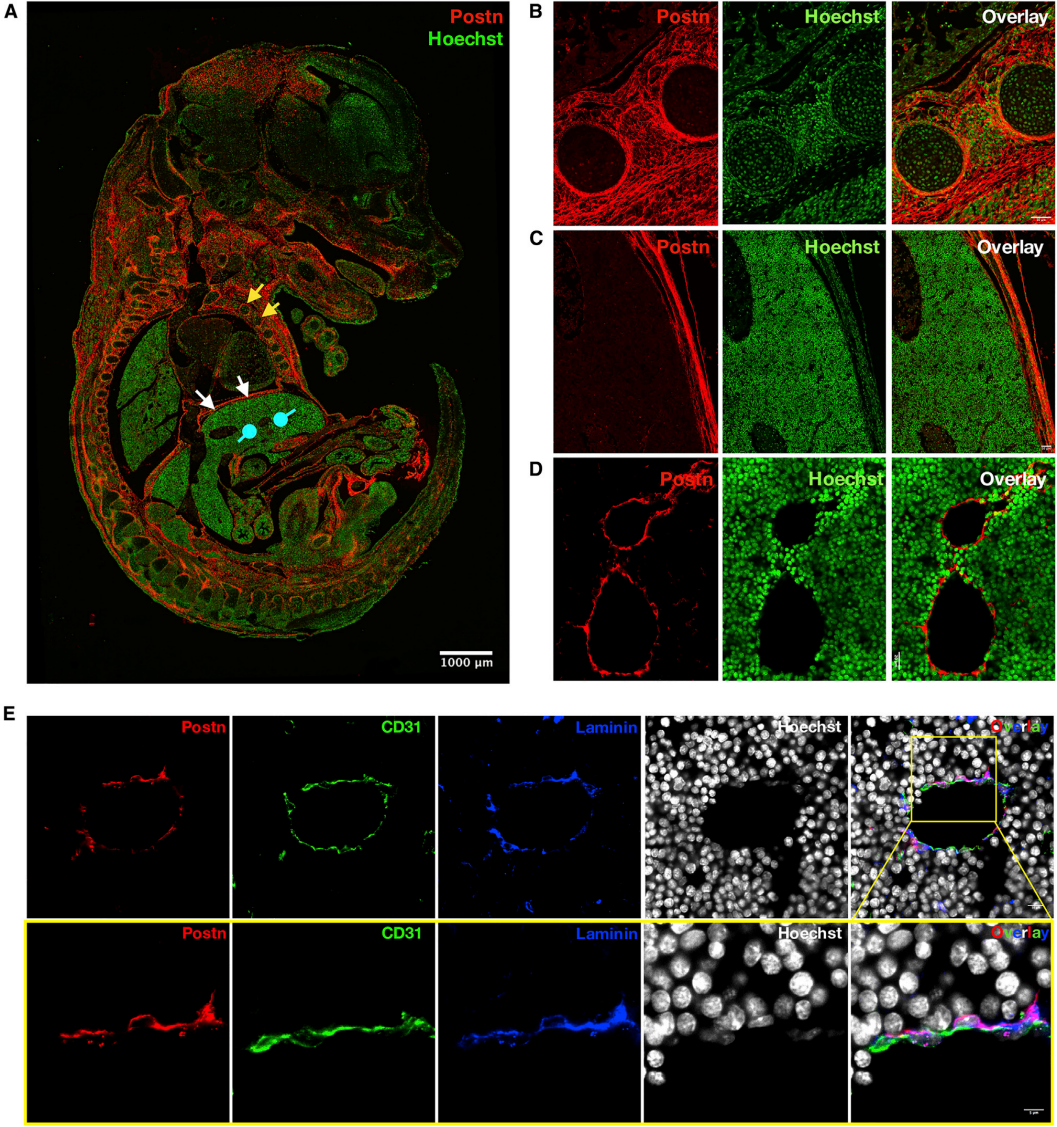
POSTN Is Expressed in Vascular Endothelium of the FL (A) PSTN expression was examined in the E14.5 fetuses using immunohistochemistry on 10-μm formalin-fixed paraffin-embedded sections followed by confocal imaging. Specific antibodies were used to identify the cells expressing POSTN, nuclear counterstaining was done using Hoechst 33342. Regions with compelling levels of POSTN expression; namely, the developing ribs (yellow arrows), diaphragm (white arrows), and the tissue of interest, FL (blue round head arrows), are indicated (n = 4). Scale bar, 1 mm. (B–D) Enlarged POSTN-expressing regions from the tiles were extracted. (B) Developing ribs showing marrow as well as the mesenchymal tissue (n = 3). Scale bar, 50 μm. (C) Muscular diaphragm (n = 3). Scale bar, 50 μm. (D) Vascular region in the FL tissue (n = 4). Scale bar, 20 μm. (E) Immuno-staining of the FL sections with endothelial marker CD31 and ECM protein laminin, along with POSTN. The sections were counterstained with Hoechst 33342 (upper panel) (n = 4). Scale bar, 10 μm. The lower panel shows an enlarged part of the section shown in the upper panel. Scale bar, 5 μm). See also Figure S6.

Results presented above further established POSTN as a negative regulator of HSC proliferation across developmental stages, notwithstanding the impact on their overall stemness. Inhibition of its interaction with ITGAV in the FL led to the expansion of the HSC pool size without any loss of stemness. We investigated if POSTN was involved in the creation of HSC niche in the FL tissue. To determine where POSTN is expressed within the E14.5 FL tissue, immuno-histochemical analysis was performed on 10-μm formalin-fixed paraffin-embedded sections. Confocal imaging clearly showed that POSTN was expressed in most of the skeletal primordial structures linked with bone formation (Figure 7A). Further examination of POSTN expression in specific tissues within the fetal sections revealed high levels of expression in the cartilage primordia of the rib shaft (Figure 7B). In fact, the entire vascular perichondrium surrounding the non-vascular fetal hyaline cartilage expressed POSTN. However, we did not observe any POSTN expression within the developing rib marrow. In addition to the developing ribs, we observed abundant POSTN expression associated with the skeletal muscle fibers of the developing diaphragm that is rich in connective tissue and tendon (Figure 7C). Gross analysis showed that the majority of cells in the FL tissue did not express POSTN (Figure 7A). However, upon detailed examination of the FL tissue, we observed POSTN signals from the vascular lining not restricted to a specific part of the tissue (Figure 7D). Vascular endothelium has been shown to play an important role in creating the HSC niche in the BM as well as in the FL tissue. Therefore, we tested if indeed it was the endothelium associated with the developing hepatic vasculature that expressed POSTN. We used Endoglin and CD31 as endothelial markers and examined the proportion of these cells that expressed POSTN. We analyzed z stack images taken at 0.35-μm step-size to count individual endothelial cells and tested for POSTN expression (Figure S6; Videos S1, S2, S3, and S4). Quantification showed that 91.29% ± 3.07% of Endoglin^+^ (Figure S6A; Video S1 and S2) and 89% ± 2.08% of CD31^+^ cells (Figure S6B; Videos S3 and S4) also expressed POSTN (n = 3, N = 271–300). We detected POSTN expression in some non-endothelial cells also, but vascular endothelium was clearly the most prominent cell type with POSTN expression. Careful observations showed that at least a part of the non-endothelial POSTN signals appeared non-cellular. As POSTN is a known ECM binding protein, we examined if POSTN showed co-localization with laminin associated with basal lamina (Figure 7E; Video S5). It was clear that CD31^+^ endothelial cells expressed POSTN, although both signals were spatially distinct (Figure 7E, lower panel; Video S6). Importantly, POSTN signals showed clear overlap with laminin, associated with basal lamina. Overall, we show that POSTN is a potential component of HSC niche in the FL tissues and acts as a negative regulator of proliferation in otherwise proliferative HSCs.

Video S1. z Stack Series and Maximum Projection of Confocal Images Showing Postn Expression in Endoglin Expressing Vascular Endothelial Cells, Related to figure 6 Postn in red, endoglin in green, and Hoechst 33342 in blue. Scale bar, 10 μm

Video S2. z Stack Series and Maximum Projection of Zoomed-in Confocal Images Showing Postn Expression in Endoglin Expressing Vascular Endothelial Cells, Related to Figure 6 Postn in red, endoglin in green, and Hoechst 33342 in blue. Scale bar, 10 μm

Video S3. z Stack Series and Maximum Projection of Confocal Images Showing Postn Expression in CD31 Expressing Vascular Endothelial Cells, Related to Figure 6 Postn in red, CD31 in green, and Hoechst 33342 in blue. Scale bar, 10 μm.

Video S4. z Stack Series and Maximum Projection of Zoomed-in Confocal Images Showing Postn Expression in CD31 Expressing Vascular Endothelial Cells Postn in red, CD31 in green, and Hoechst 33342 in blue. Scale bar, 10 μm.

Video S5. z Stack Series and Maximum Projection of Confocal Images Showing Postn Co-localization with ECM Protein Laminin around CD31 Expressing Vascular Endothelial Cells, Related to Figure 6 Postn in red, CD31 in green, laminin in blue, and Hoechst 33342 in white. Scale bar, 10 μm. Related to Figure 6

Video S6. z Stack Series and Maximum Projection of Zoomed-in Confocal Images Showing Postn Co-localization with ECM Protein Laminin around CD31 Expressing Vascular Endothelial Cells, Related to Figure 6 Postn in red, CD31 in green, laminin in blue, and Hoechst 33342 in white. Scale bar, 10 μm.

## Discussion

We earlier demonstrated that POSTN-ITGAV interaction promotes quiescence in adult BM HSCs (Khurana et al., 2016). Here, we present evidence that systemic loss of *Postn* results in increased proliferation of FL HSCs. It has been well documented that the HSCs in FL tissue proliferate extensively (Morrison et al., 1995). In addition, upon transplantation in lethally irradiated animals competitive repopulation units (CRUs) from E14.5 FL show more robust engraftment than BM CRUs, along with a much higher clonal expansion (Rebel et al., 1996). Our studies confirm the observation that significantly fewer FL HSCs are quiescent (in the G0 stage of the cell cycle) compared with adult BM HSCs. The fraction of FL HSCs in S and G2M phases was increased in *Vav-Itgav*^*−/−*^ embryos, perhaps indicating faster G1 progression. Long-term repopulation assays demonstrated that, in contrast to the BM HSCs from *Postn*^*−/−*^ mice, enhanced proliferation of FL HSCs did not affect their function. Hence, enhanced proliferation of FL HSCs resulted in de facto expansion rather than functional decline. This was reflected clearly in their robust multi-lineage engraftment potential, even upon transplantation into secondary recipients.

It has been demonstrated that HSCs keep proliferating postnatally to create a pool of HSCs that take up newly formed BM niches (Bowie et al., 2006). In mice, transition from a proliferative to quiescent phenotype takes place 3–4 weeks after birth. Detailed experiments later showed that E14.5 FL-derived HSCs, transplanted in irradiated hosts for 4–6 weeks, were indistinguishable from adult BM HSCs (Bowie et al., 2007). As the proliferative HSCs from FL transit to a quiescent state after transplantation, the phenotype due to lack of *Itgav* will be due to the changes in functioning of the adult HSCs. Due to intrinsic genetic alteration, true reflection of functional change specifically at fetal stage will be masked. Therefore, a snapshot of the functional status of HSCs after interruption of POSTN-ITGAV interaction was possible only by using *Postn*^*−/−*^ FL HSCs. It could be seen through the long-term repopulation assays performed using total FL cells that donor-derived chimerism was higher when cells from *Postn*^*−/−*^ fetuses were transplanted. Further studies using sorted HSCs confirmed that the HSCs from *Postn*^*−/−*^ FL tissues did not differ in their engraftment potential. Put together, *in vivo* repopulation assays confirmed an overall expansion of HSCs in FL, wherein POSTN-ITGAV axis was disrupted. This was unlike their BM counterparts, where a loss of function upon entry into the cell cycle was observed; indicating intrinsic differences between the stem cell populations from different developmental stages. In fact, the *in vivo* engraftment assays using *Vav-Itgav*^*−/−*^ FL HSCs confirmed our earlier findings. We observed poorer engraftment, with myeloid skewing, as these cells transited to the adult BM phenotype after transplantation.

Earlier, our group (Manesia et al., 2015) and McKinney-Freeman et al. (2012) reported the transcriptional profile of primitive HSCs from different fetal developmental stages compared with adult BM HSCs. Further evaluation of pathways that were differentially expressed in our RNA-seq dataset identified increased levels of DDR pathway genes in FL HSCs (Figure S5). DNA damage accumulation in HSCs is linked with functional decline during aging (Rossi et al., 2007). In addition, in a number of mouse models with defective DDRs, pre-mature aging of HSCs was observed (Ito et al., 2004). These changes involve increased proliferation of HSCs, poorer engraftment potential, and differentiation skewed toward myelopoiesis at the expense of lymphopoiesis (Rossi et al., 2005). Our results demonstrate that the outcome of increased HSC proliferation is influenced by the developmental context. We observed that *ex vivo* culture of E14.5 FL HSCs was associated with significantly better DNA damage repair than adult HSCs. Not only was there an increased expression of genes belonging to different DDR pathways, but we also observed that the extent of DNA repair was significantly greater in FL than adult BM HSPCs. In line with these findings, we observed increased levels of DNA damage repair genes resulting in lower levels of double-strand breaks in the FL HSCs from *Postn*-deficient embryos. It has clearly been established that DNA damage signaling is well-linked with cell-cycle progression through several check-points (Jackson and Bartek, 2009). Our results indicate that the strength of this signaling might be developmental context dependent. First, a stronger activation of DDR pathways during embryonic development ensures that the proliferation stress is well tolerated. Secondly, a further increase in proliferation rates induced faster repair kinetics in HSCs in *Postn*-deficient embryos resulting in overall HSC expansion without any loss of stemness.

We earlier reported that E14.5 FL HSCs have higher mitochondrial activity resulting in increased levels of reactive oxygen species (ROS) (Manesia et al., 2015). However, this oxidative stress appears to be well tolerated by the fetal HSCs unlike their adult BM counterparts (Wang et al., 2014). The observation that FL HSCs possess enhanced DNA repair capabilities, reported here, may also help explain this contrast. Increased ROS levels are considered to be one of the major factors underlying DNA damage accumulation, ultimately leading to poorly regulated cell-cycle progression (Yahata et al., 2011). Antioxidants have been used to reverse the effects of increased ROS levels in response to increased mitochondrial respiration (Ito et al., 2006).

In addition to providing clues to the genetic basis of functional differences between fetal and adult HSCs, our results also provide key insights into the role of outside-in integrin signaling. The results presented here confirm the role of the POSTN-ITGAV axis in the regulation of cell-cycle progression in HSCs irrespective of the developmental stage. A recent study implicated the POSTN-integrin axis in mediating the effects of the vitamin K antagonist warfarin, used clinically to prevent thromboembolic events (Verma et al., 2019). Warfarin inhibited HSC function through blocking γ-carboxylation of POSTN, decreasing the binding of POSTN to ITGB3. Infusion of carboxylated POSTN rescued animals from warfarin-induced coagulation defects. Expression of ITGB3, an ITGAV binding partner, has been correlated with the quiescent properties and long-term repopulation potential of HSCs (Umemoto et al., 2006). Although, RNA-seq results demonstrate that transcript levels of *Itgav* and *Itgb3* in FL HSCs is lower than in BM HSCs, significant levels of cell surface expression of ITGAV and ITGB3 were observed in E14.5 FL-derived primitive HSCs. There is evidence that the ITGAV-B3 heterodimer can be involved in inside-out signaling, affecting adhesion of cells to ECM, possibly through binding to POSTN (Umemoto et al., 2012). However, we have demonstrated that loss of *Itgav* from adult BM HSCs did not affect their adhesion potential (Khurana et al., 2016).

The availability of modern imaging tools, aided with more accurate reporter mouse lines, contributed to the expansion in our knowledge about the physical location of HSCs in the BM. These tools have unequivocally established the importance of BM vasculature in the physical maintenance of primitive HSCs (Boulais and Frenette, 2015). Even functional decline of HSCs during aging has been attributed to the genetic and physical changes in the vascular niche (Kusumbe et al., 2016). The most crucial of the factors that regulate HSC function have been shown to be expressed within the cellular components of these niches. A variety of cell types associated with different vessel types have been shown to express crucial regulators of adult hematopoiesis (Sasine et al., 2017). Of note, the expression of hematopoietic regulators, such as SCF (Ding et al., 2012) and stromal cell-derived factor-1α (Ding and Morrison, 2013) was used to identify the microenvironment of the most primitive HSCs in the BM. These experiments established the importance of BM vascular niche, not only to physically host the HSCs, but also in regulating their function. Our experiments also showed that POSTN expression is largely restricted to vascular areas in the FL tissue. In fact, recent findings have indicated that HSCs take up vascular niches in the FL (Tamplin et al., 2015). In Zebrafish, endothelial cells assisted the colonization of caudal hematopoietic tissue by the incoming HSCs and this phenomenon was conserved in mouse. Interestingly, at a later stage in mouse FL tissue, it was noted that the Ng2^+^Nestin^+^ pericytes, associated with portal vessels, were a crucial component of the HSC niche (Khan et al., 2016). Overall though, high levels of POSTN expression were observed in skeletal tissues in the developing fetus, such as in the developing ribs and diaphragm, both tissues rich in muscle cells and ECM proteins (Merrell and Kardon, 2013; Quondamatteo et al., 2002). This was in line with the earlier published findings that POSTN is associated with ECM proteins, such as type I collagen (Norris et al., 2007), fibronectin (Takayama et al., 2006), laminin (Nishiyama et al., 2011), as well as skeletal structures.

In conclusion, we present evidence for differential responses of HSCs to enhanced proliferation, such as caused by disruption of the POSTN-ITGAV axis, depending upon their developmental stage. Loss of POSTN in the HSC niche induces proliferation; however, this is associated with functional decline only in the adult BM. By contrast, an expansion of the HSC pool is observed in *Postn*^*−/−*^ FL tissue. We present evidence that fetal HSCs might be less vulnerable to proliferation-induced stress due to increased levels of DNA damage repair.

## Experimental Procedures

### Animals

The animals were bred at the animal facilities of KU Leuven, Rajiv Gandhi Center for Biotechnology, and IISER Thiruvananthapuram. During the experiments, mice were maintained in isolator cages, fed with autoclaved acidified water and irradiated food ad libitum. All animal experiments were approved by the Institutional Animal Ethics Committees for the respective animal facilities, details can be found in Supplemental Information.

### Long-Term Repopulation Assays

A single dose of 3.5 Gy (sub-lethal dose) or 10 Gy (sub-lethal dose) was given to the animals, a day before the test cells were transplanted. Mononuclear cells or the sorted HSCs E14.5 *Vav-Itgav*^*+/+*^, *Vav-Itgav*^*+/−*^, and *Vav-Itgav*^*−/−*^ FL tissue were transplanted along with the competitor cells into lethally irradiated mice. Transplantation of sorted or unsorted FL HSCs from *Postn*^*+/+*^ and *Postn*^*−/−*^ embryos was performed into sub-lethally irradiated *Rag2*^*−/−*^*γC*^*−/−*^ mice, without competitor cells. PB chimerism analysis was performed every 4 weeks after which transplantation into secondary recipients was performed, which was followed for an additional 12 weeks. Further details can be found in Supplemental Information.

### Statistical Analysis

All data are represented as mean ± SEM. Normal distribution of data was tested using the Shapiro-Wilk test. The equality of group variance was tested using the Brown-Forsythe test. Comparisons between samples from two groups with normally distributed data with equal variance were made using the unpaired two-tailed Student's t test. The Mann-Whitney test was used for comparing two groups where data were non-normally distributed. For multiple comparisons of the normally distributed data with equal variance, one-way ANOVA was performed followed by the Tukey-Kramer post hoc test. Non-normally distributed data was analyzed by the Friedman test. The chi-square test was used for testing the goodness of fit of the observed ratios among genotypes of embryos to expected Mendelian ratio. Statistical analyses were performed with Microsoft Excel or GraphPad Prism 6. For all analyses, p values ≤0.05 were accepted as statistically significant, n represents the number of biological repeats and N represents total number of technical repeats across experiments.

## Author Contributions

S.K. conceptualized the study, laid out the experimental design, supervised the project, analyzed data, and wrote the manuscript. A.B., I.M.R., and P.C.B. performed the experiments and analyzed the results. A.B. assisted in writing the manuscript, and critically reviewed the drafts. J.M. performed computational analysis of the RNA-seq data and reviewed manuscript. S.S. and V.V. provided technical assistance. R.J.A. and J.H. provided material. A.L.-H. provided material and reviewed the manuscript. C.M.V. provided material support and critically reviewed the manuscript.

## Supplemental Information

Document S1. Supplemental Experimental Procedures, Figures S1–S6, and Tables S1 and S2

Document S2. Article plus Supplemental Information

## Footnotes

Supplemental Information can be found online at https://doi.org/10.1016/j.stemcr.2020.06.022.
