# Supplementary material for: The Periostin/Integrin-αv Axis Regulates the Size of Hematopoietic Stem Cell Pool in the Fetal Liver
Source: Stem Cell Reports. 2020 Jul 30;15(2):340–57. doi: 10.1016/j.stemcr.2020.06.022 (PMC7419718; doi:10.1016/j.stemcr.2020.06.022)
Supplement: Document S1. Supplemental Experimental Procedures, Figures S1–S6, and Tables S1 and S2 [file mmc1.pdf]

Stem Cell Reports, Volume *15*

Supplemental Information

The Periostin/Integrin-αv Axis Regulates the Size of Hematopoietic Stem Cell Pool in the Fetal Liver

Atreyi Biswas, Irene M. Roy, Prathibha C. Babu, Javed Manesia, Sarah Schouteden, Vinod Vijayakurup, Ruby John Anto, Joerg Huelsken, Adam Lacy-Hulbert, Catherine M. Verfaillie, and Satish Khurana

Figure S1

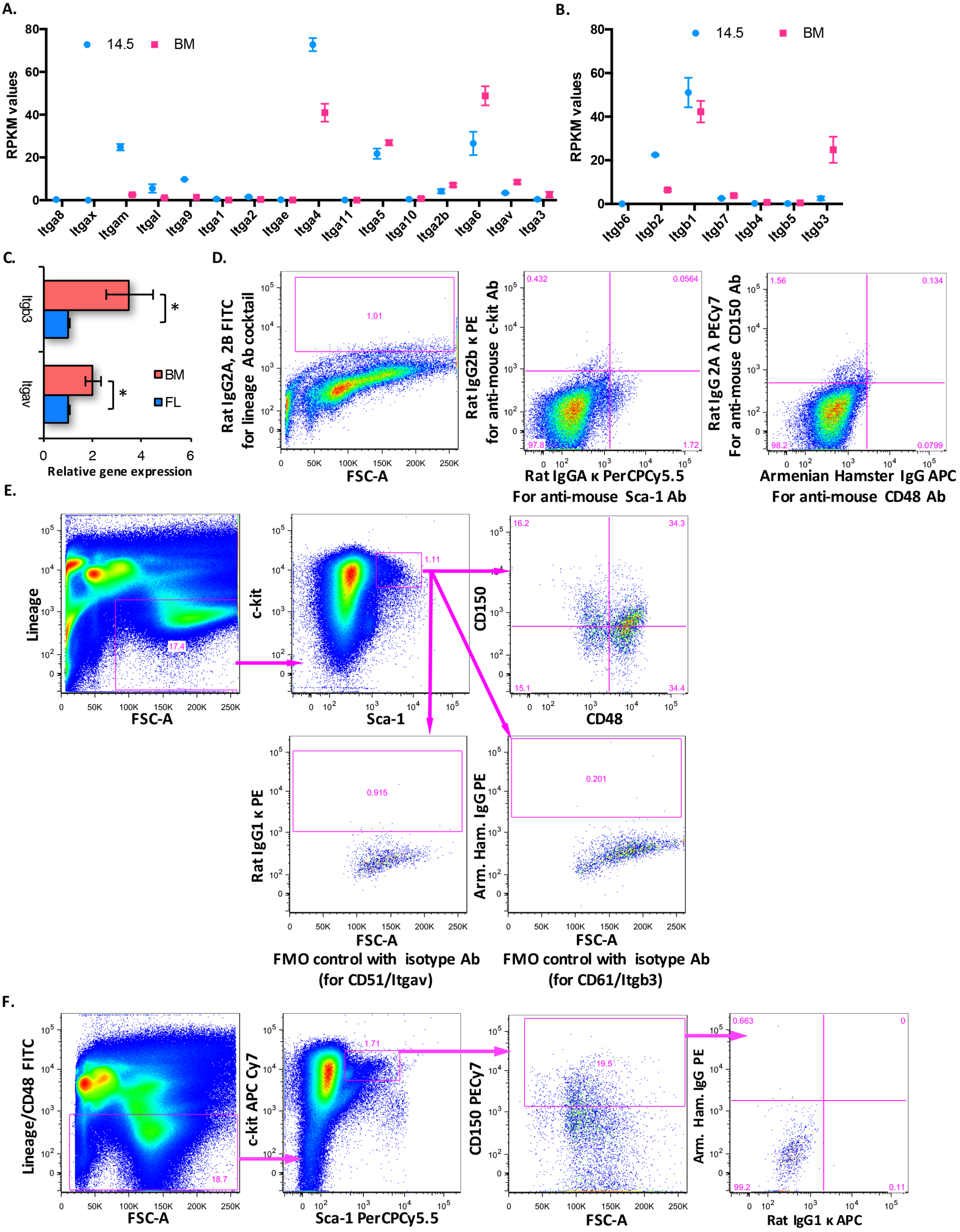

A.
RPKM values
14.5
BM
Itga8
Itgax
Itgam
Itgal
Itga9
Itga1
Itga2
Itgae
Itga4
Itga11
Itga5
Itga10
Itga2b
Itga6
Itgav
Itga3
B.
RPKM values
14.5
BM
Itgb6
Itgb2
Itgb1
Itgb7
Itgb4
Itgb5
Itgb3
C.
Itgb3
Itgav
BM
FL
*
*
Relative gene expression
D.
Rat IgG2A, 2B FITC for lineage Ab cocktail
FSC-A
1.01
Rat IgG2b κ PE for anti-mouse c-kit Ab
Rat IgGA κ PerCPCy5.5 For anti-mouse Sca-1 Ab
0.432
0.0564
97.8
1.72
Rat IgG 2A λ PECy7 For anti-mouse CD150 Ab
Armenian Hamster IgG APC For anti-mouse CD48 Ab
1.56
0.134
98.2
0.0799
E.
Lineage
FSC-A
17.4
c-kit
Sca-1
1.11
CD150
CD48
16.2
34.3
15.1
34.4
Rat IgG1 κ PE
FSC-A
0.915
FMO control with isotype Ab (for CD51/Itgav)
Arm. Ham. IgG PE
FSC-A
0.201
FMO control with isotype Ab (for CD61/Itgb3)
F.
Lineage/CD48 FITC
FSC-A
18.7
c-kit APC Cy7
Sca-1 PerCPCy5.5
1.71
CD150 PECy7
FSC-A
19.5
Arm. Ham. IgG PE
Rat IgG1 κ APC
0.663
0
99.2
0.11

Figure S1: Expression of Itgav and Itgb3 in FL HSCs.

(A,B) Expression of all known α- (A) and β- (B) integrin chains analyzed by RNASeq of primitive HSCs from E14.5 FL and adult BM. $CD150^{+}CD48^{-}$ LSK cells were sorted out from the two stages to perform paired end sequencing, reported in an earlier study from our group. RPKM values for each gene is presented.

(C) Freshly sorted primitive HSCs ($CD150^{+}CD48^{-}$LSK cells) were used to assess the expression of *Itgav* and *Itgb3* by performing quantitative RT-PCR (Unpaired two tailed Student's t-test was performed. n=6, * $p<0.05$).

(D) Isotype antibody controls for the antibodies used for flow cytometry analysis of different stem cell sub-populations in hematopoietic system. Isotype controls used were FITC conjugated Rat IgG2a and 2b for lineage cocktail, PE/APC-Cy7 conjugated Rat IgG2bκ for c-kit, PerCP-Cy5.5 conjugated Rat IgG2a κ for Sca-1, FITC/APC conjugated Armenian hamster IgG for CD48, PE-Cy7 conjugated Rat IgG2a λ for CD150.

(E) Gating strategy and isotype/FMO controls to examine Itgav and Itgb3 expression in various stem cell sub-populations in E14.5 FL cells. Total mononuclear cells (MNCs) wee labeled with FITC conjugated lineage antibody cocktail, APC conjugated c-kit, PerCP-Cy5.5 conjugated Sca-1, APCCy7 conjugated CD48 and PE-Cy7 conjugated CD150 antibodies. Along with these Isotype antibodies for Itgav (CD51) or Itgb3 (CD61) were also used, separately. These were PE conjugated Rat IgG1κ for CD51 and PE conjugated Armenian hamster IgG was used for CD61.

(F) Fluorescence minus one (FMO) control used for co-expression analysis of Itgav and Itgb3 on primitive HSCs derived from E14.5 FL MNCs.

Related to Figure 1

Figure S2

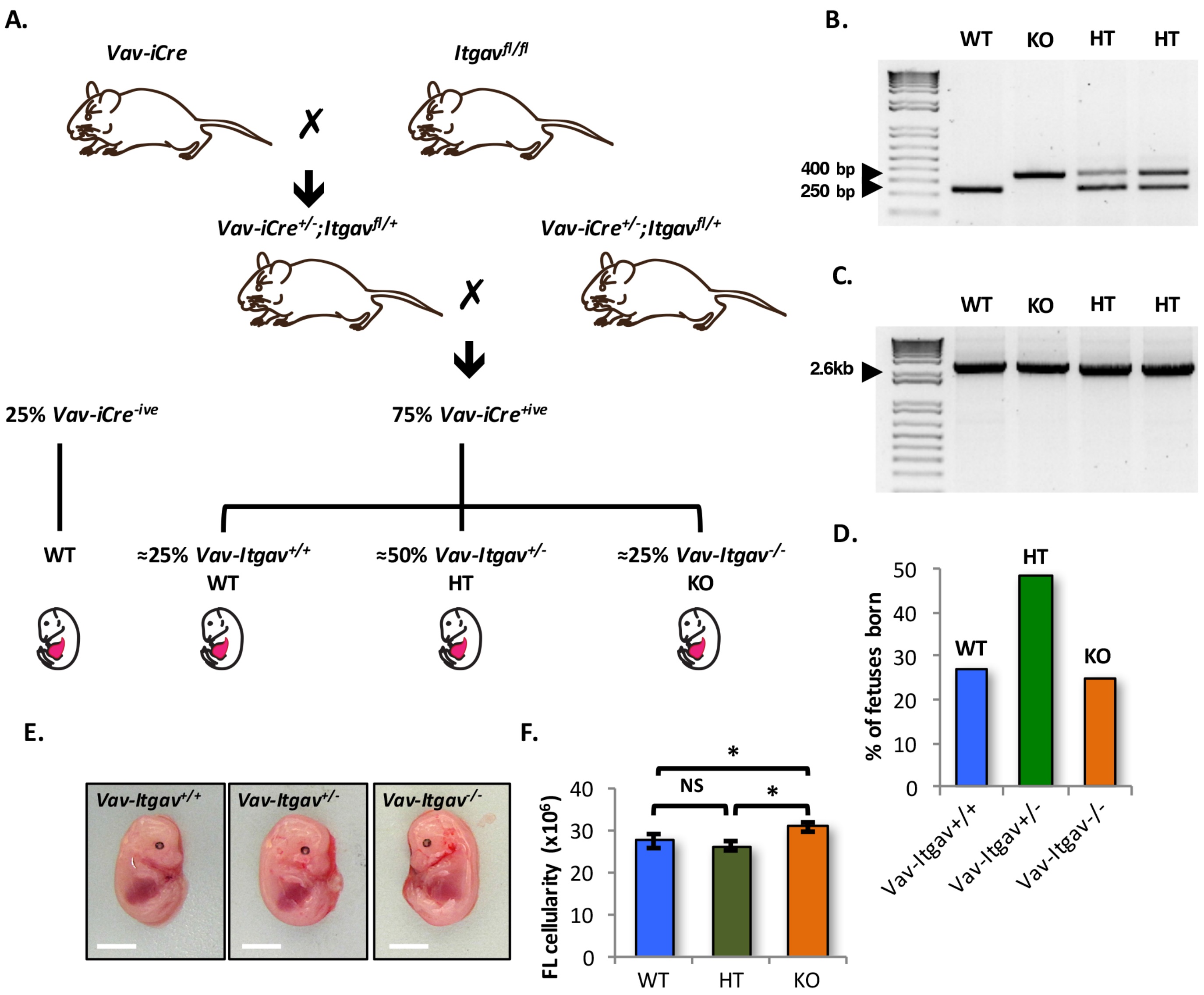

A.
Vav-iCre
Itgavfl/fl
Vav-iCre+/-;Itgavfl/+
Vav-iCre+/-;Itgavfl/+
25% Vav-iCre-ive
75% Vav-iCre+ive
WT
≈25% Vav-Itgav+/+
WT
≈50% Vav-Itgav+/-
HT
≈25% Vav-Itgav-/-
KO
B.
WT
KO
HT
HT
400 bp
250 bp
C.
WT
KO
HT
HT
2.6kb
D.
% of fetuses born
50
40
30
20
10
0
WT
HT
KO
Vav-Itgav+/+
Vav-Itgav+/-
Vav-Itgav-/-
E.
Vav-Itgav+/+
Vav-Itgav+/-
Vav-Itgav-/-
F.
FL cellularity (x10^6)
40
30
20
10
0
NS
*
*
WT
HT
KO

Figure S2: Generation and preliminary characterization of *Vav-Itgav*$^{-/-}$ fetuses

A) *Vav-iCre* and *Itgav*$^{fl/fl}$ mice were crossed to conditionally delete *Itgav* in hematopoietic system. Fetuses at E14.5 from F2 generation were harvested and genotyped to identify *Vav-Itgav*$^{+/+}$ (WT), *Vav-Itgav*$^{+/-}$ (HT), *Vav-Itgav*$^{-/-}$ (cKO) embryos.

B,C) Genotyping PCR was performed to identify *Itgav*$^{+}$/*Itgav*^{fl} alleles as well as *Vav-iCre* construct using specific primers given in Table S1.

D) Comparison between the numbers of *Vav-iCre*$^{+}$ fetuses from each genotype *Vav-Itgav*$^{+/+}$, *Vav-Itgav*$^{+/-}$, *Vav-Itgav*$^{-/-}$. To test the goodness of fit of the observed ratios among genotypes of embryos to expected mendelian ratio, Chi-square test was employed (n=15-29, $\chi^2_{df=2}$=0.049, p=0.98).

E) Gross morphology of the fetuses upon mono- or bi-allelic deletion of *Itgav* in comparison to control littermates. Scale bar=5mm

F) Comparison of the total number of mononuclear cells from fetal liver tissue per embryo. (n=3, N=8-12, t test: * p<0.05)

Related to Figure 2

Figure S3

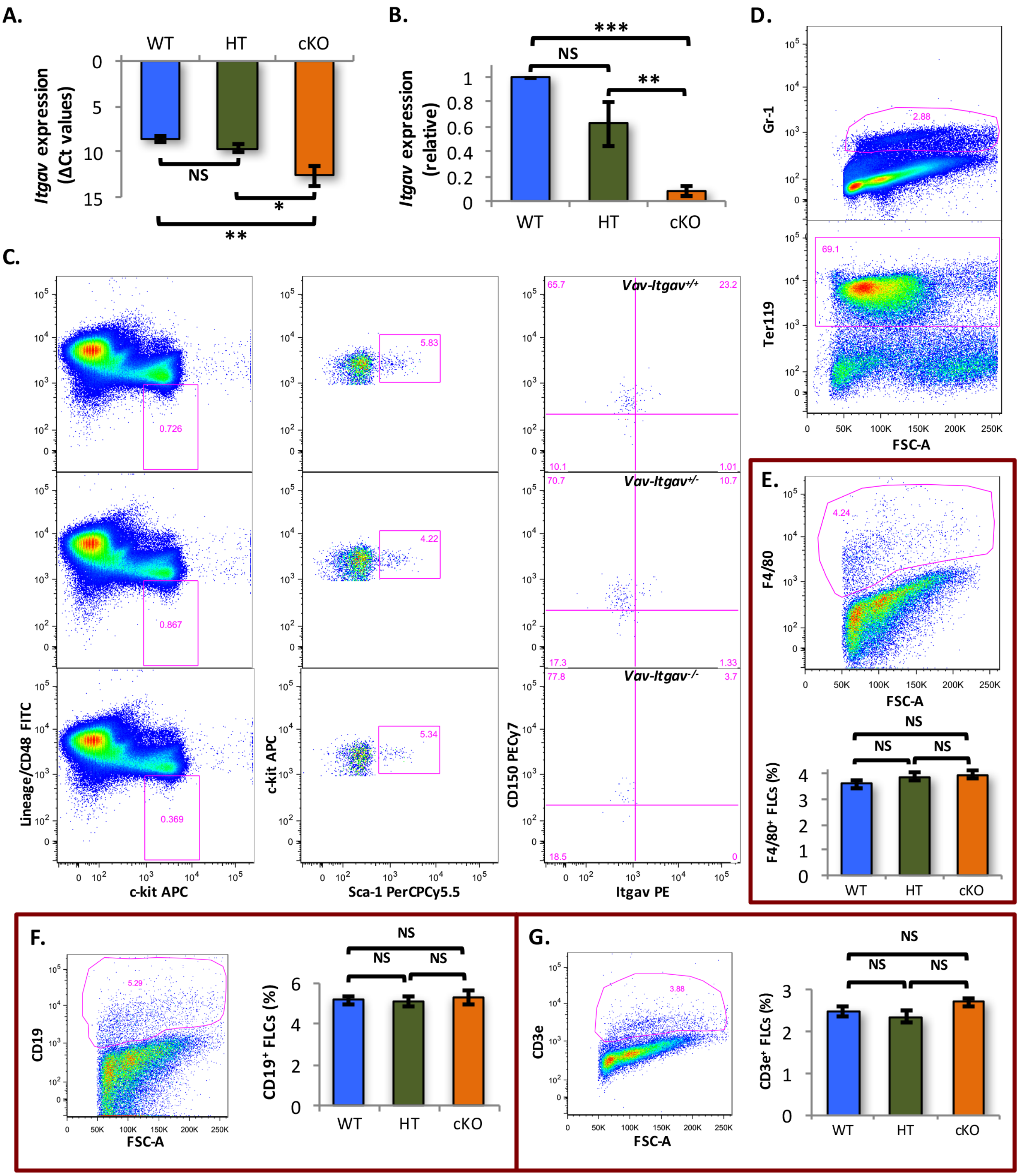

A.
WT
HT
cKO
Itgav expression (ΔCt values)
NS
*
**
B.
***
NS
**
Itgav expression (relative)
C.
Vav-Itgav+/+
Vav-Itgav+/-
Vav-Itgav-/-
Lineage/CD48 FITC
c-kit APC
Sca-1 PerCPCy5.5
CD150 PECy7
Itgav PE
D.
Gr-1
Ter119
FSC-A
E.
F4/80
F4/80+ FLCs (%)
F.
CD19
CD19+ FLCs (%)
G.
CD3e
CD3e+ FLCs (%)

Figure S3: Confirmation of Itgav deletion and lineage analysis of E14.5 *Postn*$^{-/-}$ fetal liver tissues

A,B) Confirmation of *Vav-iCre* mediated *Itgav*-deletion in hematopoietic stem and progenitor cells. LSK cells from FL tissues were sorted from *Vav-Itgav*$^{+/+}$ (WT), *Vav-Itgav*$^{+/-}$ (HT), *Vav-Itgav*$^{-/-}$ (cKO) embryos. Freshly isolated cells were used for quantification of *Itgav* expression using quantitative RT-PCR analysis. Expression levels were shown using ΔCt values (A) and comparisons were made by calculating fold changes following mono- or bi-allelic deletion of *Itgav* (B).

(C) Flowcytometry based assessment of Itgav expression in primitive HSCs from *Vav-Itgav*$^{+/+}$ (WT), *Vav-Itgav*$^{+/-}$ (HT), *Vav-Itgav*$^{-/-}$ (KO) embryos.

D-G) Flow cytometry based analysis performed to compare the frequency of various lineage-committed cells in E14.5 FL tissue from WT, HT and cKO embryos. (D) Gating strategies followed for analyzing Gr-1^{+} and Ter119^{+} populations. (E-G) Flow cytometry based analysis performed to compare the frequency of F4/80+ (E), CD19+ (F) and CD3e+ cells (G) in E14.5 FL tissue from WT, HT and cKO embryos. Gating strategies are shown in upper panels.

(n=8-12, t-test NS $p<0.05$)

Related to Figure 2

Figure S4

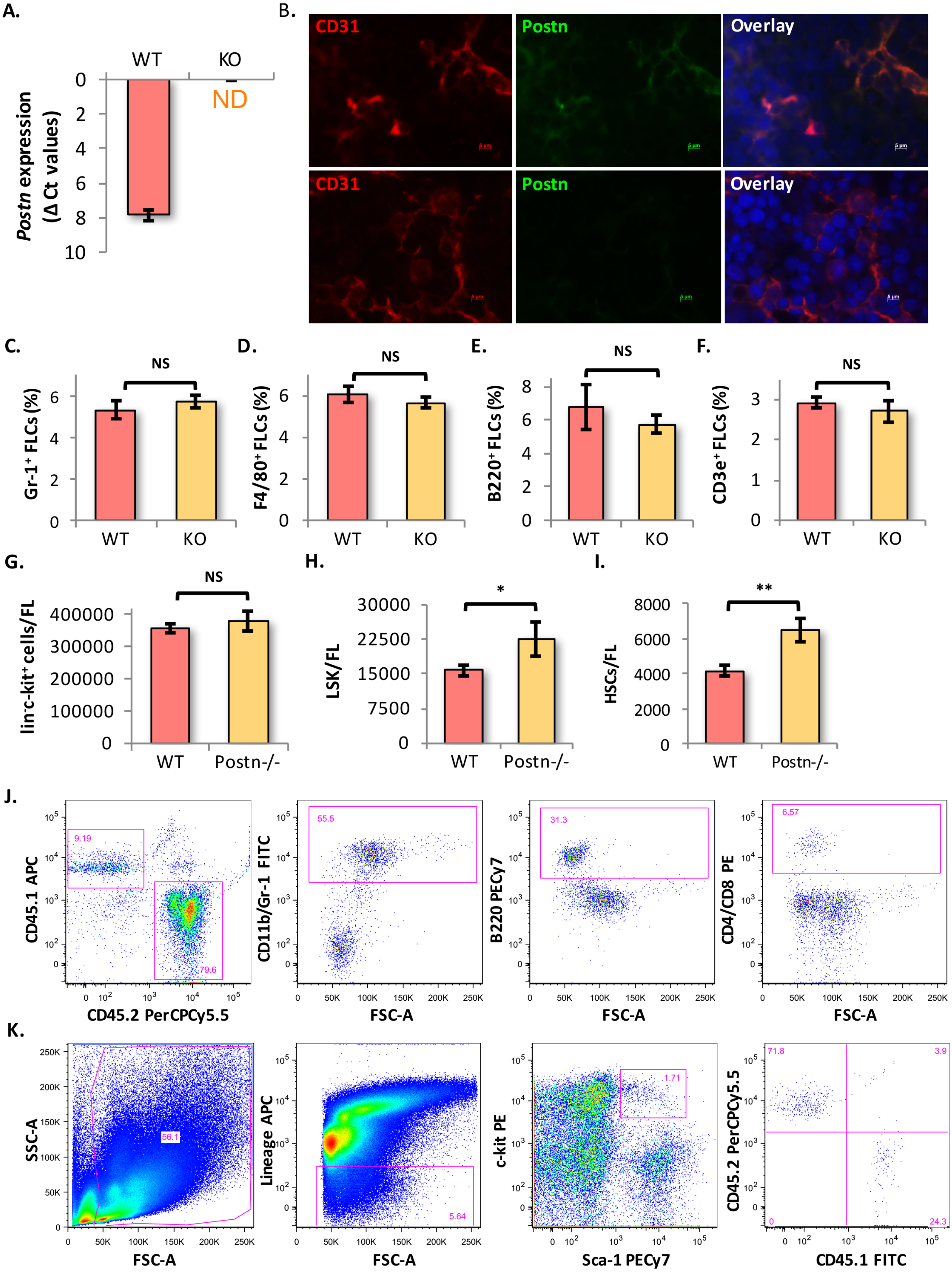

A.
WT
KO
ND
Postn expression (Δ Ct values)
B.
CD31
Postn
Overlay
C.
NS
Gr-1+ FLCs (%)
WT
KO
D.
NS
F4/80+ FLCs (%)
E.
NS
B220+ FLCs (%)
F.
NS
CD3e+ FLCs (%)
G.
NS
lin-c-kit+ cells/FL
WT
Postn-/-
H.
*
LSK/FL
I.
**
HSCs/FL
J.
CD45.1 APC
CD45.2 PerCPCy5.5
9.19
79.6
CD11b/Gr-1 FITC
55.5
FSC-A
B220 PECy7
31.3
CD4/CD8 PE
6.57
K.
SSC-A
56.1
Lineage APC
5.64
c-kit PE
Sca-1 PECy7
1.71
CD45.2 PerCPCy5.5
CD45.1 FITC
71.8
3.9
0
24.3

Figure S4: Lineage analysis of E14.5 *Postn*$^{-/-}$ fetal liver tissues

A) Confirmation of lack of *Postn* expression in hematopoietic niche in the E14.5 fetal liver. Quantitative RT-PCR was performed on lin$^-$CD45$^-$ cells isolated from the FL tissues by MACS.

B) Postn specific antibodies were used to detect its expression in WT FL tissue at E14.5 (upper panel). Vasculature was identified by using anti-CD31 antibodies. Nuclear counterstaining was performed using Hoechst 33342. Lack of expression of Postn in FL tissues from *Postn*$^{-/-}$ embryos was also confirmed (lower panel). Scale bar=5µm

(C-F) Mononuclear cells from E14.5 fetal liver cells were analyzed for the proportion of; C) Gr-1$^+$ (granulocytes), D) F4/80$^+$ (macrophages), E) B220$^+$ cells (B-cells) and F) CD3e$^+$ cells (T-cells). (n=11-14, t-test NS p<0.05)

(G-I) Comparison of the total number of hematopoietic stem and progenitor cells per FL from WT, Postn$^{-/-}$ embryos at E14.5; (G) lin$^-$c-kit$^+$ cells, (H) LSK cells, (I) primitive HSCs.

J) Gating strategy used to quantify donor-derived chimerism. CD45.1$^+$ cells were identified as donor-derived cells, while CD45.2$^+$ cells were recognized as recipients' leukocytes. Multi-lineage chimerism into myeloid (CD11b/Gr-1$^+$ cells), B-cell (B220$^+$) and T-cell lineage (CD4/CD8$^+$) was examined within the donor-derived population in animals transplanted with FL cells or freshly isolated FL HSCs from WT/KO embryos.

K) Gating strategy followed to quantify donor-derived chimerism in BM LSK cell population. LSK cells was gated from within the total BM MNC population and the proportion of donor-derived cells was examined by using antibodies against CD45.1 and CD45.2.

An unpaired two tailed Student's t-test was performed. n=3, N=15-18, t test: * p<0.03, **p≤0.01, NS indicates not significant.

Related to Figure 4

Figure S5

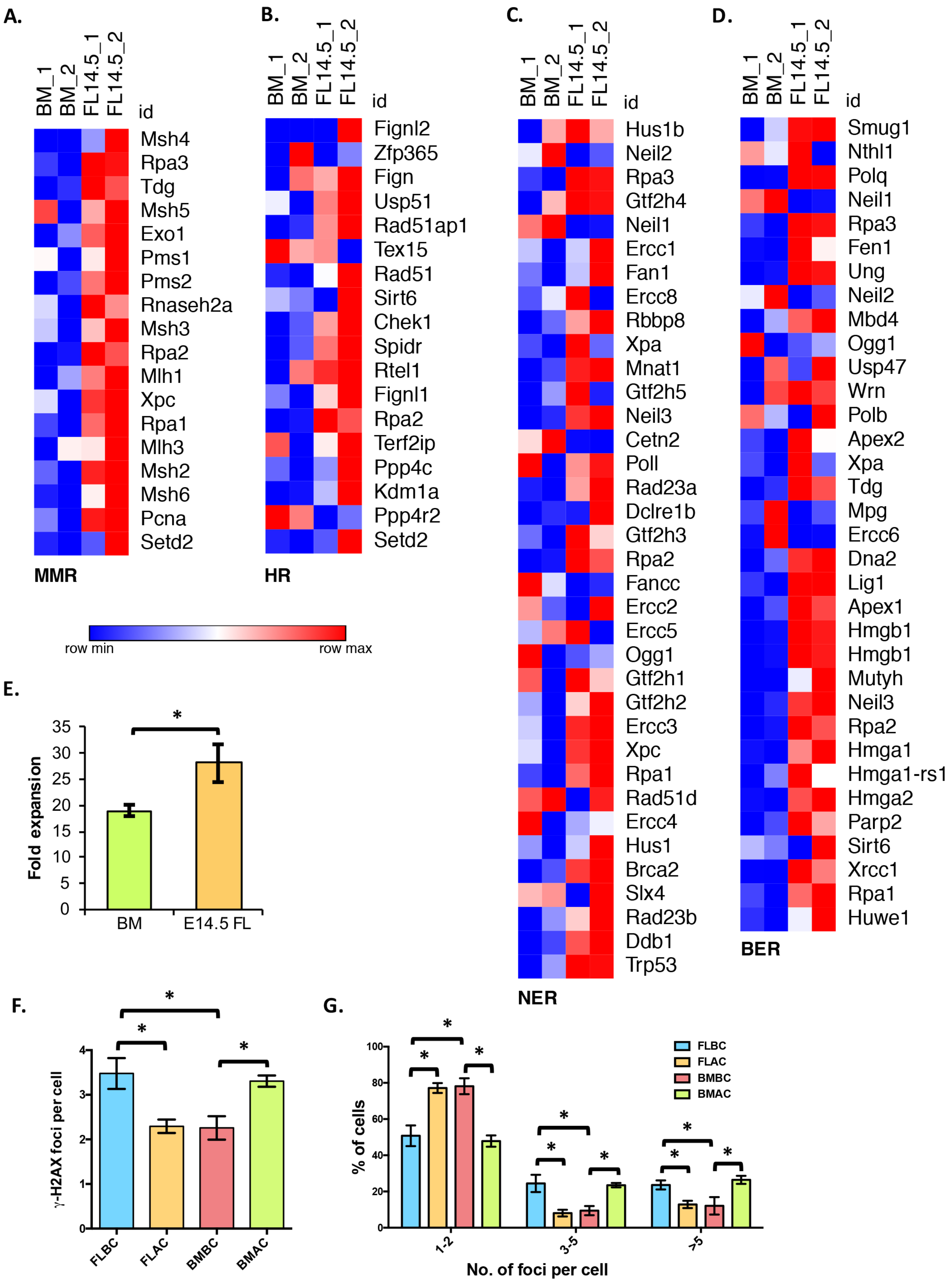

A.
BM_1
BM_2
FL14.5_1
FL14.5_2
id
Msh4
Rpa3
Tdg
Msh5
Exo1
Pms1
Pms2
Rnaseh2a
Msh3
Rpa2
Mlh1
Xpc
Rpa1
Mlh3
Msh2
Msh6
Pcna
Setd2
MMR
B.
Fignl2
Zfp365
Fign
Usp51
Rad51ap1
Tex15
Rad51
Sirt6
Chek1
Spidr
Rtel1
Fignl1
Rpa2
Terf2ip
Ppp4c
Kdm1a
Ppp4r2
Setd2
HR
row min
row max
C.
Hus1b
Neil2
Rpa3
Gtf2h4
Neil1
Ercc1
Fan1
Ercc8
Rbbp8
Xpa
Mnat1
Gtf2h5
Neil3
Cetn2
Poll
Rad23a
Dclre1b
Gtf2h3
Rpa2
Fancc
Ercc2
Ercc5
Ogg1
Gtf2h1
Gtf2h2
Ercc3
Xpc
Rpa1
Rad51d
Ercc4
Hus1
Brca2
Slx4
Rad23b
Ddb1
Trp53
NER
D.
Smug1
Nthl1
Polq
Neil1
Rpa3
Fen1
Ung
Neil2
Mbd4
Ogg1
Usp47
Wrn
Polb
Apex2
Xpa
Tdg
Mpg
Ercc6
Dna2
Lig1
Apex1
Hmgb1
Hmgb1
Mutyh
Neil3
Rpa2
Hmga1
Hmga1-rs1
Hmga2
Parp2
Sirt6
Xrcc1
Rpa1
Huwe1
BER
E.
Fold expansion
35
30
25
20
15
10
5
0
BM
E14.5 FL
*
F.
γ-H2AX foci per cell
4
3
2
1
0
FLBC
FLAC
BMBC
BMAC
*
G.
% of cells
100
80
60
40
20
0
1-2
3-5
>5
No. of foci per cell
FLBC
FLAC
BMBC
BMAC
*

Figure S5: DNA damage response pathway genes are enriched in E14.5 FL derived LT-HSCs

A-D) Heatmap showing relative transcript levels of genes from DNA damage response pathways. RPKM values were used from RNA-Seq analysis of FACS sorted HSCs from E14.5 FL and adult BM HSCs (accession number E-MTAB-4034).

A) mismatch repair (MMR), **B)** homologous recombination (HR), **C)** nucleotide excision repair (NER), and **D)** base excision repair (BER) differentially regulated in FL versus BM derived LT-HSCs identified earlier through KEGG pathway analysis.

E-G) Bone marrow and E14.5 FL derived LSK cells were cultured in serum free medium in the presence of SCF and Tpo for five days. Freshly cells were counted and immunostaining for γ-H2AX was performed on freshly isolated (before culture; BC) and cultured LSK (after culture; AC) cells from E14.5 FL and adult BM.

E) Comparison of the expansion potential of BM and E14.5 FL derived LSKs. The ratio of cells harvested after 5 days of culture with starting population was compared between the two developmental stages.

F) Quantification of γ-H2AX foci per cell in freshly isolated or cultured FL and BM derived LSK cells.

G) Comparison of proportion of cells with different levels of DNA damage based on number of γ-H2AX foci per cell.

n=4, N>173, one-way ANOVA followed by Tukey's multiple comparisons test * $p<0.05$.

Related to Figure 5 and Figure 6

Figure S6

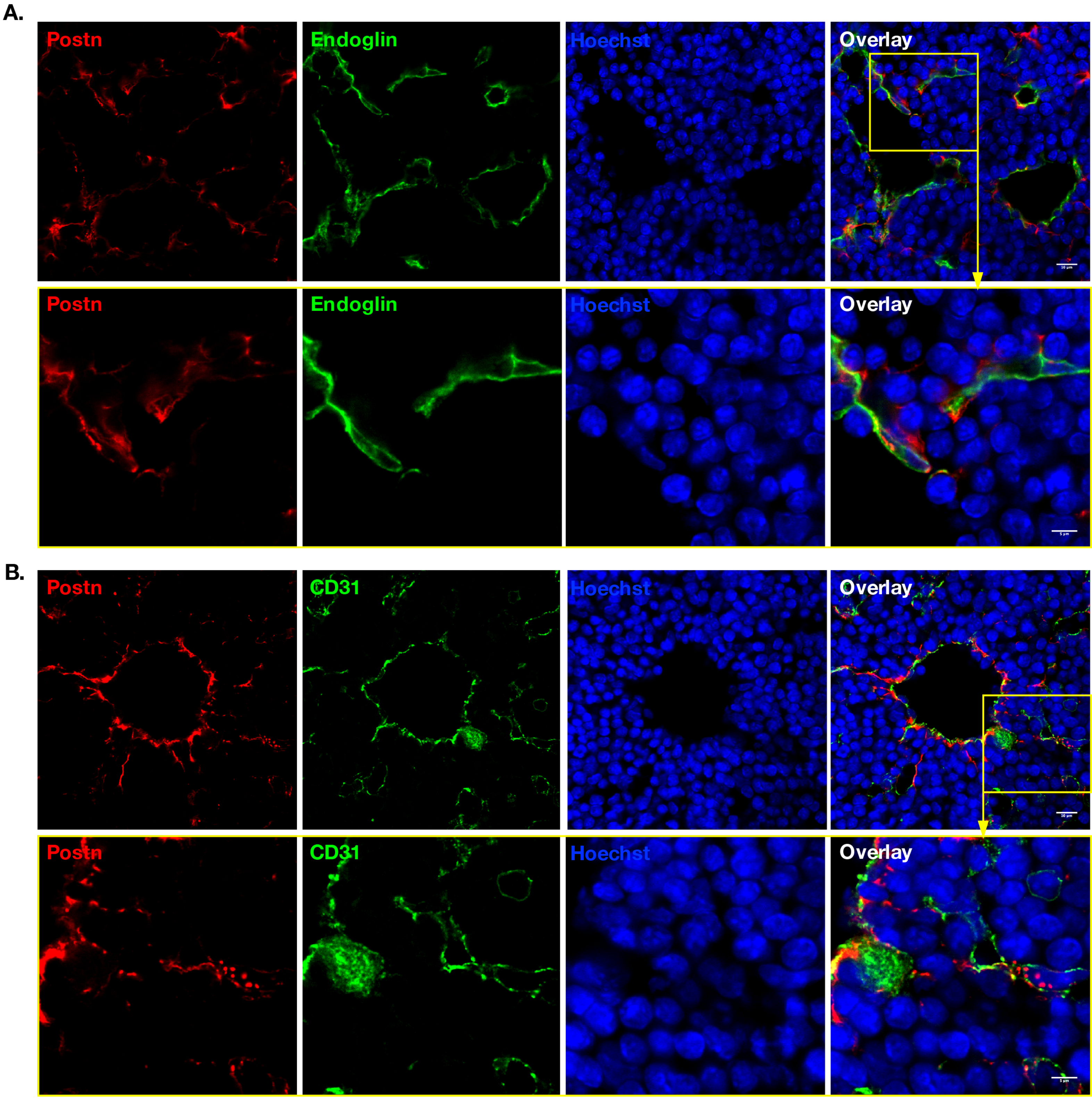
A.
Postn
Endoglin
Hoechst
Overlay
Postn
Endoglin
Hoechst
Overlay
B.
Postn
CD31
Hoechst
Overlay
Postn
CD31
Hoechst
Overlay

Figure S6: Postn is expressed in vascular endothelium of the fetal liver

Immuno-staining performed to identify the cells that express Postn. Vascular endothelial cells were identified by using specific markers; (A) Endoglin and (B) CD31. Immunostaining was performed using Postn specific antibodies along with the endothelial cell specific markers. Lower panels for both A and B show enlarged portion of the sections shown in the upper panel. Counterstaining for nucleus was performed using Hoechst 33342 (n=3-5, scale bar= 10µm for upper panels and 5µm for the lower panels)

Related to Figure 7

Table S1. Primer sets used in this study, related to Figure 5, 6, S1, S2, S3 and S4

S.No.	**Primer Name**	**Sequence 5'-3'**
1	Genotyping Postn-/-_F	GGT GCT TCT GTA AGG CCA TC
	Genotyping Postn-/-_R	GTG AGC CAG GAC CTT GTC ATA
	Genotyping Postn-/-_Int-as	AGC ACT GAC TGC GTT AGC AA
2	Genotyping Itgav-fl/fl_F	GGTGACTCAATCTGTGACCTTCAGC
	Genotyping Itgav-fl/fl mice_R	CACAAATCAAGGATGACCAAACTGAG
3	Genotyping Vav-iCre_F	CCATGGCACCCAAGAAGAAG
	Genotyping Vav-iCre_R	GCTTAGTTTTCCTGCAGCGG
4	Mm-Topbp1_F	AGAGGCTACTGCCCAGAACA
	Mm-Topbp1_R	CGAGGCCGTTTGACTACATT
5	Mm-Brca1_F	GGCTTGACCCCCAAAGAAGT
	Mm-Brca1_R	TGTCCGCTCACACACAAACT
6	Mm-Palb2_F	GGGAAACGAAAATCAGCCCG
	Mm-Palb2_R	AACCACGCCTCTGTTCTGAC
7	Mm-Mre11a_F	CTGGGAGCGGTTTTCTTGTG
	Mm-Mre11a_R	TGGATCTGTGGGGCTCATTT
8	Mm-Blm_F	CTTGGGAGCTGAAAGAGGTG
	Mm-Blm_R	AACGAGGAAGAAGCAGTGGA
9	Mm-Pcna_F	GAGGGTTGGTAGTTGTCGCT
	Mm-Pcna_R	CTCAAACATGGTGGCGGAGT
10	Mm-Rfc4_F	GCCAAAGCACAACTGACCAAG
	Mm-Rfc4_R	CACTGCAACCACTTCGTCCT
11	Mm-Rfc5_F	AGAACGCCTTGAGACGAGTG
	Mm-Rfc5_R	TCAGAGGGCCAAATCGGAAC
12	Mm-Rpa3_F	CGCCAGCATGTTACCACAGTA
	Mm-Rpa3_R	ATTTCCTCGTCAAGTGGCTCC
13	Mm-Ddb1_F	GTGTCTCAAGAGCCCAAAGC
	Mm-Ddb1_R	TCTCTGTGTGGCTGATTTGC
14	Mm-Cetn2_F	TGCAGTGGCTTCTTAGTTGTCC
	Mm-Cetn2_R	ATGCCACAGCAAGCACTCAT
15	Mm-Neil1_F	AAGGGGCTGGTATTTGGTGG
	Mm-Neil1_R	CTCAATGTCAAGCGCAGCTC
16	Mm-Neil3_F	CGGTGGAAAGCCAACAGAGA
	Mm-Neil3_R	ACACATCACACAGCATCCGA
17	Mm-Xrcc1_F	AAAGAGTGGGTGCTGGACTG
	Mm-Xrcc1_R	AGCTTGGGAGCTTCGTCTTC
18	Mm-Tdg_F	CCCCGATCCTGTGCTATTCTC
	Mm-Tdg_R	GTCACGGTTGCCATGTTAGG
19	Mm-Exo1_F	CCTACAGCCACTTAGACCCAA
	Mm-Exo1_R	ACAATCTTAGGCAGACAGCTCC
20	Mm-Setd2_F	CCCGACCCCTGAAGAAGAAG
	Mm-Setd2_R	CCGTCCCTGTTCCTCCAAAT
21	Mm-Ercc1_F	AAGAACTTCGCCCTTCGTGT
	Mm-Ercc1_R	GCTTCCTCTGCACTCCAGG
22	Mm-Rad51_F	TCTGTAAGTGGGAATGGGTG
	Mm-Rad51_R	TGCTGCATGTAAGACTCCTT
23	Mm-Postn_F	TGCTGCCCTGGCTATATGAG
	Mm-Postn_R	GTAGTGGCTCCCACAATGCC
24	Mm-Itgav_F	CCAGCCCATTGAGTTTGATT
	Mm-Itgav_R	TTTGACCTGCATGGAGCATA

Table S2. List of antibodies used in this study, related to Figure 1, 2, 3, 4, 5, 7, S1, S3, S4, S5, S6.

Sr. No.	Antibody	Company	Catalogue No.	Clone	Isotype
1	anti-mouse B220 FITC	BD Biosciences	553088	RA36B2	Rat IgG2a, κ
2	anti-mouse Ter119 FITC	Ebiosciences	11-5921-85	TER119	Rat IgG2b, κ
3	anti-mouse Gr1 FITC	Ebiosciences	11-5931-85	RB6-8C5	Rat IgG2b, κ
4	anti-mouse CD4 FITC	Tonbo	35-0041	GK1.5	Rat IgG2b, κ
5	anti-mouse CD8a FITC	Tonbo	35-0081	53-6.5	Rat IgG2a, κ
6	anti-mouse c-kit APC	Tonbo	20-1172	ACK2	Rat IgG2b, κ
7	anti-mouse c-kit APCCy7	Biolegend	105826	2B8	Rat IgG2b, κ
8	anti-mouse Sca-1 PerCPCy5.5	Biolegend	122524	D7	Rat IgG2a, κ
9	anti-mouse CD150 PECy7	Ebiosciences	25-1502	mShad150	Rat IgG2a, λ
10	anti-mouse CD48 APCCy7	Biolegend	103432	HM48-1	Armenian Hamster IgG
11	anti-mouse CD48 FITC	Ebiosciences	11-0481-85	HM48-1	Armenian Hamster IgG
12	anti-mouse CD51 Biotin	Ebiosciences	13-0512-82	RMV-7	Rat IgG1, κ
13	anti-mouse CD51 PE	LS Bio	Ls-C106287	RMV-7	Rat IgG1, κ
14	anti-mouse CD61 PE	Ebiosciences	12-0611-83	2C9.G3	Armenian Hamster IgG
15	anti-mouse CD45.1 APC	BD Biosciences	558701	A20	Mouse IgG2a, κ
16	anti-mouse CD45.2 PerCPCy5.5	BD Biosciences	552950	104	Mouse IgG2a, κ
17	anti-mouse F4/80 APCCy7	Biolegend	123117	BM8	Rat IgG2a, κ
18	anti-mouse CD3e	BD Biosciences	553064	145-2C11	Armenian Hamster IgG1 κ
19	anti-mouse Postn	R&D	AF2955	Polyclonal	Goat IgG
20	anti-mouse CD31	R&D	AF3628	Polyclonal	Goat IgG
21	anti-mouse endoglin	eBioscience	13-1051-82	MJ7/18	Rat IgG2a, κ
22	anti-mouse laminin	abcam	ab11575	Polyclonal	Rabbit IgG
23	anti-mouse γ-H2AX AF647	BD	560447	N1-431	Mouse BALB/c IgG1, κ
24	anti-mouse CD11b FITC	Ebiosciences	11-0112-82	M1/70	Rat IgG2b, κ
25	anti-mouse B220 PECy7	Ebiosciences	25-0452-82	RA3-6B2	Rat IgG2a, κ
26	anti-mouse CD4 PE	Tonbo	50-0041-U100	GK1.5	Rat IgG2b, κ
27	anti-mouse CD8 PE	eBioscience	12-0081-82	53-6.7	Rat IgG2a, κ
28	anti-mouse c-kit PE	eBioscience	12-1172-82	ACK2	Rat IgG2b, κ
29	anti-mouse Sca-1 PECy7	Ebiosciences	25-5981-82	D7	Rat IgG2a, κ
30	anti-mouse CD48 APC	Ebiosciences	17-0481-82	HM48-1	Armenian Hamster IgG
31	APC-Lineage antibody cocktail	BD Biosciences	558074	145-2C11	Armenian Hamster IgG1, κ
				M1/70	Rat IgG2b, κ
				RA3-6B2	Rat IgG2a, κ
				TER-119	Rat IgG2b, κ
				RB6-8C5	Rat IgG2b, κ
32	Rat IgG2a, κ FITC	BD Biosciences	553929	R35-95	
33	Rat IgG2a, κ PerCPCy5.5	BD Biosciences	550765	R35-95	
34	Rat IgG2a, κ APCCy7	BD Biosciences	552770	R35-95	
35	Rat IgG2a, λ PECy7	BD Biosciences	560721	B39-4	
36	Rat IgG2b, κ FITC	BD Biosciences	553988	A95-1	
37	Rat IgG2b, κ APC	BD Biosciences	553991	A95-1	
38	Rat IgG2b, κ PE	BD Biosciences	553989	A95-1	

39	Rat IgG2b, κ APCCy7	BD Biosciences	552773	A95-1	
40	Mouse IgG2a, κ APC	BD Biosciences	551414	G155-178	
41	Mouse IgG2a, κ PerCPCy5.5	BD Biosciences	552577	G155-178	
42	Mouse IgG2a, κ PECy7	BD Biosciences	552868	G155-178	
43	Mouse IgG2a, κ PE	BD Biosciences	553458	G155-178	
44	Armenian Hamster IgG APCCy7	Biolegend	400927	HTK888	
45	Armenian Hamster IgG FITC	Biolegend	400905	HTK888	
46	Armenian Hamster IgG PE	Biolegend	400907	HTK888	
47	Armenian Hamster IgG APC	Biolegend	400911	HTK888	
48	Donkey Anti-Goat IgG AF647	Jackson ImmunoResearch	705-607-003	Polyclonal	
49	Donkey Anti-Goat IgG AF488	Jackson ImmunoResearch	705-547-003	Polyclonal	
50	Goat Anti-Rabbit IgG AF 647	Jackson ImmunoResearch	111-607-003	Polyclonal	
51	Goat Anti-Rat IgG AF 647	Life Technologies, Invitrogen	A21247	Polyclonal	

Supplementary Videos

Video S1. Z-stack series and maximum projection of confocal images showing Postn expression in endoglin expressing vascular endothelial cells. Postn in red, endoglin in green and Hoechst 33342 in blue. Scale bar 10µm. Related to figure 6

Video S2. Z-stack series and maximum projection of zoomed-in confocal images showing Postn expression in endoglin expressing vascular endothelial cells. Postn in red, endoglin in green and Hoechst 33342 in blue. Scale bar 10µm. Related to figure 6

Video S3. Z-stack series and maximum projection of confocal images showing Postn expression in CD31 expressing vascular endothelial cells. Postn in red, CD31 in green and Hoechst 33342 in blue. Scale bar 10µm. Related to figure 6

Video S4. Z-stack series and maximum projection of zoomed-in confocal images showing Postn expression in CD31 expressing vascular endothelial cells. Postn in red, CD31 in green and Hoechst 33342 in blue. Scale bar 10µm. Related to figure 6

Video S5. Z-stack series and maximum projection of confocal images showing Postn co-localization with ECM protein laminin around CD31 expressing vascular endothelial cells. Postn in red, CD31 in green, laminin in blue and Hoechst 33342 in white. Scale bar 10µm. Related to figure 6

Video S6. Z-stack series and maximum projection of zoomed-in confocal images showing Postn co-localization with ECM protein laminin around CD31 expressing vascular endothelial cells. Postn in red, CD31 in green, laminin in blue and Hoechst 33342 in white. Scale bar 10µm. Related to figure 6

Supplemental experimental procedures

Animals: Six to eight weeks old FVB/NJ, C57BL/6J-CD45.2 (Centre d'Elevage R. Janvier, Le Genest-St Isle, *France)*, B6.SJL-PTPRCA-CD45.1 (Charles River Laboratories, Raleigh, NC), *Postn*$^{-/-}$ (generated in FVB/NJ background) [1], *Vav-iCre* [2] (from Thomas Graf, Centre for Genomic Regulation, Barcelona), *Itgav*$^{fl/fl}$ [3], *Rag2*$^{-/-}$*γC*$^{-/-}$ (from Prof. Chantal Mathieu, Clinical and Experimental Endocrinology, UZ Leuven, Leuven, Belgium) mice were used. The animals were bred at the animal facilities of KU Leuven, Rajiv Gandhi Centre for Biotechnology (RGCB) and IISER Thiruvananthapuram. During the experiments, mice were maintained in isolator cages, fed with autoclaved acidified water, and irradiated food ad libitum. At IISER TVM and RGCB, the animals were maintained as per guidelines provided by the Committee for the Purpose of Control and Supervision of Experiments on Animals (CPCSEA), Ministry of Environment and Forests, Government of India. All animal experiments were approved by the Institutional Animal Ethics Committees for the respective animal facilities.

The fetuses were isolated at E14.5 and genomic DNA from limb bud tissue was used for genotyping. Age of the fetuses was determined by considering the vaginal plug detection at E0.5. Primers used for genotyping of the *Vav-iCre; Itgav*$^{fl/fl}$ and *Postn*$^{-/-}$ lines are listed in Tabe S1.

Isolation of LT-HSC: LT-HSCs were sorted using specific antibodies to identify lin$^-$c-kit$^+$Sca-1$^+$CD150$^+$CD48$^-$ cells. Fetal liver (FL) derived HSCs have been shown to express CD11b [4], therefore we removed anti-CD11b antibody from the lineage cocktail for sorting HSCs from FL tissues. The tissues were obtained from timed pregnant C57BL/6J female mice. Embryos were dissected at embryonic day (E) 14.5 (14 days after vaginal plug was observed). Mononuclear cell suspension was prepared by disrupting the tissue and repeated pipetting. The cells were washed with phosphate-buffered saline (PBS; Gibco Invitrogen, CA) containing 0.1% bovine serum albumin (BSA; Sigma). Ter-119 depletion was performed to deplete erythrocytes and erythroid progenitors by magnetic separation using MACS columns (Miltenyi Biotec, Germany). Ter-119 depleted cells were then stained with Alexa Fluor 488 conjugated Anti-lineage antibody cocktail (containing CD4, CD5, CD8a, CD45R, Ter-119, GR-1), PE conjugated anti-c-kit, APC conjugated anti-Sca-1, PE-Cy7 conjugated anti-CD150 and Alexa Fluor 488 conjugated anti-CD48. Cells were incubated on ice for 30 minutes. After staining the FL cells, HSCs were sorted by fluorescence-activated cell sorting (FACS), using a FACS ARIAIII (Becton Dickinson). Total adult bone marrow (ABM) cells, to be transplanted together with sorted HSCs for competitive repopulation assays, were flushed from femurs and tibiae of female C57BL/6J mice, washed twice with phosphate-buffered saline (PBS; Gibco Invitrogen, CA) containing 0.1% bovine serum albumin (BSA; Sigma). List of all antibodies used for flow cytometry based sorting and analysis is given in Table S2.

Cell Cycle analysis: Cell cycle analysis was performed by Hoechst 33342 (Ho) and Pyronin Y (PY) staining on cells labeled for HSPC markers as described before [5]. Fetal liver derived cells were first stained for HSPC markers (Lineage, Sca-1, c-kit) followed by Hoechst 33342 (Ho) alone or in combination with Pyronin Y (PY) staining as described before [5]. Cells were acquired on FACS Aria III (BD Biosciences) and analysed using FlowJo software (TreeStar, Ashland, OR).

Quantitative RT-PCR: Total RNA was prepared using the RNA Isolation Kit (Qiagen, Hilden, Germany), according to the manufacturer's protocol. DNase treatment of RNA was performed using Turbo DNase kit (Ambion, Austin, TX, USA). The purity and the concentration of RNA were assessed using a micro-volume spectrophotometer (Colibri, Berthold Technologies GmBH & Co. KG Germany). 100ng-1µg of RNA from each sample was used to synthesize cDNA using Superscript III First-Strand Synthesis System (Invitrogen, Carlsbad, CA) according to the manufacturer's protocol. qRT-PCR was carried out using Taqman SYBR green universal mix PCR reaction buffer (Applied Biosystems, Foster City, CA). The PCR reactions were carried out in a CFX96 detection system (Thermal-Cycler C1000, Biorad, Hercules, CA, USA). The list of primers used is given in Table S1.

Long-term repopulation assays: A single dose of 3.5 Gy (sub-lethal dose) or 10Gy (sub-lethal dose) radiation on a RAD SOURCE RS-2000 biological Irradiator (Rad Source Technologies, Alpharetta , GA) was given to the animals, a day before the test cells were transplanted. To compare the frequency of HSCs in E14.5 *Vav-Itgav*$^{+/+}$, *Vav-Itgav*$^{+/-}$ *and Vav-Itgav*$^{-/-}$ FL tissue, 10,000 FL mononuclear cells (CD45.2) along with 90,000 whole BM derived cells (CD45.1) were transplanted into lethally irradiated CD45.1 mice. For testing the function of HSC compartment, freshly sorted 100 HSCs from E14.5 *Vav-Itgav*$^{+/+}$, *Vav-Itgav*$^{+/-}$ *and Vav-Itgav*$^{-/-}$ FL tissues, along with 100,000 whole BM derived cells (CD45.1) were transplanted into lethally irradiated CD45.1 mice. For assessing the effect of Postn deletion on HSC frequency, 50,000 FL mononuclear cells derived from *Postn*$^{+/+}$ and *Postn*$^{-/-}$ embryos (CD45.1 FVB background) were transplanted into sub-lethally (3.5Gy) irradiated *Rag2*$^{-/-}$*γC*$^{-/-}$ mice (CD45.2 background). For testing the function of HSC compartment, freshly sorted 100 HSCs from *Postn*$^{+/+}$ and *Postn*$^{-/-}$ E14.5 FL tissues were transplanted into sub-lethally (3.5Gy) irradiated *Rag2*$^{-/-}$*γC*$^{-/-}$ mice. Peripheral blood chimerism analysis was performed every 4 weeks. After 12 weeks, half of the primary recipients were sacrificed; BM harvested, and 100,000 (for *Vav-Itgav*$^{-/-}$) or $1x10^6$ (for *Postn*$^{-/-}$) cells grafted in lethally or sub-lethally irradiated secondary recipients, respectively. After 3 months, chimerism in secondary recipients was evaluated. The second half of the primary recipients was followed for PB and BM chimerism for upto 24 weeks of transplantation

For chimerism analysis, the percentage of donor derived ($CD45.1^+$ or $CD45.2^+$) cells within the total leukocyte population ($CD45.1^+$ + $CD45.2^+$) was calculated. Multilineage-engraftment data was presented as the percentage of cells from myeloid, B- and T-cell lineage within the donor-derived population. For LSK chimerism in the BM, the proportion of donor-derived cells within the total LSK cell population was calculated and compared between the WT and KO groups.

Bioinformatics analysis: RNASeq analysis of the HSCs across developmental stages was previously published by us before [6]. The differential expression analysis of genes was performed using the R package DESeq2 [7] and differentially expressed genes were identified by the following thresholds: false discovery rate (FDR) < 0.05 and |log fold change| > 1.0. The gene-set enrichment test was then carried out to detect significantly enriched gene sets (also called pathways) using the R package GAGE v2.14.4 [8]. KEGG metabolic pathway maps were constructed to provide an overview of gene expression profiling and molecular interactions within a single pathway by using the R package Pathview [9]. The data can be accessed through ArrayExpress database (www.ebi.ac.uk/arrayexpress) under accession number E-MTAB-4034. This data was analyzed to examine the differential expression of all known genes for various α- and β-integrin chains and genes for pathways involved in DNA damage repair, namely mismatch repair, homologous recombination, nucleotide excision repair, and base excision repair. The lists of integrins and DNA repair pathway genes were extracted from MGI database and respective genes expression from RNA-seq was represented as heat-maps that were generated using Morpheus, https://software.broadinstitute.org/morpheus.

In vitro adhesion assays: For cell adhesion assays, the cells were first labeled with PKH26 membrane dye [10] according to the manufacturer's instructions (Sigma, St Louis, MO, USA). Freshly sorted or cultured LSK cells were harvested and washed with PBS to remove any protein content. The cells were re-suspended at $1x10^7$ per ml of Diluent C. The cell suspension was mixed with an equal volume of 4 mM PKH26 dye (in Diluent C) for 5min at room temperature. An equal volume of fetal bovine serum (FBS) was added to stop the reaction and the cells were washed with medium containing 10% FBS. $5x10^4$ ST2 cells were plated per well in 24 well plates. Ten thousand PKH-26 labeled LSK were added per well and incubated for 3h at 37°C and 5% CO_2. Non-adhered cells were removed and adhered cells were harvested along with the feeder layer. Flowcytometry was used to quantify the labeled cells to compare cell attachment [11]. Results are represented as percentage of cells adhered.

In vivo homing assay: A protocol published earlier [12] originally adapted from previously published reports [13,14] was used to examine the homing potential of BM derived HSPCs. One day prior to transplantation, 6-8 week old B6.SJL-PTPRCA-CD45.1 mice underwent total body lethal

irradiation (10Gy). Two million E14.5 FL cells from *Vav-Itgav*$^{+/+}$, *Vav-Itgav*$^{+/-}$ or *Vav-Itgav*$^{-/-}$ mice were injected intravenously into the irradiated mice. Prior to transplantation, the frequency of CFCs in the transplanted cells were quantified. 16 hours after injection, BM from transplanted mice was harvested and donor derived HSPCs homed in the recipient BM were quantified by CFU-C assay. For each experiment, a set of 6 mice irradiated but not transplanted was used to quantify left over CFCs in the BM of the irradiated mice. For CFC assays, $1x10^5$ cells were plated in 3 ml methylcellulose using MethoCult GF M3434 medium (Stem Cell Technologies). Each sample was cultured in triplicates at 37°C with 5% CO_2 in air. CFCs were scored after 9 to 12 days by light microscope. The proportion of total CFCs transplanted that homed in the total recipient BM represents homing.

Neutral comet assay: The DNA double strand break repair was assayed by neutral single-cell agarose gel electrophoresis, using a method earlier described by Olive et al. [15] and refined by Wojewódzka et al. [16]. The method was used to perform comet assay to assess DNA damage in freshly sorted and cultured hematopoietic stem cells from E14.5 FL and adult BM. Briefly, the test cells (freshly sorted or cultured) were mixed with low-gelling-temperature agarose (Sigma; type VII), and layered onto agarose-coated glass slides. Slides were maintained in the dark at 4°C to gel and for all the steps thereafter. Slides were submerged in lysis buffer (2.5 M NaCl, 0.1 M EDTA, 10 mM Trizma base, 1% Triton X-100, 10% DMSO) for 1.5 h, washed with Tris buffer, and incubated for 45 min in alkaline electrophoresis buffer (300 mM NaOH, 1 mM EDTA at pH 10) or in neutral electrophoresis buffer (300 mM sodium acetate, 100 mM Tris-HCl, 1% DMSO at pH 8.3). After electrophoresis (~40 min, 25 V), air-dried and neutralised slides were stained with 30 µl of ethidium bromide solution (20 µg/ml). Average Comet Tail Moment was scored for 100 cells/slide by using Comet Imager 1.2.10 software (MetaSystems Incorporation, Altlussheim, Germany).

Immunocytochemistry: Teflon-printed 10-well glass slides (Matsunami Glass Industry, Osaka, Japan) were pre-coated with Corning Cell-Tak™ adhesive and incubated for 2 hrs in a humidity chamber. Cell-Tak was removed and 500 of the BM derived LSK cells in 50µl PBS were added. The cells were incubated for 30 minutes at room temperature to adhere to the surface. The supernatant was carefully removed and 4% PFA was added for fixation at room temperature for 10 minutes. PFA was carefully removed and the cells were washed twice with PBS. AF647 conjugated anti-γH2AX antibody (1:100; clone N1-431; BD Pharmingen) in blocking buffer (3% BSA, 3% Goat Serum and 0.3% Triton X-100 in PBS) was added and kept in humidity chamber for overnight incubation at 4°C. The cells were washed twice using PBS and nuclei was counterstained with Hoechst 33342 (10µg/ml) for 2 mins and washed with PBS. Prolong Gold

antifade reagent (Invitrogen) was to mount the cells. The samples were air dried for 12 hrs in dark and visualised under Leica SP5 Confocal Upright Microscope (Leica Microsystems, Heidelberg, GmBH). Scoring of total γH2AX^{+} foci and the number of foci per nucleus was performed using Leica Application Suite X software.

Immunohistochemistry: E14.5 fetuses were harvested from pregnant dams, washed in PBS and fixed with 4% paraformaldehyde (PFA), on ice, for 4 hours. Paraffin blocks were made of the fixed fetuses by following gradient steps for dehydration, clearing and infiltration and finally embedding in fresh Paraplast (Sigma). Microtome (Leica RM2265) was used to cut 10μm longitudinal sections of E14.5 embryo for immunohistochemical assays on FFPE sections. Alternatively, FL tissues were dissected out from the fetuses and subjected to a 30 minute fixation with 2% PFA, on ice. Fixed tissues were subjected to overnight 30% sucrose gradient for cryo-protection prior to cryo-block preparation using Polyfreeze (Sigma). Cryotome (Thermo Scientific HM525 NX) was used to obtain 50μm thick sections of E14.5 FL for immunohistochemical assay on cryo-sections.

FFPE sections on frosted slides (VWR) or floating cryo-sections were immune-labeled using antigen specific primary antibodies; anti-Postn antibody (R&D systems, Minneapolis, MN), AF647 conjugated anti-mouse CD31 antibody (BioLegend), biotin conjugated anti-mouse Endoglin antibody (Invitrogen) and fluorescently labeled secondary antibodies (Jackson ImmunoResearch Laboratories Inc.). Hoechst 33342 (Sigma Aldrich) was used to counterstain the nuclei. Sections were mounted using Prolong Gold antifade mounting medium (Invitrogen). Fluorescence imaging was performed using Leica upright confocal microscope (Leica TCS SP5 II). Images were captured using 63X oil immersion objective (HCX PL APO CS 63.0x1.40 Oil). For imaging whole embryo section, tile scanning followed by auto-stitching was performed using LasAF software. Z-stacks were taken with 0.35μm step size and LasX software was used for analyzing the obtained images.

Statistical analysis: All data are represented as mean ± s.e.m. Normal distribution of data was tested using the Shapiro-Wilk test. The equality of group variance was tested using Brown-Forsythe test. Comparisons between samples from two groups with normally distributed data with equal variance were made using the unpaired two-tailed Student's *t-test*. Mann Whitney test was used for comparing two groups where data were non-normally distributed. For multiple comparisons of the normally distributed data with equal variance, one-way ANOVA was performed followed by Tukey Kramer post hoc test. Non-normally distributed data was analyzed by Friedman test. Chi-square test was employed for testing the goodness of fit of the observed ratios among genotypes of embryos to expected Mendelian ratio. Statistical analyses were

performed with Microsoft Excel or GraphPad Prism 6. For all analyses, p-values ≤0.05 were accepted as statistically significant.

Supplemental references

1. Malanchi I, Santamaria-Martinez A, Susanto E, et al. Interactions between cancer stem cells and their niche govern metastatic colonization. *Nature*. 2012;481(7379):85-89.

2. de Boer J, Williams A, Skavdis G, et al. Transgenic mice with hematopoietic and lymphoid specific expression of Cre. *Eur J Immunol*. 2003;33(2):314-325.

3. Lacy-Hulbert A, Smith AM, Tissire H, et al. Ulcerative colitis and autoimmunity induced by loss of myeloid alphav integrins. *Proc Natl Acad Sci U S A*. 2007;104(40):15823-15828.

4. Morrison SJ, Hemmati HD, Wandycz AM, Weissman IL. The purification and characterization of fetal liver hematopoietic stem cells. *Proc Natl Acad Sci U S A*. 1995;92(22):10302-10306.

5. Arai F, Hirao A, Ohmura M, et al. Tie2/angiopoietin-1 signaling regulates hematopoietic stem cell quiescence in the bone marrow niche. *Cell*. 2004;118(2):149-161.

6. Manesia JK, Xu ZF, Broekaert D, et al. Highly proliferative primitive fetal liver hematopoietic stem cells are fueled by oxidative metabolic pathways. *Stem Cell Research*. 2015;15(3):715-721.

7. Love MI, Huber W, Anders S. Moderated estimation of fold change and dispersion for RNA-seq data with DESeq2. *Genome Biol*. 2014;15(12):550.

8. Luo W, Friedman MS, Shedden K, Hankenson KD, Woolf PJ. GAGE: generally applicable gene set enrichment for pathway analysis. *BMC Bioinformatics*. 2009;10:161.

9. Luo W, Brouwer C. Pathview: an R/Bioconductor package for pathway-based data integration and visualization. *Bioinformatics*. 2013;29(14):1830-1831.

10. Hendrikx PJ, Martens CM, Hagenbeek A, Keij JF, Visser JW. Homing of fluorescently labeled murine hematopoietic stem cells. *Experimental hematology*. 1996;24(2):129-140.

11. Simmons PJ, Masinovsky B, Longenecker BM, Berenson R, Torok-Storb B, Gallatin WM. Vascular cell adhesion molecule-1 expressed by bone marrow stromal cells mediates the binding of hematopoietic progenitor cells. *Blood*. 1992;80(2):388-395.

12. Khurana S, Buckley S, Schouteden S, et al. A novel role of BMP4 in adult hematopoietic stem and progenitor cell homing via Smad independent regulation of integrin-alpha4 expression. *Blood*. 2013;121(5):781-790.

13. Szilvassy SJ, Meyerrose TE, Ragland PL, Grimes B. Differential homing and engraftment properties of hematopoietic progenitor cells from murine bone marrow, mobilized peripheral blood, and fetal liver. *Blood*. 2001;98(7):2108-2115.

14. Frenette PS, Subbarao S, Mazo IB, von Andrian UH, Wagner DD. Endothelial selectins and vascular cell adhesion molecule-1 promote hematopoietic progenitor homing to bone marrow.

Proceedings of the National Academy of Sciences of the United States of America. 1998;95(24):14423-14428.

15. Olive PL, Banath JP, Durand RE. Heterogeneity in Radiation-Induced DNA Damage and Repair in Tumor and Normal-Cells Measured Using the Comet Assay. *Radiation Research*. 1990;122(1):86-94.

16. Wojewodzka M, Buraczewska I, Kruszewski M. A modified neutral comet assay: elimination of lysis at high temperature and validation of the assay with anti-single-stranded DNA antibody. *Mutation Research-Genetic Toxicology and Environmental Mutagenesis*. 2002;518(1):9-20.
