# Supplementary material for: The Periostin/Integrin-αv Axis Regulates the Size of Hematopoietic Stem Cell Pool in the Fetal Liver
Source: Stem Cell Reports. 2020 Jul 30;15(2):340–57. doi: 10.1016/j.stemcr.2020.06.022 (PMC7419718; doi:10.1016/j.stemcr.2020.06.022)
Supplement: Document S2. Article plus Supplemental Information [file mmc8.pdf]

Stem Cell Reports
Article
OPEN ACCESS

The Periostin/Integrin-αv Axis Regulates the Size of Hematopoietic Stem Cell Pool in the Fetal Liver

Atreyi Biswas,[1] Irene M. Roy,[1] Prathibha C. Babu,[1,6] Javed Manesia,[2,7] Sarah Schouteden,[2] Vinod Vijayakurup,[3] Ruby John Anto,[3] Joerg Huelsken,[4] Adam Lacy-Hulbert,[5] Catherine M. Verfaillie,[2] and Satish Khurana[1,*]

[1]School of Biology, Indian Institute of Science Education and Research Thiruvananthapuram, Thiruvananthapuram, Kerala 695551, India
[2]Inter-Departmental Stem Cell Institute, KU Leuven, 3000 Leuven, Belgium
[3]Rajiv Gandhi Centre for Biotechnology, Poojappura, Thiruvananthapuram, Kerala, India
[4]École Polytechnique Fédérale de Lausanne (EPFL), Lausanne, Switzerland
[5]Benaroya Research Institute at Virginia Mason, Seattle, WA 98101, USA
[6]Present address: Center for Stem Cell Research, Vellore, India
[7]Present address: Canadian Blood Services, Ottawa, Canada
*Correspondence: satishkhurana@iisertvm.ac.in or svkanand@gmail.com (S.K.)
https://doi.org/10.1016/j.stemcr.2020.06.022

SUMMARY

We earlier showed that outside-in integrin signaling through POSTN-ITGAV interaction plays an important role in regulating adult hematopoietic stem cell (HSC) quiescence. Here, we show that *Itgav* deletion results in increased frequency of phenotypic HSCs in fetal liver (FL) due to faster proliferation. Systemic deletion of *Postn* led to increased proliferation of FL HSCs, albeit without any loss of stemness, unlike *Vav-Itgav*$^{-/-}$ HSCs. Based on RNA sequencing analysis of FL and bone marrow HSCs, we predicted the involvement of DNA damage response pathways in this dichotomy. Indeed, proliferative HSCs from *Postn*-deficient FL tissues showed increased levels of DNA repair, resulting in lesser double-strand breaks. Thus POSTN, with its expression majorly localized in the vascular endothelium of FL tissue, acts as a regulator of stem cell pool size during development. Overall, we demonstrate that the duality of response to proliferation in HSCs is developmental stage dependent and can be correlated with DNA damage responses.

INTRODUCTION

Emergence and maturation of hematopoietic stem cells (HSCs) takes place during embryonic development where fetal liver (FL) plays a central role (Muller et al., 1994). Whereas bone marrow (BM)-resident adult HSCs are largely quiescent, fetal HSCs show robust proliferation to create the required stem cell pool (Bowie et al., 2006; Haas et al., 2018). It has been shown conclusively that HSCs differ majorly in their transcriptional profile across developmental stages (Manesia et al., 2015; McKinney-Freeman et al., 2012). The appearance of transient hematopoietic sites is a hallmark feature of the hematopoietic system during ontogeny and involves major changes in the HSC microenvironment across developmental stages (Boulais and Frenette, 2015). Little is known about fetal HSC niches, but recent evidence from Paul Frenette's group has shown the importance of cells associated with portal vessels in supporting HSCs in the FL tissue (Khan et al., 2016). This study follows closely another report from the same group wherein the arteriolar niche was shown to play host to the most primitive HSCs (Kunisaki et al., 2013). Significant genetic differences were observed in the cells that supported proliferative HSCs in FL tissue and the niche cells of the adult BM-derived HSCs (Khan et al., 2016). These differences notwithstanding, it has been sufficiently well established that association with their niches is crucial to the optimal functioning of fetal as well as adult HSCs (Gao et al., 2018).

Disruption of the interaction between HSCs and their microenvironment leads to deregulated proliferation, skewed differentiation, and decreased engraftment potential (Kiel and Morrison, 2008). Loss of integrin-α4, important for physical retention of HSCs in their niche, resulted in cell-cycle entry and spontaneous differentiation (Arroyo et al., 1999). Maintenance of quiescence in adult HSCs has been correlated with preservation of their long-term potential (Khurana, 2016). Repeated induction of proliferation in HSCs resulting from infection or chronic bleeding led to DNA damage and, ultimately, functional decline (Walter et al., 2015). However, as DNA damage responses (DDRs) are also linked to DNA replication process, Mohrin et al. (2010) showed that error-prone DDR pathways in quiescent HSCs lead to accumulation of DNA damage. By contrast, Beerman et al. (2014) showed that proliferative events in aged HSCs may lead to repair of damaged DNA. Thus, the precise link between proliferative events and stemness in the hematopoietic system remains unclear.

Integrins are some of the most crucial cell surface receptors that mediate retention of HSCs in the BM niche (Crane et al., 2017; Hoggatt et al., 2016; Khurana et al., 2013a). In addition, these receptors also mediate signaling cascades induced by growth factors or cytokines through outside-in signaling (Hynes, 2002). Whereas the importance of

This is an open access article under the CC BY license (http://creativecommons.org/licenses/by/4.0/).

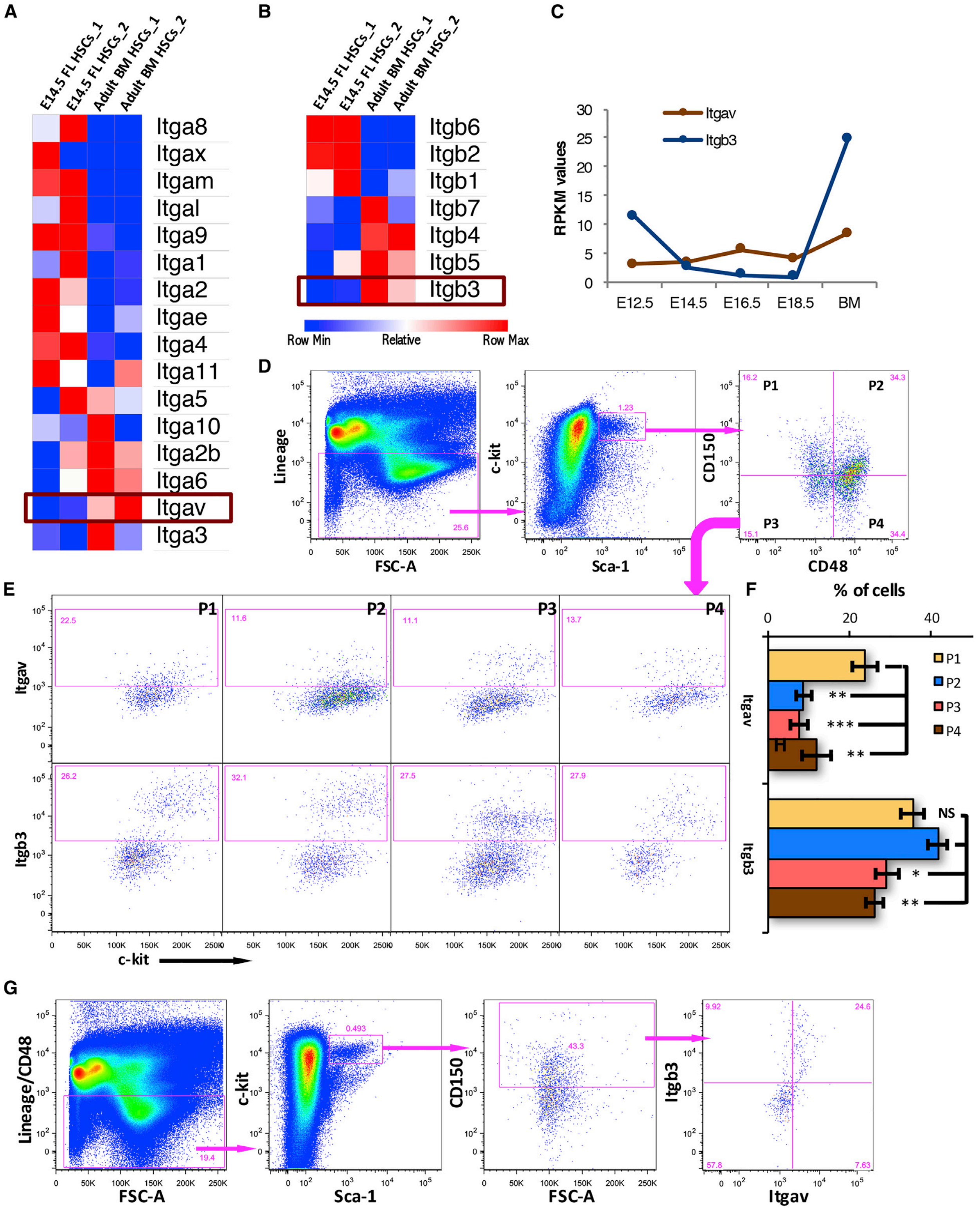

Figure 1. Expression of ITGAV and ITGB3 in FL HSCs

Gene and protein expression for both α- and β-integrin chains that make a heterodimeric receptor for POSTN, analyzed using RNA-seq and flow cytometry, respectively.

(legend continued on next page)

inside-out integrin signaling that regulates cell-cell or cell-extracellular matrix (ECM) adhesion is well worked out, the role of ligand-mediated outside-in signaling in hematopoiesis is not very well studied. Osteopontin, which elicits outside-in signaling cascades through integrin-αv (ITGAV; CD51) binding (Urtasun et al., 2012), acts as a negative regulator of HSC expansion (Nilsson et al., 2005; Stier et al., 2005). Integrin-αvβ3 (ITGAV-B3) also has a synergistic negative effect with the pro-inflammatory cytokine interferon-γ on HSC function (Umemoto et al., 2017). ITGB3 expression has been correlated with HSC quiescence (Umemoto et al., 2006) and ITGB3^{hi}CD34^{-}LSK cells were shown to be enriched for long-term repopulating HSCs (Umemoto et al., 2008). Periostin (POSTN) (Gillan et al., 2002) and Del1 (Mitroulis et al., 2017; Penta et al., 1999) have also been shown to mediate outside-in integrin signaling via binding to the ITGAV-B3 heterodimer. We recently reported that systemic deletion of *Postn* or *Vav-iCre* mediated conditional deletion of *Itgav* leads to the loss of quiescence in primitive HSCs, ultimately resulting in functional decline (Khurana et al., 2016).

Here, we report that the interruption of POSTN-ITGAV interaction causes increased proliferation of FL HSCs without any loss of stemness, resulting in their efficient expansion. This was unlike the effect of increased HSC proliferation on adult HSC function, indicating a developmental stage-specific response to proliferation rate. Our results linked better DDR in fetal HSCs with enhanced tolerance to proliferation stress. Overall, we show that the effect of proliferation on stemness is developmental stage dependent and is linked with DDR pathways.

RESULTS

Expression of αv and β3 Integrin Chains in FL-Derived Primitive HSCs

We first examined the expression of ITGAV and its binding partner ITGB3 in embryonic day 14.5 (E14.5) FL HSCs (lin^{-}c-kit^{+}Sca-1^{+}CD48^{-}CD150^{+} cells; Figure 1). We analyzed our previously published RNA sequencing (RNA-seq) data (Manesia et al., 2015) to compare the expression of all known α-integrin (Figure 1A) and β-integrin (Figure 1B) chains. The heatmap analysis showed lower expression of both *Itgav* and *Itgb3* in E14.5 FL-derived HSCs. In fact, the expression of *Itgav* and *Itgb3* was observed to be low in HSCs from all embryonic stages (Figure 1C, S1A, and S1B), consistent with our earlier published results that established POSTN-ITGAV interaction as a negative regulator of BM-HSC proliferation. Importantly, we detected high levels of expression of integrins, such as *Itga4*, *Itga5*, *Itga6*, *Itgb1*, which are known to play an important role in HSC function, in FL-derived HSCs (Figures S1A and S1B). To confirm the relative abundance of *Itgav* and *Itgb3* in BM versus E14.5 FL HSCs, we performed qRT-PCR using freshly sorted cells (Figure S1C). We confirmed that the transcript levels of both *Itgav* and *Itgb3* were significantly higher in the BM versus FL HSCs.

We next performed flow cytometry analysis of FL-derived mononuclear cells (MNCs) to examine the expression of ITGAV and ITGB3 in different sub-populations within the stem cell compartment (Figures 1D–1F, S1D, and S1E). On the basis of expression of SLAM markers CD150 and CD48, the lin^{-}c-kit^{+}Sca-1^{+} (LSK) population (isotype antibody controls are shown in Figure S1D) was subdivided into four sub-populations; CD150^{+}CD48^{-} (P1), CD150^{+}CD48^{+} (P2), CD150^{-}CD48^{-} (P3), and CD150^{-}CD48^{+} (P4) (Figure 1D). Subsequently, the expression of ITGAV (upper panel) as well as ITGB3 (lower panel) in each of these populations was assessed (Figure 1E; details of gating strategies with isotype antibody and FMO controls in Figures S1D and S1E). Results showed that 23.80% ± 3.21% of the most primitive HSCs (P1) from E14.5 FL MNCs expressed Itgav (Figure 1F). The expression of ITGAV in all other sub-populations was lower than the primitive HSC population ($p = 0.002$; one-way ANOVA followed by Tukey-Kramer test). As Itgav has been shown to partner with ITGB3 to form a heterodimeric integrin receptor in a

(A and B) Heatmaps showing differential expression of all known α-integrin (A) and β-integrin (B) chains analyzed by RNA-seq of primitive HSCs from E14.5 FL and adult BM. CD150^{+}CD48^{-} LSK cells were sorted out from the two stages to perform paired end sequencing, reported in our earlier study.
(C) *Itgav* and *Itgb3* expression in HSCs sorted from different developmental stages. Raw reads were subjected to quality control and high quality reads were aligned to mouse reference genome mm9. Reads per kilobase per million (RPKM) values obtained for *Itgav* and *Itgb3* expression across developmental stages were plotted.
(D) E14.5 FL cells were analyzed for the cell surface expression of ITGAV and ITGB3 on various HSC sub-populations. Lin^{-}c-kit^{+}Sca-1^{+} (LSK) population was further divided into four sub-populations on the basis of expression of SLAM markers CD48 and CD150.
(E) All four sub-populations of the LSK cells were examined for the expression of ITGAV (upper panel) and ITGB3 (lower panel), separately.
(F) Proportion of different HSC sub-populations that expressed ITGAV and ITGB3. n = 4, one-way ANOVA followed by Tukey-Kramer post hoc test: $*p < 0.05$, $**p \leq 0.01$, $***p \leq 0.005$, NS, not significant.
(G) Co-expression analysis of ITGAV and ITGB3 on the primitive FL HSCs. The E14.5 FL-derived HSCs were identified as lin^{-}CD48^{-}c-kit^{+}Sca-1^{+}CD150^{+} cells and were examined for the expression of ITGAV and ITGB3 based on flow cytometry.
See also Figure S1.

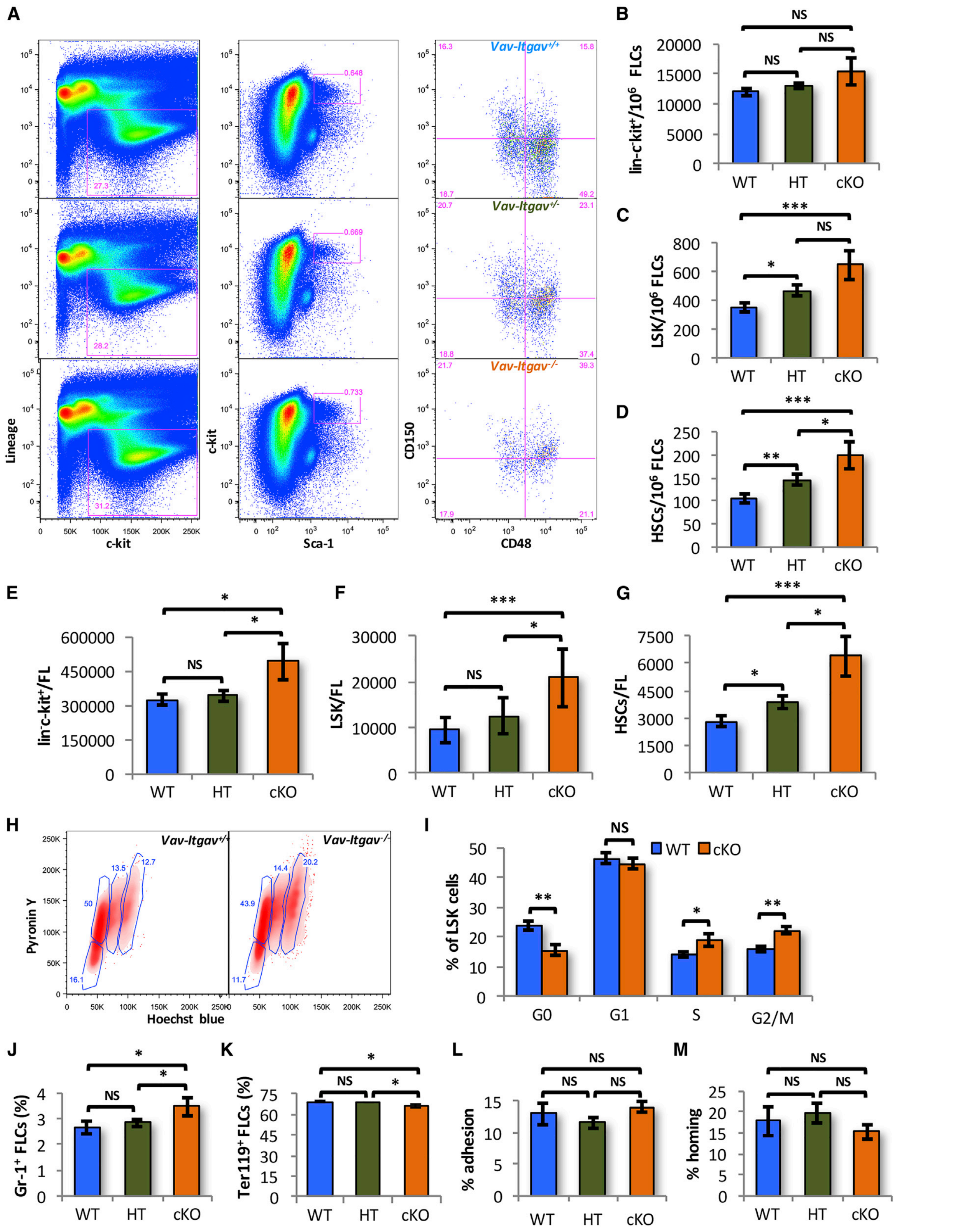

(legend on next page)

variety of cells (Savill et al., 1990), including a number of cancers (Eliceiri and Cheresh, 2001), we investigated the expression of ITGB3 in the stem cell sub-populations. The CD150$^+$CD48$^-$ (P1; 35.3% ± 2.72%) and CD150$^+$ CD48$^+$ sub-populations (P2; 41.57% ± 6.17%) expressed significantly higher levels of ITGB3 than CD150$^-$CD48$^-$ (P3; 29.06% ± 2.90%) and CD150$^-$CD48$^+$ (P4; 26.13% ± 1.97%) sub-populations (Figure 1F, p = 0.008; one-way ANOVA followed by Tukey-Kramer test). We then examined the level of co-expression of ITGAV and ITGB3 on the most primitive HSC population (CD150$^+$CD48$^-$ LSK population). We observed that 76.25% ± 6.8% of the primitive HSCs from E14.5 FL that expressed ITGAV also expressed ITGB3 (Figure 1G; details of gating strategies with isotype antibody controls in Figures S1D–S1F). Therefore, notwithstanding lower transcript levels of *Itgav* and *Itgb3* in primitive HSCs all across FL stages when compared with the adult BM-derived cells, we observed a significant level of cell surface expression of both ITGAV and ITGB3.

Loss of ITGAV in the Hematopoietic System Leads to Increased Frequency of Phenotypic FL Hematopoietic Stem and Progenitor Cells

To understand the role of POSTN-ITGAV interaction, we conditionally deleted *Itgav* in the hematopoietic system by crossing *Itgav*$^{fl/fl}$ (Lacy-Hulbert et al., 2007) with *Vav*-iCre mice (de Boer et al., 2003) (Figure S2A). Tissue from the hindlimb bud of each E14.5 fetus was kept for genotyping to detect the wild-type (WT) or floxed *Itgav* allele (Figure S2B) and the *Vav*-iCre (Figure S2C) transgene. Genotyping confirmed normal monohybrid Mendelian genotypic ratios between *Vav-Itgav*$^{+/+}$ (WT; 26.67%), *Vav-Itgav*$^{+/-}$ (heterozygous [HT]; 48.33%), and *Vav-Itgav*$^{-/-}$ (conditional knockout [cKO]; 25%) fetuses ($\chi^2_{df=2} = 0.049$, p = 0.98) (Figure S2D) that appeared healthy with no gross change in morphology (Figure S2E). We did observe modest but significant increase in total FL cellularity upon biallelic deletion of *Itgav* (Figure S2F). We then confirmed the lack of *Itgav* expression in the sorted LSK population by qRT-PCR (Figures S3A and S3B). In addition, the absence of cell surface expression of ITGAV in the primitive HSC population (SLAM LSK cells) was confirmed by flow cytometry (Figure S3C).

Next, we compared the frequency of hematopoietic stem and progenitor cells (HSPCs) (lin$^-$c-kit$^+$ cells), LSK cells, and primitive HSCs in E14.5 FL tissues (Figures 2A–2D). Although we did not observe any significant change in the frequency of lin$^-$c-kit$^+$ cells (Figure 2B), we did observe a significant increase in the frequency of LSK cells (Figure 2C) as well as primitive HSCs (Figure 2D) following *Itgav* deletion. Interestingly, even monoallelic deletion of *Itgav* (HT) resulted in an increase in the frequency of LSK and primitive HSCs (Figures 2C and 2D). As we observed increased total FL cellularity upon *Itgav* deletion, we compared the total numbers of HSPCs, LSK cells, and HSCs (SLAM LSK cells) per FL (Figures 2E–2G). We observed an increase in the number of LSK cells and primitive HSC population in *Vav-Itgav*$^{-/-}$ FL tissues. Following monoallelic deletion of *Itgav*, however, we could see an increase in the primitive HSC population only (Figure 2G). We then addressed if this increase in the frequency of HSPC populations was accompanied by an altered proliferation status. Cell-cycle analysis of the FL cells using Hoechst and Pyronin Y staining (Figures 2H and 2I) showed only 23.63% ± 1.57% of the LSK cells to be quiescent. In *Vav-Itgav*$^{-/-}$ fetuses, we observed a significant decrease in the proportion (15.58% ± 1.74%) of quiescent LSK cells (Figure 2I). This was accompanied by an increase in the

Figure 2. Increased Frequency of Phenotypic HSCs in E14.5 *Vav-Itgav*$^{-/-}$ FL Tissues

(A) *Vav*-iCre and *Itgav*$^{fl/fl}$ mice were crossed to conditionally delete *Itgav* in hematopoietic system. The embryos were harvested at E14.5 and genotyped to identify *Vav-Itgav*$^{+/+}$ (WT), *Vav-Itgav*$^{+/-}$ (HT), *Vav-Itgav*$^{-/-}$ (cKO) embryos. FL tissue was used for analysis of HSC frequency in by flow cytometry using specific antibodies.

(B–D) Quantification of frequency of various hematopoietic stem and progenitor cell populations in the FL tissue from WT, HT, and cKO embryos at E14.5; (B) lin$^-$c-kit$^+$ cells, (C) LSK cells, (D) primitive HSCs.

(E–G) Comparison of the total number of hematopoietic stem and progenitor cells per FL from WT, HT, and cKO embryos at E14.5; (E) lin$^-$c-kit$^+$ cells, (F) LSK cells, (G) primitive HSCs. (H) Cell-cycle analysis of the LSK cells harvested from E14.5 FL tissues by Hoechst 33342/pyronin Y staining (n = 6).

(I) Comparison of the proportion of FL-derived LSK cells in various stages of cell cycle.

(J and K) Flow cytometry-based analysis performed to compare the frequency of lineage-committed cells in E14.5 FL tissue from WT, HT, and cKO embryos. (J) Granulocytes identified as Gr-1$^+$ cells, (K) erythrocytes identified as Ter119$^+$ cells.

(L) Adhesion potential of E14.5 *Vav;Itgav*$^{+/+}$ (WT), *Vav;Itgav*$^{+/-}$ (HT), and *Vav;Itgav*$^{-/-}$ (cKO) FL-derived LSK cells was compared through *in vitro* adhesion assays. Freshly isolated LSK cells were allowed to adhere on ST2 cell feeder. The percentage of cells that adhered to the feeder after 3 h was plotted for each condition (n = 4).

(M) Whole FL cells from *Vav;Itgav*$^{+/+}$ (WT), *Vav;Itgav*$^{+/-}$ (HT), and *Vav;Itgav*$^{-/-}$ (cKO) mice, were infused in lethally irradiated animals. The percentage of transplanted colony-forming cells that homed into the BM within 16 h was plotted (n = 2, N = 8).

An unpaired two-tailed Student's t test was performed. n = 2–4, N = 8–14, t test: $^*p < 0.05$, $^{**}p \leq 0.01$, $^{***}p \leq 0.005$. See also Figures S2 and S3.

proportion of LSK cells in S phase from 13.96% ± 1.03% in WT to 19.06% ± 2.26% in *Vav-Itgav*$^{-/-}$ fetuses. Similar increase in the percentage of FL LSK cells in G2/M phase of cell cycle was also observed (16.02% ± 1.06% in WT to 22.23% ± 1.42% in cKO). We did not detect any changes in the proportion of these cells in the G1 phase of cell cycle (Figure 2I). These results indicated that the loss of *Itgav* expression in the hematopoietic cells led to increased proliferation rates.

We then performed flow cytometry-based analysis of FL-derived MNCs to detect any differences in the lineage-committed cell populations upon *Itgav* deletion (Figures 2J, 2K, and S3D–S3G). Results demonstrated a modest but significant increase in the proportion of Gr-1^{+} cells in *Vav-Itgav*$^{-/-}$ FL tissues (Figures 2J and S3D, upper panel). We did not observe any change in the macrophage population identified by F4/80 expression (Figure S3E). Evaluation of B and T cell lineages identified as CD19^{+} (Figure S3F) and CD3e^{+} cells (Figure S3G) respectively, showed no change upon *Itgav* deletion. We also examined whether the proportion of erythroid population that makes the bulk of FL cellularity is changed upon *Itgav* deletion (Figures 2K and S3D, lower panel). We observed that biallelic deletion of *Itgav* led to a modest but significant decrease in the erythroid cell population from 69.03% ± 0.58% to 65.08% ± 2.21%.

As integrins are known for their role as cell surface adhesion receptors, we examined if the lack of ITGAV had any effect on stem cell attachment that could affect their homing and engraftment. We performed *in vitro* adhesion assays using ST2 cell feeders and PKH26-labeled LSK cells. We observed no effect of *Itgav* deletion, on the proportion of the cells that adhered to the feeder layer after a 3-h incubation (Figure 2L). We also performed short-term *in vivo* assays to examine if there was any defect in their homing potential. Total E14.5 FL-derived cells were transplanted in lethally irradiated animals and the proportion of colony-forming unit cells that homed in the BM within 16 h of transplantation was quantified (Figure 2M). Comparison showed no effect of loss of *Itgav* on the homing potential of the FL-derived hematopoietic stem and progenitor population.

Loss of *Itgav* in FL HSCs Results in Poor Long-Term Engraftment

After confirming that *Itgav* deficiency led to an increase in the frequency of phenotypic HSCs in FL tissue, we examined their functional properties using competitive repopulation assays. First, 10,000 WT, HT, or cKO FL MNCs together with 90,000 supporting CD45.1 whole BM cells were transplanted into lethally irradiated CD45.1 recipients (Figure 3A). The primary recipients that were transplanted with *Vav-Itgav*$^{-/-}$ FL cells consistently showed increased chimerism compared with the animals that received WT cells (Figures 3B–3D). Monoallelic deletion of *Itgav* did not result in any change in donor-derived chimerism 8 weeks after transplantation (Figures 3B and 3C). After 12 weeks of transplantation, there was no difference in the level of chimerism between animals that received *Vav-Itgav*$^{-/-}$ or *Vav-Itgav*$^{+/-}$ FL cells (Figure 3D). To test the long-term engraftment potential, we transplanted BM cells from primary recipients into lethally irradiated secondary recipients. Surprisingly, 12 weeks after secondary transplantation, we did not observe any differences between the donor-derived chimerism in animals that received BM cells from primary recipients of the WT, HT, or cKO cells (Figure 3E). We did not observe any significant change in the multi-lineage engraftment potential following *Itgav* deletion (Figure 3F). Thus, the results from *in vivo* repopulation studies indicated an increase in the frequency of only short-term repopulating FL HSCs resulting from the loss of *Itgav*.

To test the function of the HSC population in response to *Itgav* deletion, we repeated the long-term repopulation assays using sorted HSCs. We transplanted 100 fluorescence-activated cell sorting (FACS)-sorted FL HSCs (CD150^{+}CD48^{-} LSK cells) from *Vav-Itgav*$^{+/+}$, *Vav-Itgav*$^{+/-}$, or *Vav-Itgav*$^{-/-}$ fetuses, together with 100,000 CD45.1 WBMCs cells into lethally irradiated CD45.1 recipient mice (Figure 3A). No differences were observed among the three groups of primary recipients for up to 8 weeks of transplantation (Figures 3G and 3H). However, there was a significant increase in the donor-derived chimerism from *Vav-Itgav*$^{-/-}$ FL HSCs at 12 weeks after transplantation in primary recipients (Figure 3I). After 12 weeks of transplantation, 10×10^5 BM cells from the primary recipients were transplanted into lethally irradiated secondary recipients. Interestingly, we observed that donor-derived chimerism was lower in the cohort that received BM cells from primary recipients transplanted with the *Vav-Itgav*$^{-/-}$ HSCs (Figure 3J). These results indicated that the FL HSCs showed poor long-term engraftment in the absence of *Itgav* expression, consistent with our previously published findings on adult BM HSCs (Khurana et al., 2016). Importantly, multi-lineage engraftment potential was also affected in HSCs; however, only following biallelic deletion of *Itgav* (Figure 3K). While there was an increase in the myeloid population within the donor-derived peripheral blood (PB) cells, there was an almost proportionate decrease in the cells from B cell lineage. We observed no difference in the donor-derived contribution to T cell differentiation (Figure 3K).

Systemic Deletion of *Postn* Leads to Expansion of Functional HSCs in FL

The fetal HSCs transition to an adult phenotype within a few weeks after engraftment, masking any effects of *Itgav*

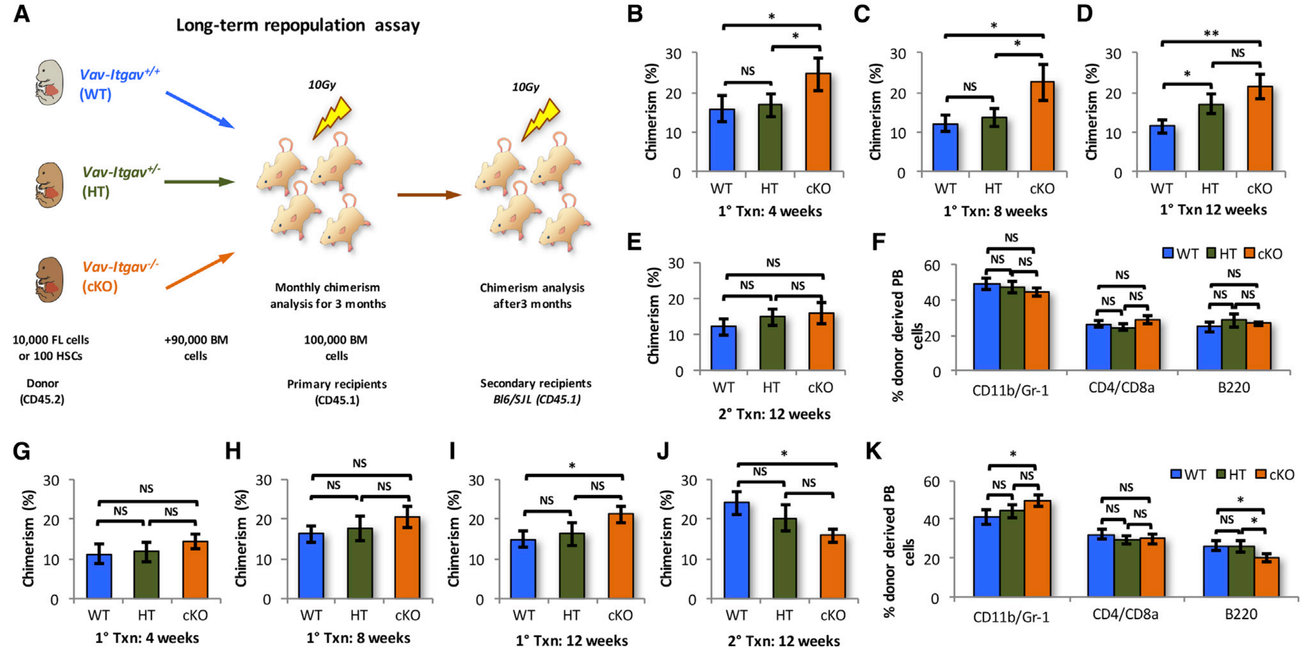

Figure 3. *Vav-Itgav*$^{-/-}$ HSCs Acquire Adult Phenotype During Engraftment and Show Functional Decline

(A) Schematic representation of the competitive repopulation assay performed to assess the function and/or frequency of HSCs in FL tissue following *Itgav* deletion. A total of 10,000 whole FL cells from WT, HT, and cKO embryos along with 90,000 whole BM competitor cells (CD45.1) were transplanted into lethally irradiated WT CD45.1 mice. Alternatively, the assays were performed using sorted primitive HSCs. One hundred CD150^{+}CD48^{-}KLS cells together with 100,000 whole BM competitor cells (CD45.1) were transplanted into sub-lethally irradiated WT CD45.1 mice. Peripheral blood (PB) chimerism and multi-lineage engraftment was followed for 12 weeks, after which secondary transplantations were performed. Secondary recipients were analyzed after 3 months of transplantation.

(B–D) PB chimerism in primary recipients compared after 4 weeks (B), 8 weeks (C), and 12 weeks (D).

(E) PB chimerism in secondary recipients after 12 weeks of transplantation.

(F) Multi-lineage engraftment for myeloid (CD11b/Gr-1), T cell (CD4/CD8), and B cell (B220) lineage in secondary recipients after 12 weeks of transplantation.

(G–I) Chimerism analysis of primary recipients transplanted with 100 sorted HSCs from WT, HT, and KO embryos, together with 100,000 competitor cells. PB chimerism was analyzed after (G) 4 weeks, (H) 8 weeks, and (I) 12 weeks of transplantation.

(J) Chimerism analysis of the secondary recipients transplanted with the BM cells of primary recipients that received freshly sorted HSCs. PB chimerism was analyzed after 12 weeks of transplantation.

(K) Multi-lineage engraftment in secondary recipients after 12 weeks of transplantation. Analysis was performed for myeloid (CD11b/Gr-1), T cell (CD4/CD8), and B cell (B220) lineages.

An unpaired two-tailed Student's t test was performed. n = 3, N = 15–18, t test: *$p < 0.05$, **$p \leq 0.01$, NS, not significant.

deletion on fetal HSCs. Therefore, we examined the effects of loss of POSTN-ITGAV interaction on HSC function using cells from *Postn*$^{-/-}$ FL tissues. While, we did not observe any major developmental defects upon *Postn* deletion at E14.5, we did observe a modest decrease in the overall size of the embryos (Figure 4A). This was also reflected in a marginal, but significant decrease in the weight of *Postn*-deficient fetuses (KO) at E14.5 (Figure 4B). Before the characterization of the hematopoietic system in *Postn*$^{-/-}$ FL tissues, we confirmed the lack of *Postn* at transcript and protein levels using qRT-PCR (Figure S4A) and immunohistochemistry (Figure S4B), respectively. We could not detect *Postn* expression in the FL tissue either at the transcript or at the protein level. We observed a modest decrease in the total cellularity of *Postn*$^{-/-}$ FL tissue as compared with the WT (Figure 4C). Thereafter, we examined if there was any effect of *Postn* deletion on lineage-committed cell populations (Figures S4C–S4F). We observed no significant change in the proportion of granulocyte (Gr-1^{+}; Figure S4C), macrophage (F4/80^{+}; Figure S4D), B cell (B220^{+}; Figure S4E), or T cell (CD3e^{+}; Figure S4F) populations in the FL tissues from *Postn*$^{-/-}$ fetuses. We next tested if there was any effect of *Postn* deficiency on the frequency of HSCs in the FL tissue (Figures 4D–4G). We did not observe any difference in the number of lin^{-}c-kit^{+} HSPC population between WT and KO fetuses (Figure 4E). However, analysis showed increase in the frequency of LSK cells (Figure 4F) as well as the

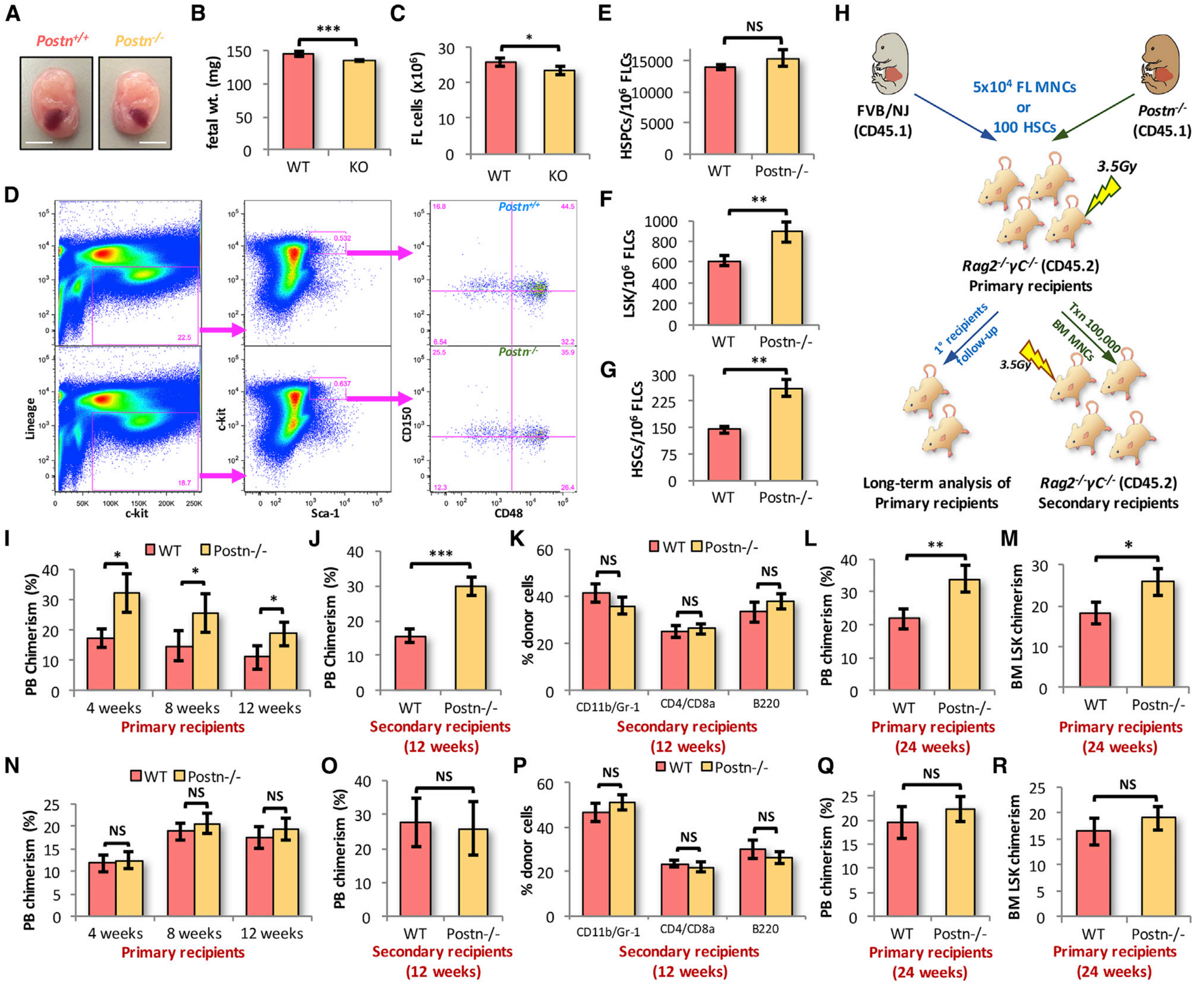

Figure 4. POSTN Deficiency Leads to Expansion of Functional HSCs in FL Tissue

(A) *Postn*$^{+/+}$ (WT) and *Postn*$^{-/-}$ (KO) embryos were harvested at E14.5, cleared of the extra-embryonic layers and gross morphology was compared. Scale bars, 5 mm.

(B) Individual embryos were weighed and overall weight of the WT and KO embryos was compared.

(C) Mononuclear cells from the E14.5 FL tissues were harvested and counted for the WT and KO embryos.

(D–G) The mononuclear cells harvested from the FL tissue of WT and KO embryos were analyzed for the frequency of HSC sub-populations (D) by flow cytometry. The frequency of lin^{-}c-kit^{+} cells (E), LSK cells (F), and primitive HSCs (G) in E14.5 FL mononuclear cells was compared between WT and *Postn*$^{-/-}$ embryos. n = 6–13.

(H) Schematic representation of the competitive repopulation assays. A total of 50,000 total E14.5 FL cells or 100 E14.5 FL-derived FACS-sorted SLAM LSK cells from *Postn*$^{+/+}$ (WT) or *Postn*$^{-/-}$ embryos (CD45.1) was transplanted into sub-lethally irradiated *Rag2*$^{-/-}$*γC*$^{-/-}$ mice (CD45.2). PB chimerism was followed for 12 weeks, after which secondary transplantations were performed using BM cells from half of the primary recipients. The secondary recipients were analyzed for PB chimerism after 12 weeks of transplantation. The PB chimerism in the rest of the primary recipients was analyzed for up to 24 weeks of transplantation. Donor-derived chimerism in BM LSK population was analyzed in moribund mice. n = 3, N = 15–18.

(I) Donor-derived PB chimerism in primary recipients analyzed after 4, 8, and 12 weeks of transplantation.

(J) After 12 weeks of transplantation of BM cells from primary recipients, secondary recipients were analyzed for PB chimerism.

(K) Multi-lineage engraftment in secondary recipients after 12 weeks of transplantation.

(L) After 24 weeks of transplantation, donor-derived PB chimerism in primary recipients that received whole FL cells from WT or KO embryos.

(legend continued on next page)

primitive HSCs (Figure 4G). As there was a modest change in the FL cellularity, we also analyzed the total number of cells in each of these populations, namely $lin^{-}c\text{-}kit^{+}$ HSPCs (Figure S4G), LSK (Figure S4H), and HSCs (Figure S4I), and observed similar effects upon *Postn* deletion.

To assess hematopoietic function, we performed *in vivo* repopulation assays (Figure 4H). Due to non-availability of suitable CD45.2 congenic mouse line in FVB/NJ background, immune-deficient $Rag2^{-/-}\gamma C^{-/-}$ mice were used as recipients. Fifty thousand E14.5 FL-derived cells were transplanted into sub-lethally irradiated $Rag2^{-/-}\gamma C^{-/-}$ mice. No competitor cells were transplanted, as the residual hematopoietic cells ($CD45.2^{+}$ cells against $CD45.1^{+}$ donor-derived cells) following a sub-lethal dose of radiation provided competition to the donor cells. The primary recipients that received FL cells from $Postn^{-/-}$ embryos showed higher levels of donor-derived chimerism (Figures 4I and S4J). After 12 weeks, the BM cells from half of the primary recipients were transplanted into sub-lethally irradiated secondary recipients and the donor-derived chimerism was followed for another 3 months. We observed a similar increase in donor-derived chimerism in secondary recipients as well (Figure 4J). However, we did not observe any change in the multi-lineage engraftment of the transplanted cells (Figure 4K). There was no observable difference in the proportion of cells from myeloid, T and B cell lineage within the donor-derived PB cells (Figure 4K). Analyses were continued for the second half of the primary recipients for long-term engraftment analysis. We observed a similar increase in donor-derived PB chimerism in the animals that received FL cells from $Postn^{-/-}$ embryos (Figure 4L). We also examined the donor-derived chimerism in the BM LSK population in moribund mice (Figures 4M and S4K). On expected lines, we observed a higher proportion of donor-derived BM LSK cells in the KO FL cell-transplanted group, although less pronounced than PB chimerism.

To confirm if the increased repopulation potential was due to increased hematopoietic function or HSC frequency, we performed long-term repopulation assays using sorted HSCs (Figure 4H). One hundred FACS-sorted primitive HSCs from WT or $Postn^{-/-}$ FL tissues were transplanted into sub-lethally irradiated $Rag2^{-/-}\gamma C^{-/-}$ mice. The results showed no change in donor-derived chimerism in primary (Figure 4N) or secondary recipients (Figure 4O) that were transplanted with HSCs from $Postn^{-/-}$ FL tissues as compared with WT. Importantly, multi-lineage engraftment potential of the transplanted HSCs also remained intact (Figure 4P). To assess the long-term repopulation potential of the HSCs from *Postn*-deficient FL tissues, we followed the primary recipients for up to 24 weeks. As indicated by the PB chimerism after 24 weeks, we did not observe any effect of *Postn* deletion on the long-term repopulation potential of the FL HSCs (Figure 4Q). In addition, we observed no change in the donor-derived chimerism in the BM LSK cell population after 24 weeks of transplantation (Figure 4R). These results indicated that the HSCs did not differ in their repopulation potential. Therefore, the increased level of chimerism after transplantation of total $Postn^{-/-}$ FL cells is likely due to increased frequency of HSCs and HSPCs.

FL HSCs Tolerate Culture-Induced DNA Damage Better Than BM HSCs

We hypothesized that the intrinsic differences between fetal and adult HSCs were the underlying reason for the difference in their tolerance to proliferation stress. Interestingly, from our earlier published RNA-seq data, we observed that mismatch repair (MMR), homologous recombination (HR), nucleotide excision repair (NER), and base excision repair (BER) were among the top 20 highly expressed pathways in E14.5 FL-derived HSCs compared with the adult BM-derived HSCs (Manesia et al., 2015). Heatmap analysis also showed increased expression levels of genes involved in MMR (Figure S5A), HR (Figure S5B), NER (Figure S5C), and BER (Figure S5D). Next, we aimed to examine the functional relevance of these pathways in the repair of culture-induced DNA damage (Figure 5). We

(M) The primary recipients analyzed after 24 weeks of transplantation for donor-derived chimerism in BM LSK population. Proportion of BM-derived cells in the total BM LSK cells was compared between the primary recipients that received whole FL cells from E14.5 WT or KO embryos (n = 3, N = 9–12).
(N) PB chimerism in primary recipients transplanted with 100 SLAM LSK cells from the E14.5 FL tissues.
(O) Secondary recipients analyzed for donor-derived PB chimerism 12 weeks after transplantation.
(P) Donor-derived multi-lineage engraftment in secondary recipients 12 weeks after transplantation.
(Q) Sorted HSCs from E14.5 WT or KO embryos were transplanted in sub-lethally irradiated animals. After 24 weeks of transplantation, donor-derived PB chimerism in primary recipients was compared between the two groups (n = 3, N = 7–11).
(R) The primary recipients analyzed after 24 weeks of transplantation for donor-derived chimerism in BM LSK population. Proportion of BM-derived cells in the total BM LSK cells was compared between the primary recipients that received freshly sorted HSCs from E14.5 WT or KO embryos (n = 3, N = 9–12).
An unpaired two-tailed Student's t test was performed. n = 3, N = 15–18, t test: $^{*}p < 0.03$, $^{**}p \leq 0.01$, $^{***}p \leq 0.005$, NS indicates not significant. See also Figure S4.

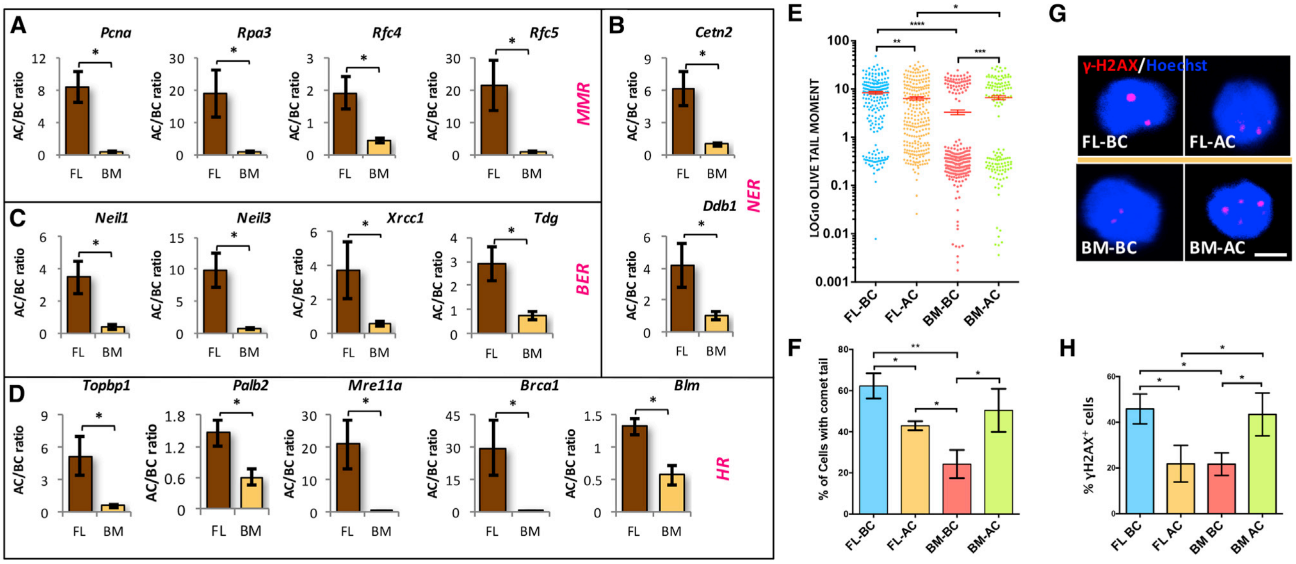

Figure 5. Elevated DNA Damage Response in FL-Derived HSCs against Culture-Induced Stress

(A–D) Bone marrow and E14.5 FL-derived KLS cells were cultured in serum-free medium in the presence of SCF and Tpo for 5 days. Expression for DDR genes from the harvested cells (after culture [AC]) was compared with the freshly isolated (before culture [BC]) LSK cells by performing qRT-PCR. Culture-induced change in the expression of genes that belong to different DDR pathways was plotted. (A) mismatch repair, *Pcna*, *Rpa3*, *Rfc4*, *Rfc5*; (B) nucleotide excision repair, *Cetn2*, *Ddb1*; (C) base excision repair, Neil1, Neil3, Xrcc1, Tdg; (D) homologous recombination, *Topbp1*, *Palb2*, *Mre11a*, *Brca1*, *Blm*. n = 4–6, t test: *$p < 0.05$.

(E and F) Neutral comet assay was performed using freshly isolated and cultured KLS cells from BM and FL tissues. Olive tail moment (E) and proportion of cells with comet tail (F) was compared for different samples. n = 4, N > 223, one-way ANOVA followed by Tukey's multiple comparisons test: *$p < 0.05$, **$p \leq 0.01$, ***$p \leq 0.005$.

(G) Immunostaining for γ-H2AX was performed on freshly isolated and cultured LSK cells from BM and E14.5 FL tissues. Scale bar, 5 μm.

(H) Proportion of cells with γ-H2AX for was compared between different samples. n = 4, N > 173, one-way ANOVA followed by Tukey's multiple comparisons test: *$p < 0.05$, **$p \leq 0.01$.

See also Figure S5.

sorted LSK cells from E14.5 FL as well as adult BM and cultured them in serum-free medium along with stem cell factor (SCF) and thrombopoietin for 5 days. This expansion medium induces proliferation of HSCs as reported previously (Khurana et al., 2013b; Sitnicka et al., 1996; Zhang and Lodish, 2005) and expectedly; we observed faster proliferation rates in FL-derived LSK cells (Figure S5E). The proliferative events also led to increase in the single- and double-strand DNA breaks, which are repaired during the replication process. We explored if the LSK cells isolated from FL and BM showed differences in the expression of key genes involved in DDR pathways before and after culture-induced proliferation (Figures 5A–5D). We observed clear upregulation of DDR pathway genes in FL HSCs upon culture. Among the genes involved in the MMR pathway, we observed increased expression of *Pcna*, *Rpa3*, *Rfc4*, and *Rfc5* (Figure 5A). From the NER pathway, *Cetn2* and *Ddb1* (Figure 5B), and from the BER pathway, *Neil1*, *Neil3*, *Xrcc1*, and *Tdg* (Figure 5C), showed similar increase in expression. Among HR-related genes, we tested *Topbp1*, *Palb2*, *Mre11a*, *Brca1*, and *Blm*, and all showed significantly higher levels of expression (Figure 5D). Importantly, when we examined BM-derived LSK cells for DDR gene expression following culture, the increase in the expression of all these DDR genes was significantly lower than what was observed for FL-derived cells.

Gene expression may not clearly reflect the status of functional activity of the repair pathways tested; therefore, we next examined if there was any improvement in DNA damage repair in these cells. Freshly sorted as well as cultured BM- and FL-derived LSK cells were used to perform the neutral comet assay (Figures 5E and 5F) to measure double-strand breaks. Olive tail moment is a measure of DNA damage that takes into consideration both the length of the comet tail and the proportion of total DNA it contains. We observed lower levels of DNA damage in freshly isolated BM-derived cells than their FL counterparts, likely due to higher proliferation rates in FL-derived cells. In contrast, the olive tail moment was significantly decreased when FL-derived LSK cells were cultured for a period of 5 days (Figure 5E). Contrary to this, we observed an increase in double-strand breaks in BM LSK cells upon culture. We

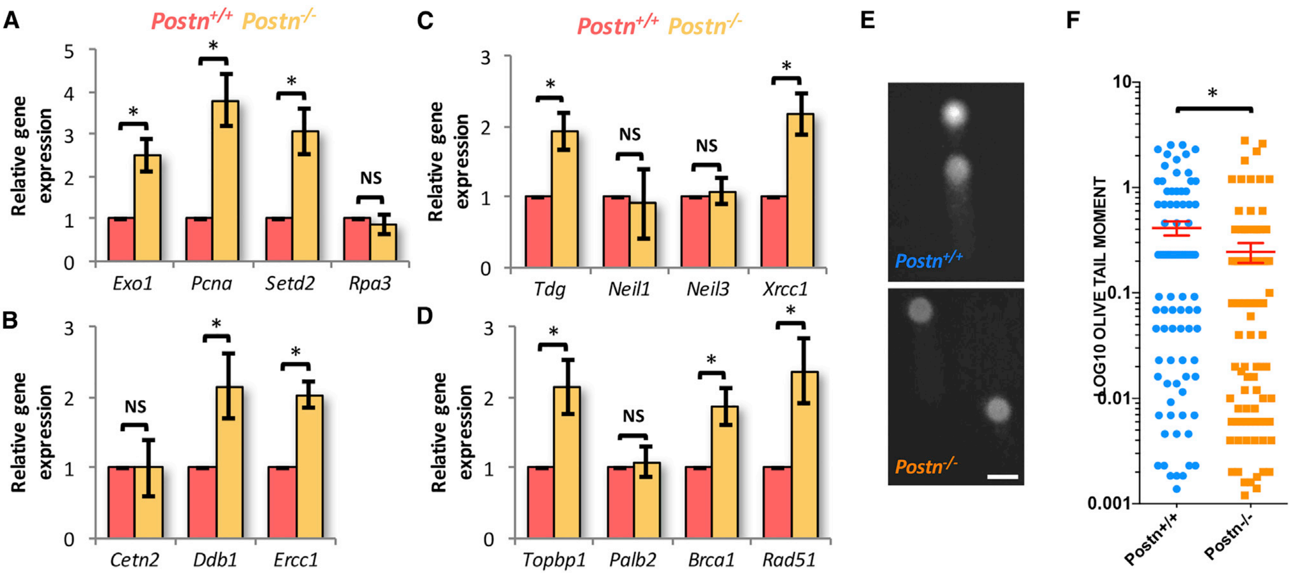

Figure 6. Better DNA Damage Repair in HSCs from *Postn*$^{-/-}$ FL Tissues

(A–D) *Postn*$^{+/+}$ and *Postn*$^{-/-}$ FL-derived LSK cells were used for assessing the expression of DNA damage response genes through qRT-PCR. Expression of genes that belong to MMR, such as *Exo1*, *Pcna*, *Setd2*, and *Rpa3* (A), NER, such as *Cetn2*, *Ddb1*, and *Ercc1* (B), BER, such as *Tdg*, *Neil1*, *Neil3*, and *Xrcc1* (C), and HR pathways, such as *Topbp1*, *Palb2*, *Brca1*, and *Rad51* (D) was examined. Gene transcript changes were normalized to *β-actin* levels and relative expression of respective genes was examined in LSK cells from *Postn*$^{-/-}$ FL tissues as compared with the *Postn*$^{+/+}$ FL-derived cells. Unpaired two-tailed Student's t test. n = 4–6. *p < 0.0055.

(E and F) Freshly sorted primitive HSCs (CD150^{+}CD48^{-} LSK cells) from E14.5 *Postn*$^{+/+}$ and *Postn*$^{-/-}$ FL tissues were used to perform neutral comet assay. (E) Representative comets from the HSCs from *Postn*$^{+/+}$ (upper panel) and *Postn*$^{-/-}$ (lower panel) FL tissues. Scale bar, 10 μm. (F) Olive tail moment compared between *Postn*$^{+/+}$ (N = 379) and *Postn*$^{-/-}$ (N = 307) FL HSCs. n = 4–6, Mann-Whitney U test: *p = 0.0407.

also examined the proportion of cells with a comet tail and observed similar results (Figure 5F). The proportion of FL-derived LSK cells with comet tail decreased after 5 days of culture while the BM-derived LSK cells showed a significant increase, indicating functional differences in DDR pathways. We confirmed these observations by performing immunostaining for γ-H2AX, a marker for DNA double-strand breaks (Figure 5G). Both the proportion of cells with γ-H2AX foci (Figure 5H) and number of the γ-H2AX foci per cell (Figure S5F) decreased in FL-derived cells after culture. BM-derived HSPCs on the other hand accumulated more DNA damage per cell. The results also showed that the proportion of cells with a higher number of foci (3–5 and >5) increased significantly when BM-derived cells were cultured (Figure S5G). Contrary to this, the DNA damage was more efficiently cleared upon culture of FL-derived HSPCs. These experiments confirmed an increased level of DDR activity in FL-derived HSPCs than BM.

Better DDR in HSCs from *Postn*$^{-/-}$ FL Tissues

Our results demonstrated that the proliferative FL HSCs were significantly better in their DDRs. These intrinsic differences resulted in increased levels of tolerance to proliferative stress in fetal HSCs as compared with their adult BM equivalents. We hypothesized that the expansion of functional HSCs in the FL tissues of *Postn*-deficient embryos could also be linked to better DNA damage repair. To test this hypothesis, we first checked the expression of some of the DDR genes, earlier identified in our screen, where HSCs isolated from FL and adult BM were compared (Figures S5A–S5D). We sorted LSK cells from FL tissues of *Postn*$^{+/+}$ and *Postn*$^{-/-}$ fetuses and performed qRT-PCR to examine the expression of genes that belong to MMR (*Exo1*, *Pcna*, *Setd2*, and *Rpa3*; Figure 6A), NER (*Cetn2*, *Ddb1*, and *Ercc1*; Figure 6B), BER (*Tdg*, *Neil1*, *Neil3*, and *Xrcc1*; Figure 6C), and HR pathways (*Topbp1*, *Palb2*, *Brca1*, and *Rad51*; Figure 6D). Some of these genes (*Rpa3* from MMR; *Cetn2* from NER; *Neil1*and *Neil3* from BER; and *Palb2* from HR) did not show any change in expression upon *Postn* deletion. However, several of them from MMR (*Exo1*, *Pcna*, and *Setd2*; Figure 6A), NER (*Ddb1* and *Ercc1*; Figure 6B), BER (*Tdg* and *Xrcc1*; Figure 6C), and HR pathways (*Palb2*, *Brca1*, and *Rad51*; Figure 6D) showed significant increase in LSK cells sorted from *Postn*$^{-/-}$ FL tissues. These results indicated a possible functional improvement in DNA damage repair. To test this, we performed neutral comet assays using HSCs (SLAM LSK cells) from *Postn*$^{+/+}$ and *Postn*$^{-/-}$ FL tissues (Figures 6E and 6F).

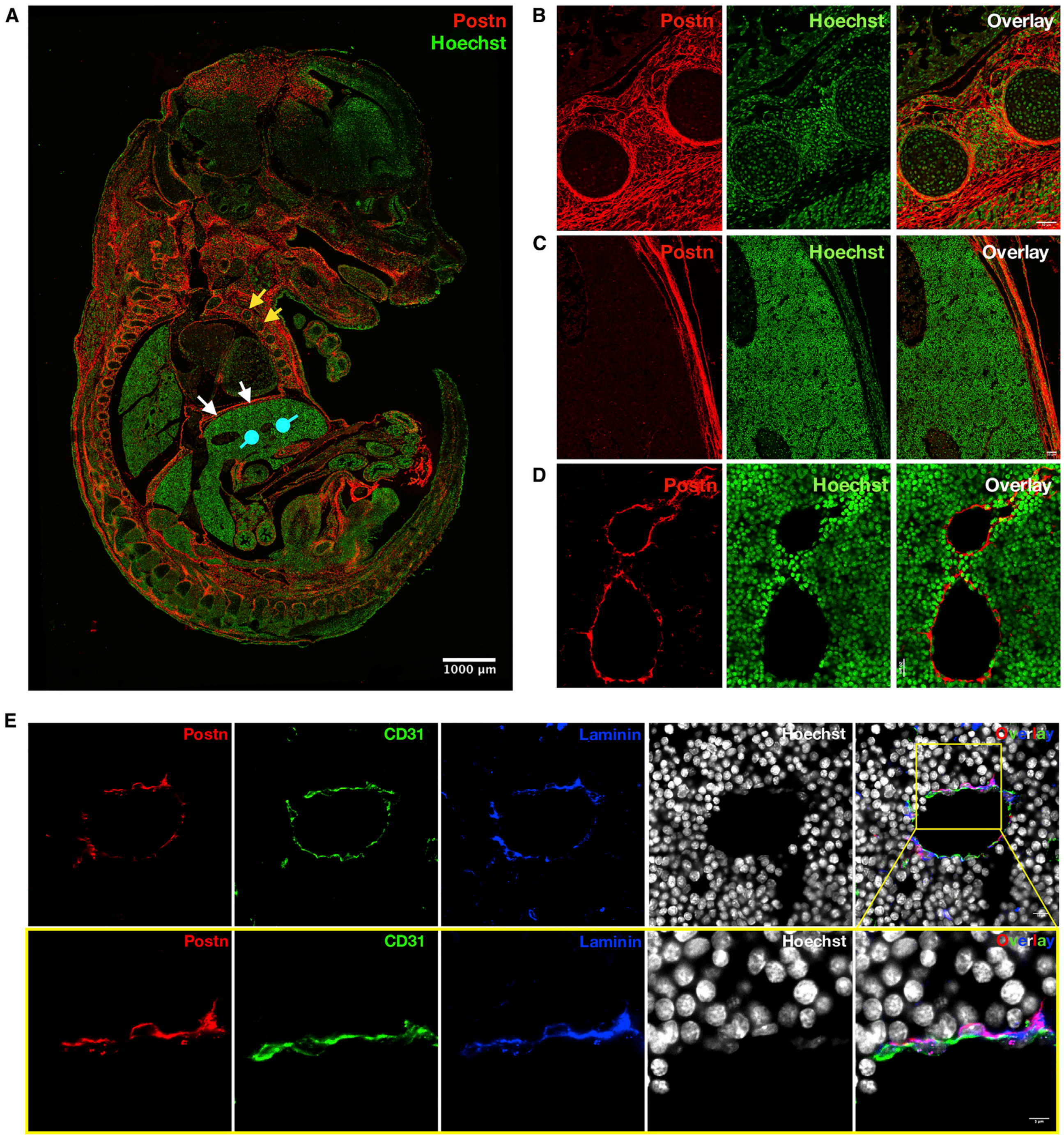

Figure 7. POSTN Is Expressed in Vascular Endothelium of the FL

(A) PSTN expression was examined in the E14.5 fetuses using immunohistochemistry on 10-µm formalin-fixed paraffin-embedded sections followed by confocal imaging. Specific antibodies were used to identify the cells expressing POSTN, nuclear counterstaining was done using Hoechst 33342. Regions with compelling levels of POSTN expression; namely, the developing ribs (yellow arrows), diaphragm (white arrows), and the tissue of interest, FL (blue round head arrows), are indicated (n = 4). Scale bar, 1 mm.

(B–D) Enlarged POSTN-expressing regions from the tiles were extracted. (B) Developing ribs showing marrow as well as the mesenchymal tissue (n = 3). Scale bar, 50 µm. (C) Muscular diaphragm (n = 3). Scale bar, 50 µm. (D) Vascular region in the FL tissue (n = 4). Scale bar, 20 µm.

(legend continued on next page)

Comparison of the olive tail moment of individual cells showed clear decrease in the DNA damage in the HSCs derived from *Postn*$^{-/-}$ FL tissues (Figure 6F; n = 3, N = 105–132, *p = 0.04). We concluded that, during embryonic development, proliferative stress is tolerated by increased levels of DDRs.

POSTN Is Expressed in Vascular Endothelium of the FL

Results presented above further established POSTN as a negative regulator of HSC proliferation across developmental stages, notwithstanding the impact on their overall stemness. Inhibition of its interaction with ITGAV in the FL led to the expansion of the HSC pool size without any loss of stemness. We investigated if POSTN was involved in the creation of HSC niche in the FL tissue. To determine where POSTN is expressed within the E14.5 FL tissue, immuno-histochemical analysis was performed on 10-μm formalin-fixed paraffin-embedded sections. Confocal imaging clearly showed that POSTN was expressed in most of the skeletal primordial structures linked with bone formation (Figure 7A). Further examination of POSTN expression in specific tissues within the fetal sections revealed high levels of expression in the cartilage primordia of the rib shaft (Figure 7B). In fact, the entire vascular perichondrium surrounding the non-vascular fetal hyaline cartilage expressed POSTN. However, we did not observe any POSTN expression within the developing rib marrow. In addition to the developing ribs, we observed abundant POSTN expression associated with the skeletal muscle fibers of the developing diaphragm that is rich in connective tissue and tendon (Figure 7C). Gross analysis showed that the majority of cells in the FL tissue did not express POSTN (Figure 7A). However, upon detailed examination of the FL tissue, we observed POSTN signals from the vascular lining not restricted to a specific part of the tissue (Figure 7D). Vascular endothelium has been shown to play an important role in creating the HSC niche in the BM as well as in the FL tissue. Therefore, we tested if indeed it was the endothelium associated with the developing hepatic vasculature that expressed POSTN. We used Endoglin and CD31 as endothelial markers and examined the proportion of these cells that expressed POSTN. We analyzed z stack images taken at 0.35-μm step-size to count individual endothelial cells and tested for POSTN expression (Figure S6; Videos S1, S2, S3, and S4). Quantification showed that 91.29% ± 3.07% of Endoglin^{+} (Figure S6A; Video S1 and S2) and 89% ± 2.08% of CD31^{+} cells (Figure S6B; Videos S3 and S4) also expressed POSTN (n = 3, N = 271–300). We detected POSTN expression in some non-endothelial cells also, but vascular endothelium was clearly the most prominent cell type with POSTN expression. Careful observations showed that at least a part of the non-endothelial POSTN signals appeared non-cellular. As POSTN is a known ECM binding protein, we examined if POSTN showed co-localization with laminin associated with basal lamina (Figure 7E; Video S5). It was clear that CD31^{+} endothelial cells expressed POSTN, although both signals were spatially distinct (Figure 7E, lower panel; Video S6). Importantly, POSTN signals showed clear overlap with laminin, associated with basal lamina. Overall, we show that POSTN is a potential component of HSC niche in the FL tissues and acts as a negative regulator of proliferation in otherwise proliferative HSCs.

DISCUSSION

We earlier demonstrated that POSTN-ITGAV interaction promotes quiescence in adult BM HSCs (Khurana et al., 2016). Here, we present evidence that systemic loss of *Postn* results in increased proliferation of FL HSCs. It has been well documented that the HSCs in FL tissue proliferate extensively (Morrison et al., 1995). In addition, upon transplantation in lethally irradiated animals competitive repopulation units (CRUs) from E14.5 FL show more robust engraftment than BM CRUs, along with a much higher clonal expansion (Rebel et al., 1996). Our studies confirm the observation that significantly fewer FL HSCs are quiescent (in the G0 stage of the cell cycle) compared with adult BM HSCs. The fraction of FL HSCs in S and G2M phases was increased in *Vav-Itgav*$^{-/-}$ embryos, perhaps indicating faster G1 progression. Long-term repopulation assays demonstrated that, in contrast to the BM HSCs from *Postn*$^{-/-}$ mice, enhanced proliferation of FL HSCs did not affect their function. Hence, enhanced proliferation of FL HSCs resulted in de facto expansion rather than functional decline. This was reflected clearly in their robust multi-lineage engraftment potential, even upon transplantation into secondary recipients.

It has been demonstrated that HSCs keep proliferating postnatally to create a pool of HSCs that take up newly formed BM niches (Bowie et al., 2006). In mice, transition from a proliferative to quiescent phenotype takes place 3–4 weeks after birth. Detailed experiments later showed that E14.5 FL-derived HSCs, transplanted in irradiated hosts for 4–6 weeks, were indistinguishable from adult

(E) Immuno-staining of the FL sections with endothelial marker CD31 and ECM protein laminin, along with POSTN. The sections were counterstained with Hoechst 33342 (upper panel) (n = 4). Scale bar, 10 μm. The lower panel shows an enlarged part of the section shown in the upper panel. Scale bar, 5 μm).
See also Figure S6.

BM HSCs (Bowie et al., 2007). As the proliferative HSCs from FL transit to a quiescent state after transplantation, the phenotype due to lack of *Itgav* will be due to the changes in functioning of the adult HSCs. Due to intrinsic genetic alteration, true reflection of functional change specifically at fetal stage will be masked. Therefore, a snapshot of the functional status of HSCs after interruption of POSTN-ITGAV interaction was possible only by using *Postn*$^{-/-}$ FL HSCs. It could be seen through the long-term repopulation assays performed using total FL cells that donor-derived chimerism was higher when cells from *Postn*$^{-/-}$ fetuses were transplanted. Further studies using sorted HSCs confirmed that the HSCs from *Postn*$^{-/-}$ FL tissues did not differ in their engraftment potential. Put together, *in vivo* repopulation assays confirmed an overall expansion of HSCs in FL, wherein POSTN-ITGAV axis was disrupted. This was unlike their BM counterparts, where a loss of function upon entry into the cell cycle was observed; indicating intrinsic differences between the stem cell populations from different developmental stages. In fact, the *in vivo* engraftment assays using *Vav-Itgav*$^{-/-}$ FL HSCs confirmed our earlier findings. We observed poorer engraftment, with myeloid skewing, as these cells transited to the adult BM phenotype after transplantation.

Earlier, our group (Manesia et al., 2015) and McKinney-Freeman et al. (2012) reported the transcriptional profile of primitive HSCs from different fetal developmental stages compared with adult BM HSCs. Further evaluation of pathways that were differentially expressed in our RNA-seq dataset identified increased levels of DDR pathway genes in FL HSCs (Figure S5). DNA damage accumulation in HSCs is linked with functional decline during aging (Rossi et al., 2007). In addition, in a number of mouse models with defective DDRs, pre-mature aging of HSCs was observed (Ito et al., 2004). These changes involve increased proliferation of HSCs, poorer engraftment potential, and differentiation skewed toward myelopoiesis at the expense of lymphopoiesis (Rossi et al., 2005). Our results demonstrate that the outcome of increased HSC proliferation is influenced by the developmental context. We observed that *ex vivo* culture of E14.5 FL HSCs was associated with significantly better DNA damage repair than adult HSCs. Not only was there an increased expression of genes belonging to different DDR pathways, but we also observed that the extent of DNA repair was significantly greater in FL than adult BM HSPCs. In line with these findings, we observed increased levels of DNA damage repair genes resulting in lower levels of double-strand breaks in the FL HSCs from *Postn*-deficient embryos. It has clearly been established that DNA damage signaling is well-linked with cell-cycle progression through several check-points (Jackson and Bartek, 2009). Our results indicate that the strength of this signaling might be developmental context dependent. First, a stronger activation of DDR pathways during embryonic development ensures that the proliferation stress is well tolerated. Secondly, a further increase in proliferation rates induced faster repair kinetics in HSCs in *Postn*-deficient embryos resulting in overall HSC expansion without any loss of stemness.

We earlier reported that E14.5 FL HSCs have higher mitochondrial activity resulting in increased levels of reactive oxygen species (ROS) (Manesia et al., 2015). However, this oxidative stress appears to be well tolerated by the fetal HSCs unlike their adult BM counterparts (Wang et al., 2014). The observation that FL HSCs possess enhanced DNA repair capabilities, reported here, may also help explain this contrast. Increased ROS levels are considered to be one of the major factors underlying DNA damage accumulation, ultimately leading to poorly regulated cell-cycle progression (Yahata et al., 2011). Antioxidants have been used to reverse the effects of increased ROS levels in response to increased mitochondrial respiration (Ito et al., 2006).

In addition to providing clues to the genetic basis of functional differences between fetal and adult HSCs, our results also provide key insights into the role of outside-in integrin signaling. The results presented here confirm the role of the POSTN-ITGAV axis in the regulation of cell-cycle progression in HSCs irrespective of the developmental stage. A recent study implicated the POSTN-integrin axis in mediating the effects of the vitamin K antagonist warfarin, used clinically to prevent thromboembolic events (Verma et al., 2019). Warfarin inhibited HSC function through blocking γ-carboxylation of POSTN, decreasing the binding of POSTN to ITGB3. Infusion of carboxylated POSTN rescued animals from warfarin-induced coagulation defects. Expression of ITGB3, an ITGAV binding partner, has been correlated with the quiescent properties and long-term repopulation potential of HSCs (Umemoto et al., 2006). Although, RNA-seq results demonstrate that transcript levels of *Itgav* and *Itgb3* in FL HSCs is lower than in BM HSCs, significant levels of cell surface expression of ITGAV and ITGB3 were observed in E14.5 FL-derived primitive HSCs. There is evidence that the ITGAV-B3 heterodimer can be involved in inside-out signaling, affecting adhesion of cells to ECM, possibly through binding to POSTN (Umemoto et al., 2012). However, we have demonstrated that loss of *Itgav* from adult BM HSCs did not affect their adhesion potential (Khurana et al., 2016).

The availability of modern imaging tools, aided with more accurate reporter mouse lines, contributed to the expansion in our knowledge about the physical location of HSCs in the BM. These tools have unequivocally established the importance of BM vasculature in the physical maintenance of primitive HSCs (Boulais and Frenette, 2015). Even functional decline of HSCs during aging has

been attributed to the genetic and physical changes in the vascular niche (Kusumbe et al., 2016). The most crucial of the factors that regulate HSC function have been shown to be expressed within the cellular components of these niches. A variety of cell types associated with different vessel types have been shown to express crucial regulators of adult hematopoiesis (Sasine et al., 2017). Of note, the expression of hematopoietic regulators, such as SCF (Ding et al., 2012) and stromal cell-derived factor-1α (Ding and Morrison, 2013) was used to identify the microenvironment of the most primitive HSCs in the BM. These experiments established the importance of BM vascular niche, not only to physically host the HSCs, but also in regulating their function. Our experiments also showed that POSTN expression is largely restricted to vascular areas in the FL tissue. In fact, recent findings have indicated that HSCs take up vascular niches in the FL (Tamplin et al., 2015). In Zebrafish, endothelial cells assisted the colonization of caudal hematopoietic tissue by the incoming HSCs and this phenomenon was conserved in mouse. Interestingly, at a later stage in mouse FL tissue, it was noted that the $Ng2^+Nestin^+$ pericytes, associated with portal vessels, were a crucial component of the HSC niche (Khan et al., 2016). Overall though, high levels of POSTN expression were observed in skeletal tissues in the developing fetus, such as in the developing ribs and diaphragm, both tissues rich in muscle cells and ECM proteins (Merrell and Kardon, 2013; Quondamatteo et al., 2002). This was in line with the earlier published findings that POSTN is associated with ECM proteins, such as type I collagen (Norris et al., 2007), fibronectin (Takayama et al., 2006), laminin (Nishiyama et al., 2011), as well as skeletal structures.

In conclusion, we present evidence for differential responses of HSCs to enhanced proliferation, such as caused by disruption of the POSTN-ITGAV axis, depending upon their developmental stage. Loss of POSTN in the HSC niche induces proliferation; however, this is associated with functional decline only in the adult BM. By contrast, an expansion of the HSC pool is observed in $Postn^{-/-}$ FL tissue. We present evidence that fetal HSCs might be less vulnerable to proliferation-induced stress due to increased levels of DNA damage repair.

EXPERIMENTAL PROCEDURES

Animals

The animals were bred at the animal facilities of KU Leuven, Rajiv Gandhi Center for Biotechnology, and IISER Thiruvananthapuram. During the experiments, mice were maintained in isolator cages, fed with autoclaved acidified water and irradiated food ad libitum. All animal experiments were approved by the Institutional Animal Ethics Committees for the respective animal facilities, details can be found in Supplemental Information.

Long-Term Repopulation Assays

A single dose of 3.5 Gy (sub-lethal dose) or 10 Gy (sub-lethal dose) was given to the animals, a day before the test cells were transplanted. Mononuclear cells or the sorted HSCs E14.5 *Vav-Itgav*$^{+/+}$, *Vav-Itgav*$^{+/-}$, and *Vav-Itgav*$^{-/-}$ FL tissue were transplanted along with the competitor cells into lethally irradiated mice. Transplantation of sorted or unsorted FL HSCs from *Postn*$^{+/+}$ and *Postn*$^{-/-}$ embryos was performed into sub-lethally irradiated *Rag2*$^{-/-}\gamma C^{-/-}$ mice, without competitor cells. PB chimerism analysis was performed every 4 weeks after which transplantation into secondary recipients was performed, which was followed for an additional 12 weeks. Further details can be found in Supplemental Information.

Statistical Analysis

All data are represented as mean ± SEM. Normal distribution of data was tested using the Shapiro-Wilk test. The equality of group variance was tested using the Brown-Forsythe test. Comparisons between samples from two groups with normally distributed data with equal variance were made using the unpaired two-tailed Student's t test. The Mann-Whitney test was used for comparing two groups where data were non-normally distributed. For multiple comparisons of the normally distributed data with equal variance, one-way ANOVA was performed followed by the Tukey-Kramer post hoc test. Non-normally distributed data was analyzed by the Friedman test. The chi-square test was used for testing the goodness of fit of the observed ratios among genotypes of embryos to expected Mendelian ratio. Statistical analyses were performed with Microsoft Excel or GraphPad Prism 6. For all analyses, p values ≤ 0.05 were accepted as statistically significant, n represents the number of biological repeats and N represents total number of technical repeats across experiments.

SUPPLEMENTAL INFORMATION

Supplemental Information can be found online at https://doi.org/10.1016/j.stemcr.2020.06.022.

AUTHOR CONTRIBUTIONS

S.K. conceptualized the study, laid out the experimental design, supervised the project, analyzed data, and wrote the manuscript. A.B., I.M.R., and P.C.B. performed the experiments and analyzed the results. A.B. assisted in writing the manuscript, and critically reviewed the drafts. J.M. performed computational analysis of the RNA-seq data and reviewed manuscript. S.S. and V.V. provided technical assistance. R.J.A. and J.H. provided material. A.L.-H. provided material and reviewed the manuscript. C.M.V. provided material support and critically reviewed the manuscript.

ACKNOWLEDGMENTS

This work was supported by the DBT-Wellcome Trust India Alliance Fellowship (IA/I/15/2/502061) awarded to S.K. and intramural funds from Indian Institute of Science Education and Research Thiruvananthapuram (IISER TVM). Institutional animal facility is supported by funds from the Department of Science and Technology, Government of India (under FIST scheme; SR/FST/LS-II/2018/217). A.B. is supported by IISER TVM. I.M.R. is supported by Senior

Research Fellowship from University Grants Commission (UGC), India. P.C.B. received support from INSPIRE fellowship from DST, India. C.M.V. is supported by funds from KU Leuven (IDO/13/016 HSC-Niche) and FWO (G0E0117N). The authors would like to acknowledge Ms. Sarika Mohan S. and Ms. Janet S. for technical assistance with flow cytometry core and animal facility, respectively. The authors thank Prof. Louise Purton and Ms. Sohela Sarkar for critically reviewing the manuscript, and Dr. Ravi Maruthachalam for critical suggestions on statistical analyses.

Received: October 18, 2019
Revised: June 25, 2020
Accepted: June 25, 2020
Published: July 30, 2020

REFERENCES

Arroyo, A.G., Yang, J.T., Rayburn, H., and Hynes, R.O. (1999). Alpha4 integrins regulate the proliferation/differentiation balance of multilineage hematopoietic progenitors in vivo. Immunity *11*, 555–566.

Beerman, I., Seita, J., Inlay, M.A., Weissman, I.L., and Rossi, D.J. (2014). Quiescent hematopoietic stem cells accumulate DNA damage during aging that is repaired upon entry into cell cycle. Cell Stem Cell *15*, 37–50.

Boulais, P.E., and Frenette, P.S. (2015). Making sense of hematopoietic stem cell niches. Blood *125*, 2621–2629.

Bowie, M.B., Kent, D.G., Dykstra, B., McKnight, K.D., McCaffrey, L., Hoodless, P.A., and Eaves, C.J. (2007). Identification of a new intrinsically timed developmental checkpoint that reprograms key hematopoietic stem cell properties. Proc. Natl. Acad. Sci. U S A *104*, 5878–5882.

Bowie, M.B., McKnight, K.D., Kent, D.G., McCaffrey, L., Hoodless, P.A., and Eaves, C.J. (2006). Hematopoietic stem cells proliferate until after birth and show a reversible phase-specific engraftment defect. J. Clin. Invest. *116*, 2808–2816.

Crane, G.M., Jeffery, E., and Morrison, S.J. (2017). Adult haematopoietic stem cell niches. Nat. Rev. Immunol. *17*, 573–590.

de Boer, J., Williams, A., Skavdis, G., Harker, N., Coles, M., Tolaini, M., Norton, T., Williams, K., Roderick, K., Potocnik, A.J., et al. (2003). Transgenic mice with hematopoietic and lymphoid specific expression of Cre. Eur. J. Immunol. *33*, 314–325.

Ding, L., and Morrison, S.J. (2013). Haematopoietic stem cells and early lymphoid progenitors occupy distinct bone marrow niches. Nature *495*, 231–235.

Ding, L., Saunders, T.L., Enikolopov, G., and Morrison, S.J. (2012). Endothelial and perivascular cells maintain haematopoietic stem cells. Nature *481*, 457–462.

Eliceiri, B.P., and Cheresh, D.A. (2001). Adhesion events in angiogenesis. Curr. Opin. Cel. Biol. *13*, 563–568.

Gao, X., Xu, C., Asada, N., and Frenette, P.S. (2018). The hematopoietic stem cell niche: from embryo to adult. Development *145*, dev139691.

Gillan, L., Matei, D., Fishman, D.A., Gerbin, C.S., Karlan, B.Y., and Chang, D.D. (2002). Periostin secreted by epithelial ovarian carcinoma is a ligand for alpha(V)beta(3) and alpha(V)beta(5) integrins and promotes cell motility. Cancer Res. *62*, 5358–5364.

Haas, S., Trumpp, A., and Milsom, M.D. (2018). Causes and consequences of hematopoietic stem cell heterogeneity. Cell Stem Cell *22*, 627–638.

Hoggatt, J., Kfoury, Y., and Scadden, D.T. (2016). Hematopoietic stem cell niche in health and disease. Annu. Rev. Pathol. *11*, 555–581.

Hynes, R.O. (2002). Integrins: bidirectional, allosteric signaling machines. Cell *110*, 673–687.

Ito, K., Hirao, A., Arai, F., Matsuoka, S., Takubo, K., Hamaguchi, I., Nomiyama, K., Hosokawa, K., Sakurada, K., Nakagata, N., et al. (2004). Regulation of oxidative stress by ATM is required for self-renewal of haematopoietic stem cells. Nature *431*, 997–1002.

Ito, K., Hirao, A., Arai, F., Takubo, K., Matsuoka, S., Miyamoto, K., Ohmura, M., Naka, K., Hosokawa, K., Ikeda, Y., et al. (2006). Reactive oxygen species act through p38 MAPK to limit the lifespan of hematopoietic stem cells. Nat. Med. *12*, 446–451.

Jackson, S.P., and Bartek, J. (2009). The DNA-damage response in human biology and disease. Nature *461*, 1071–1078.

Khan, J.A., Mendelson, A., Kunisaki, Y., Birbrair, A., Kou, Y., Arnal-Estape, A., Pinho, S., Ciero, P., Nakahara, F., Ma'ayan, A., et al. (2016). Fetal liver hematopoietic stem cell niches associate with portal vessels. Science *351*, 176–180.

Khurana, S. (2016). The effects of proliferation and DNA damage on hematopoietic stem cell function determine aging. Dev. Dynam. *245*, 739–750.

Khurana, S., Buckley, S., Schouteden, S., Ekker, S., Petryk, A., Delforge, M., Zwijsen, A., and Verfaillie, C.M. (2013a). A novel role of BMP4 in adult hematopoietic stem and progenitor cell homing via Smad independent regulation of integrin-alpha4 expression. Blood *121*, 781–790.

Khurana, S., Margamuljana, L., Joseph, C., Schouteden, S., Buckley, S.M., and Verfaillie, C.M. (2013b). Glypican-3-mediated inhibition of CD26 by TFPI: a novel mechanism in hematopoietic stem cell homing and maintenance. Blood *121*, 2587–2595.

Khurana, S., Schouteden, S., Manesia, J.K., Santamaria-Martinez, A., Huelsken, J., Lacy-Hulbert, A., and Verfaillie, C.M. (2016). Outside-in integrin signalling regulates haematopoietic stem cell function via Periostin-Itgav axis. Nat. Commun. *7*, 13500.

Kiel, M.J., and Morrison, S.J. (2008). Uncertainty in the niches that maintain haematopoietic stem cells. Nat. Rev. Immunol. *8*, 290–301.

Kunisaki, Y., Bruns, I., Scheiermann, C., Ahmed, J., Pinho, S., Zhang, D., Mizoguchi, T., Wei, Q., Lucas, D., Ito, K., et al. (2013). Arteriolar niches maintain haematopoietic stem cell quiescence. Nature *502*, 637–643.

Kusumbe, A.P., Ramasamy, S.K., Itkin, T., Mae, M.A., Langen, U.H., Betsholtz, C., Lapidot, T., and Adams, R.H. (2016). Age-dependent modulation of vascular niches for haematopoietic stem cells. Nature *532*, 380–384.

Lacy-Hulbert, A., Smith, A.M., Tissire, H., Barry, M., Crowley, D., Bronson, R.T., Roes, J.T., Savill, J.S., and Hynes, R.O. (2007). Ulcerative colitis and autoimmunity induced by loss of myeloid alphav integrins. Proc. Natl. Acad. Sci. U S A *104*, 15823–15828.

Manesia, J.K., Xu, Z.F., Broekaert, D., Boon, R., van Vliet, A., Eelen, G., Vanwelden, T., Stegen, S., Van Gastel, N., Pascual-Montano, A., et al. (2015). Highly proliferative primitive fetal liver hematopoietic stem cells are fueled by oxidative metabolic pathways. Stem Cell Res. *15*, 715–721.

McKinney-Freeman, S., Cahan, P., Li, H., Lacadie, S.A., Huang, H.T., Curran, M., Loewer, S., Naveiras, O., Kathrein, K.L., Konantz, M., et al. (2012). The transcriptional landscape of hematopoietic stem cell ontogeny. Cell Stem Cell *11*, 701–714.

Merrell, A.J., and Kardon, G. (2013). Development of the diaphragm—a skeletal muscle essential for mammalian respiration. FEBS J. *280*, 4026–4035.

Mitroulis, I., Chen, L.S., Singh, R.P., Kourtzelis, I., Economopoulou, M., Kajikawa, T., Troullinaki, M., Ziogas, A., Ruppova, K., Hosur, K., et al. (2017). Secreted protein Del-1 regulates myelopoiesis in the hematopoietic stem cell niche. J. Clin. Invest. *127*, 3624–3639.

Mohrin, M., Bourke, E., Alexander, D., Warr, M.R., Barry-Holson, K., Le Beau, M.M., Morrison, C.G., and Passegue, E. (2010). Hematopoietic stem cell quiescence promotes error-prone DNA repair and mutagenesis. Cell Stem Cell *7*, 174–185.

Morrison, S.J., Hemmati, H.D., Wandycz, A.M., and Weissman, I.L. (1995). The purification and characterization of fetal liver hematopoietic stem cells. Proc. Natl. Acad. Sci. U S A. *92*, 10302–10306.

Muller, A.M., Medvinsky, A., Strouboulis, J., Grosveld, F., and Dzierzak, E. (1994). Development of hematopoietic stem cell activity in the mouse embryo. Immunity *1*, 291–301.

Nilsson, S.K., Johnston, H.M., Whitty, G.A., Williams, B., Webb, R.J., Denhardt, D.T., Bertoncello, I., Bendall, L.J., Simmons, P.J., and Haylock, D.N. (2005). Osteopontin, a key component of the hematopoietic stem cell niche and regulator of primitive hematopoietic progenitor cells. Blood *106*, 1232–1239.

Nishiyama, T., Kii, I., Kashima, T.G., Kikuchi, Y., Ohazama, A., Shimazaki, M., Fukayama, M., and Kudo, A. (2011). Delayed re-epithelialization in periostin-deficient mice during cutaneous wound healing. PLoS One *6*, e18410.

Norris, R.A., Damon, B., Mironov, V., Kasyanov, V., Ramamurthi, A., Moreno-Rodriguez, R., Trusk, T., Potts, J.D., Goodwin, R.L., Davis, J., et al. (2007). Periostin regulates collagen fibrillogenesis and the biomechanical properties of connective tissues. J. Cell Biochem. *101*, 695–711.

Penta, K., Varner, J.A., Liaw, L., Hidai, C., Schatzman, R., and Quertermous, T. (1999). Del1 induces integrin signaling and angiogenesis by ligation of alphaVbeta3. J. Biol. Chem. *274*, 11101–11109.

Quondamatteo, F., Reinhardt, D.P., Charbonneau, N.L., Pophal, G., Sakai, L.Y., and Herken, R. (2002). Fibrillin-1 and fibrillin-2 in human embryonic and early fetal development. Matrix Biol. *21*, 637–646.

Rebel, V.I., Miller, C.L., Eaves, C.J., and Lansdorp, P.M. (1996). The repopulation potential of fetal liver hematopoietic stem cells in mice exceeds that of their liver adult bone marrow counterparts. Blood *87*, 3500–3507.

Rossi, D.J., Bryder, D., Seita, J., Nussenzweig, A., Hoeijmakers, J., and Weissman, I.L. (2007). Deficiencies in DNA damage repair limit the function of haematopoietic stem cells with age. Nature *447*, 725–729.

Rossi, D.J., Bryder, D., Zahn, J.M., Ahlenius, H., Sonu, R., Wagers, A.J., and Weissman, I.L. (2005). Cell intrinsic alterations underlie hematopoietic stem cell aging. Proc. Natl. Acad. Sci. U S A *102*, 9194–9199.

Sasine, J.P., Yeo, K.T., and Chute, J.P. (2017). Concise review: paracrine functions of vascular niche cells in regulating hematopoietic stem cell fate. Stem Cell Transl. Med. *6*, 482–489.

Savill, J., Dransfield, I., Hogg, N., and Haslett, C. (1990). Vitronectin receptor-mediated phagocytosis of cells undergoing apoptosis. Nature *343*, 170–173.

Sitnicka, E., Lin, N., Priestley, G.V., Fox, N., Broudy, V.C., Wolf, N.S., and Kaushansky, K. (1996). The effect of thrombopoietin on the proliferation and differentiation of murine hematopoietic stem cells. Blood *87*, 4998–5005.

Stier, S., Ko, Y., Forkert, R., Lutz, C., Neuhaus, T., Grunewald, E., Cheng, T., Dombkowski, D., Calvi, L.M., Rittling, S.R., et al. (2005). Osteopontin is a hematopoietic stem cell niche component that negatively regulates stem cell pool size. J. Exp. Med. *201*, 1781–1791.

Takayama, G., Arima, K., Kanaji, T., Toda, S., Tanaka, H., Shoji, S., McKenzie, A.N., Nagai, H., Hotokebuchi, T., and Izuhara, K. (2006). Periostin: a novel component of subepithelial fibrosis of bronchial asthma downstream of IL-4 and IL-13 signals. J. Allergy Clin. Immunol. *118*, 98–104.

Tamplin, O.J., Durand, E.M., Carr, L.A., Childs, S.J., Hagedorn, E.J., Li, P., Yzaguirre, A.D., Speck, N.A., and Zon, L.I. (2015). Hematopoietic stem cell arrival triggers dynamic remodeling of the perivascular niche. Cell *160*, 241–252.

Umemoto, T., Matsuzaki, Y., Shiratsuchi, Y., Hashimoto, M., Yoshimoto, T., Nakamura-Ishizu, A., Petrich, B., Yamato, M., and Suda, T. (2017). Integrin alpha v beta 3 enhances the suppressive effect of interferon-gamma on hematopoietic stem cells. EMBO J. *36*, 2390–2403.

Umemoto, T., Yamato, M., Ishihara, J., Shiratsuchi, Y., Utsumi, M., Morita, Y., Tsukui, H., Terasawa, M., Shibata, T., Nishida, K., et al. (2012). Integrin-alpha v beta 3 regulates thrombopoietin-mediated maintenance of hematopoietic stem cells. Blood *119*, 83–94.

Umemoto, T., Yamato, M., Shiratsuchi, Y., Terasawa, M., Yang, J., Nishida, K., Kobayashi, Y., and Okano, T. (2006). Expression of integrin beta3 is correlated to the properties of quiescent hemopoietic stem cells possessing the side population phenotype. J. Immunol. *177*, 7733–7739.

Umemoto, T., Yamato, M., Shiratsuchi, Y., Terasawa, M., Yang, J., Nishida, K., Kobayashi, Y., and Okano, T. (2008). CD61 enriches long-term repopulating hematopoietic stem cells. Biochem. Biophys. Res. Commun. *365*, 176–182.

Urtasun, R., Lopategi, A., George, J., Leung, T.M., Lu, Y.K., Wang, X.D., Ge, X.D., Fiel, M.I., and Nieto, N. (2012). Osteopontin, an oxidant stress sensitive cytokine, up-regulates collagen-I via integrin aV ss 3 engagement and PI3K/pAkt/NF?B signaling. Hepatology *55*, 594–608.

Verma, D., Kumar, R., R, S.P., Karantanou, C., Zanetti, C., Minciacchi, V.R., Fulzele, K., Kunz, K., Hoelper, S., Zia-Chahabi, S., et al.

(2019). Vitamin K-antagonism impairs the bone marrow microenvironment and hematopoiesis. Blood *134*, 227–238.

Walter, D., Lier, A., Geiselhart, A., Thalheimer, F.B., Huntscha, S., Sobotta, M.C., Moehrle, B., Brocks, D., Bayindir, I., Kaschutnig, P., et al. (2015). Exit from dormancy provokes DNA-damage-induced attrition in haematopoietic stem cells. Nature *520*, 549–552.

Wang, Y.H., Israelsen, W.J., Lee, D., Yu, V.W.C., Jeanson, N.T., Clish, C.B., Cantley, L.C., Vander Heiden, M.G., and Scadden, D.T. (2014). Cell-state-specific metabolic dependency in hematopoiesis and leukemogenesis. Cell *158*, 1309–1323.

Yahata, T., Takanashi, T., Muguruma, Y., Ibrahim, A.A., Matsuzawa, H., Uno, T., Sheng, Y., Onizuka, M., Ito, M., Kato, S., et al. (2011). Accumulation of oxidative DNA damage restricts the self-renewal capacity of human hematopoietic stem cells. Blood *118*, 2941–2950.

Zhang, C.C., and Lodish, H.F. (2005). Murine hematopoietic stem cells change their surface phenotype during ex vivo expansion. Blood *105*, 4314–4320.

Stem Cell Reports, Volume *15*

Supplemental Information

The Periostin/Integrin-αv Axis Regulates the Size of Hematopoietic Stem Cell Pool in the Fetal Liver

Atreyi Biswas, Irene M. Roy, Prathibha C. Babu, Javed Manesia, Sarah Schouteden, Vinod Vijayakurup, Ruby John Anto, Joerg Huelsken, Adam Lacy-Hulbert, Catherine M. Verfaillie, and Satish Khurana

Figure S1

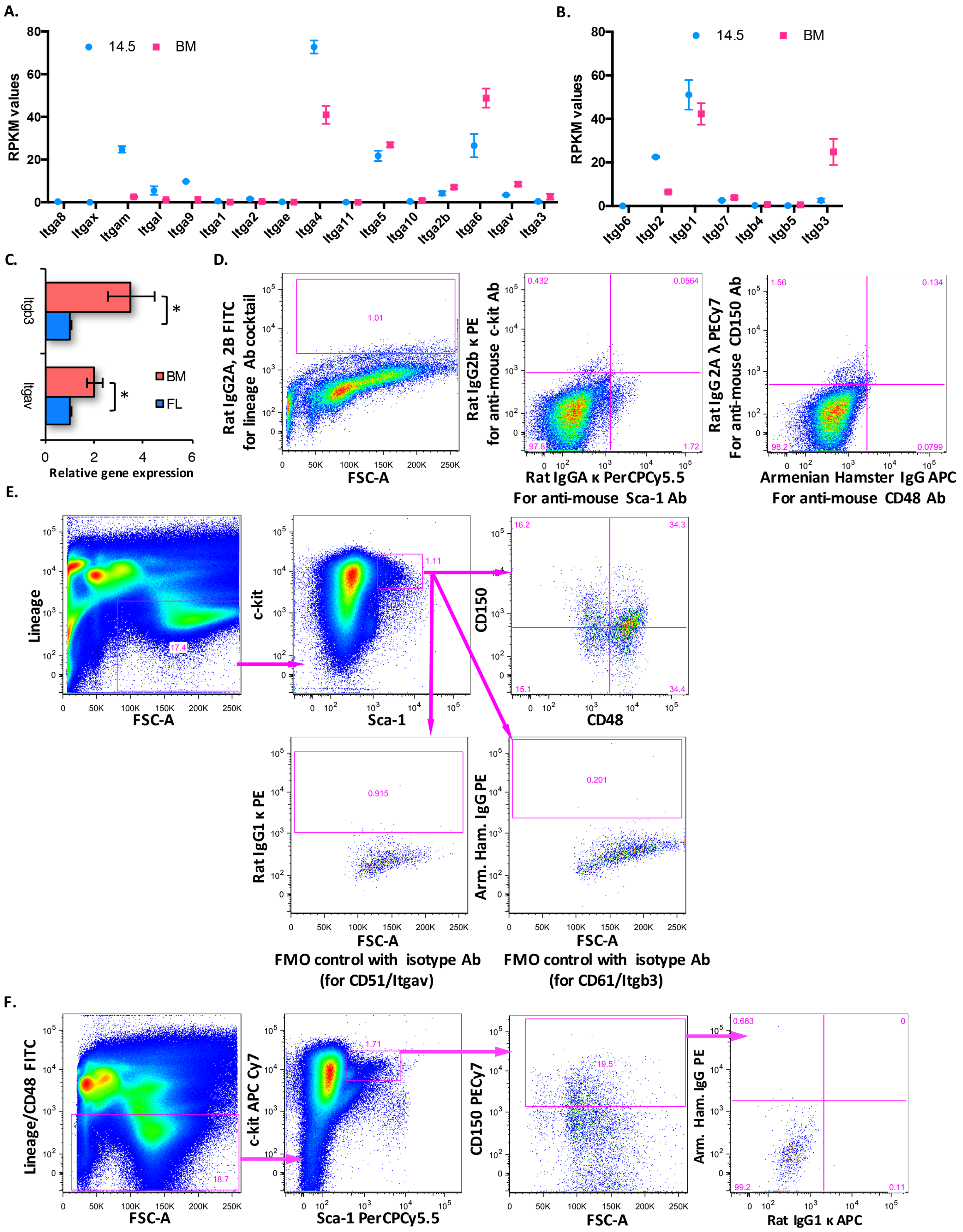

A.
RPKM values
14.5
BM
Itga8
Itgax
Itgam
Itgal
Itga9
Itga1
Itga2
Itgae
Itga4
Itga11
Itga5
Itga10
Itga2b
Itga6
Itgav
Itga3
B.
Itgb6
Itgb2
Itgb1
Itgb7
Itgb4
Itgb5
Itgb3
C.
Itgb3
Itgav
BM
FL
Relative gene expression
D.
Rat IgG2A, 2B FITC for lineage Ab cocktail
FSC-A
Rat IgG2b κ PE for anti-mouse c-kit Ab
Rat IgGA κ PerCPCy5.5
For anti-mouse Sca-1 Ab
Rat IgG 2A λ PECy7 For anti-mouse CD150 Ab
Armenian Hamster IgG APC
For anti-mouse CD48 Ab
E.
Lineage
c-kit
Sca-1
CD150
CD48
Rat IgG1 κ PE
Arm. Ham. IgG PE
FMO control with isotype Ab
(for CD51/Itgav)
FMO control with isotype Ab
(for CD61/Itgb3)
F.
Lineage/CD48 FITC
c-kit APC Cy7
Sca-1 PerCPCy5.5
CD150 PECy7
Arm. Ham. IgG PE
Rat IgG1 κ APC

Figure S1: Expression of Itgav and Itgb3 in FL HSCs.

(A,B) Expression of all known α- (A) and β- (B) integrin chains analyzed by RNASeq of primitive HSCs from E14.5 FL and adult BM. $CD150^{+}CD48^{-}$ LSK cells were sorted out from the two stages to perform paired end sequencing, reported in an earlier study from our group. RPKM values for each gene is presented.

(C) Freshly sorted primitive HSCs ($CD150^{+}CD48^{-}$LSK cells) were used to assess the expression of *Itgav* and *Itgb3* by performing quantitative RT-PCR (Unpaired two tailed Student's t-test was performed. n=6, * $p<0.05$).

(D) Isotype antibody controls for the antibodies used for flow cytometry analysis of different stem cell sub-populations in hematopoietic system. Isotype controls used were FITC conjugated Rat IgG2a and 2b for lineage cocktail, PE/APC-Cy7 conjugated Rat IgG2bκ for c-kit, PerCP-Cy5.5 conjugated Rat IgG2a κ for Sca-1, FITC/APC conjugated Armenian hamster IgG for CD48, PE-Cy7 conjugated Rat IgG2a λ for CD150.

(E) Gating strategy and isotype/FMO controls to examine Itgav and Itgb3 expression in various stem cell sub-populations in E14.5 FL cells. Total mononuclear cells (MNCs) wee labeled with FITC conjugated lineage antibody cocktail, APC conjugated c-kit, PerCP-Cy5.5 conjugated Sca-1, APCCy7 conjugated CD48 and PE-Cy7 conjugated CD150 antibodies. Along with these Isotype antibodies for Itgav (CD51) or Itgb3 (CD61) were also used, separately. These were PE conjugated Rat IgG1κ for CD51 and PE conjugated Armenian hamster IgG was used for CD61.

(F) Fluorescence minus one (FMO) control used for co-expression analysis of Itgav and Itgb3 on primitive HSCs derived from E14.5 FL MNCs.

Related to Figure 1

Figure S2

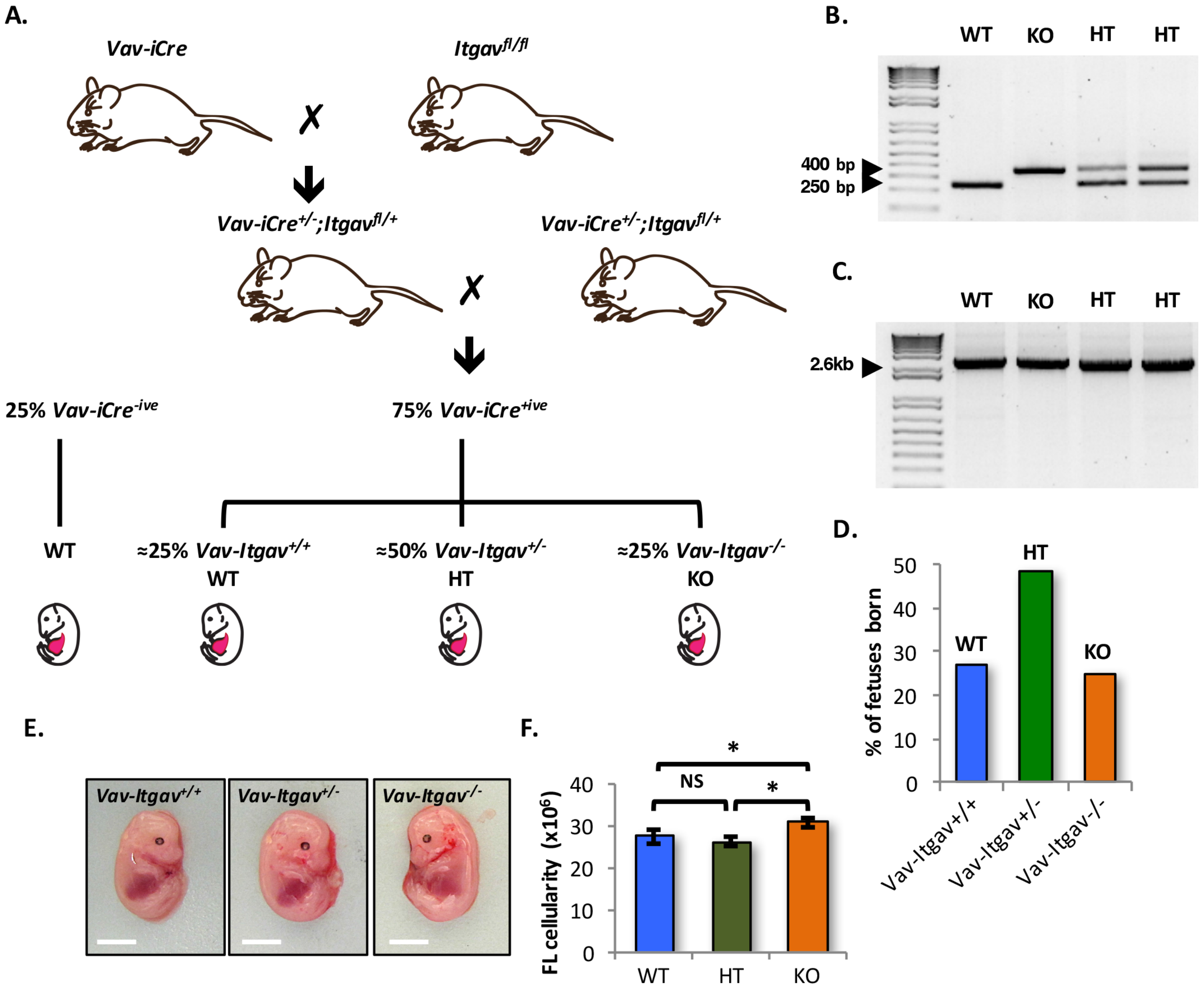

A.
Vav-iCre
Itgavfl/fl
Vav-iCre+/-;Itgavfl/+
Vav-iCre+/-;Itgavfl/+
25% Vav-iCre-ive
75% Vav-iCre+ive
WT
≈25% Vav-Itgav+/+
WT
≈50% Vav-Itgav+/-
HT
≈25% Vav-Itgav-/-
KO
B.
WT
KO
HT
HT
400 bp
250 bp
C.
WT
KO
HT
HT
2.6kb
D.
% of fetuses born
50
40
30
20
10
0
WT
HT
KO
Vav-Itgav+/+
Vav-Itgav+/-
Vav-Itgav-/-
E.
Vav-Itgav+/+
Vav-Itgav+/-
Vav-Itgav-/-
F.
FL cellularity (x10^6)
40
30
20
10
0
NS
*
*
WT
HT
KO

Figure S2: Generation and preliminary characterization of *Vav-Itgav*$^{-/-}$ fetuses

A) *Vav-iCre* and *Itgav*$^{fl/fl}$ mice were crossed to conditionally delete *Itgav* in hematopoietic system. Fetuses at E14.5 from F2 generation were harvested and genotyped to identify *Vav-Itgav*$^{+/+}$ (WT), *Vav-Itgav*$^{+/-}$ (HT), *Vav-Itgav*$^{-/-}$ (cKO) embryos.

B,C) Genotyping PCR was performed to identify *Itgav*$^{+}$/*Itgav*^{fl} alleles as well as *Vav-iCre* construct using specific primers given in Table S1.

D) Comparison between the numbers of *Vav-iCre*$^{+}$ fetuses from each genotype *Vav-Itgav*$^{+/+}$, *Vav-Itgav*$^{+/-}$, *Vav-Itgav*$^{-/-}$. To test the goodness of fit of the observed ratios among genotypes of embryos to expected mendelian ratio, Chi-square test was employed (n=15-29, $\chi^2_{df=2}$=0.049, p=0.98).

E) Gross morphology of the fetuses upon mono- or bi-allelic deletion of *Itgav* in comparison to control littermates. Scale bar=5mm

F) Comparison of the total number of mononuclear cells from fetal liver tissue per embryo. (n=3, N=8-12, t test: * p<0.05)

Related to Figure 2

Figure S3

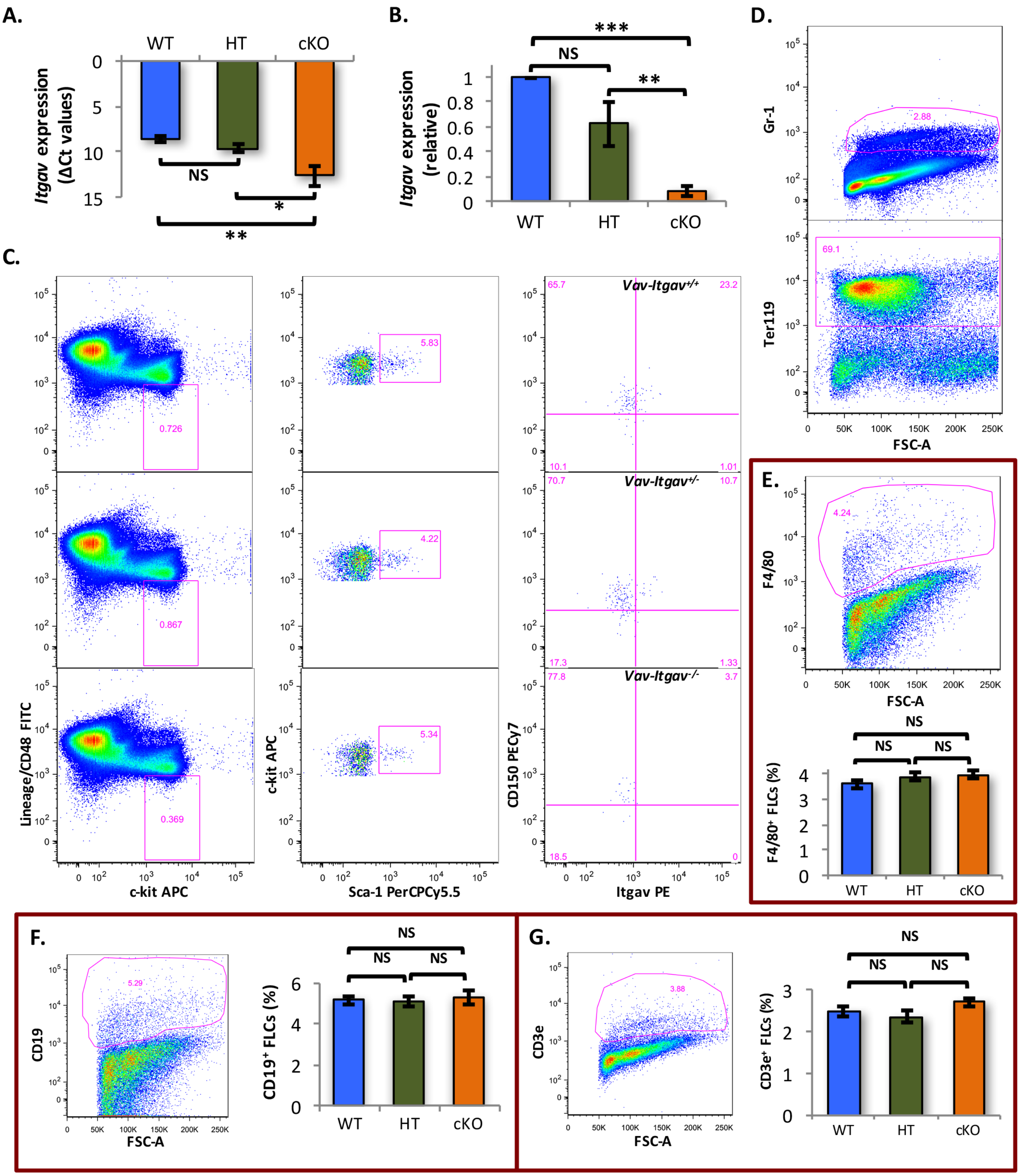
A.
Itgav expression (ΔCt values)
WT
HT
cKO
NS
*
**
B.
Itgav expression (relative)
***
NS
**
C.
Vav-Itgav+/+
Vav-Itgav+/-
Vav-Itgav-/-
Lineage/CD48 FITC
c-kit APC
Sca-1 PerCPCy5.5
CD150 PECy7
Itgav PE
D.
Gr-1
Ter119
FSC-A
E.
F4/80
F4/80+ FLCs (%)
F.
CD19
CD19+ FLCs (%)
G.
CD3e
CD3e+ FLCs (%)

Figure S3: Confirmation of Itgav deletion and lineage analysis of E14.5 *Postn*$^{-/-}$ fetal liver tissues

A,B) Confirmation of *Vav-iCre* mediated *Itgav*-deletion in hematopoietic stem and progenitor cells. LSK cells from FL tissues were sorted from *Vav-Itgav*$^{+/+}$ (WT), *Vav-Itgav*$^{+/-}$ (HT), *Vav-Itgav*$^{-/-}$ (cKO) embryos. Freshly isolated cells were used for quantification of *Itgav* expression using quantitative RT-PCR analysis. Expression levels were shown using ΔCt values (A) and comparisons were made by calculating fold changes following mono- or bi-allelic deletion of *Itgav* (B).

(C) Flowcytometry based assessment of Itgav expression in primitive HSCs from *Vav-Itgav*$^{+/+}$ (WT), *Vav-Itgav*$^{+/-}$ (HT), *Vav-Itgav*$^{-/-}$ (KO) embryos.

D-G) Flow cytometry based analysis performed to compare the frequency of various lineage-committed cells in E14.5 FL tissue from WT, HT and cKO embryos. (D) Gating strategies followed for analyzing Gr-1^{+} and Ter119^{+} populations. (E-G) Flow cytometry based analysis performed to compare the frequency of F4/80+ (E), CD19+ (F) and CD3e+ cells (G) in E14.5 FL tissue from WT, HT and cKO embryos. Gating strategies are shown in upper panels.

(n=8-12, t-test NS $p<0.05$)

Related to Figure 2

Figure S4

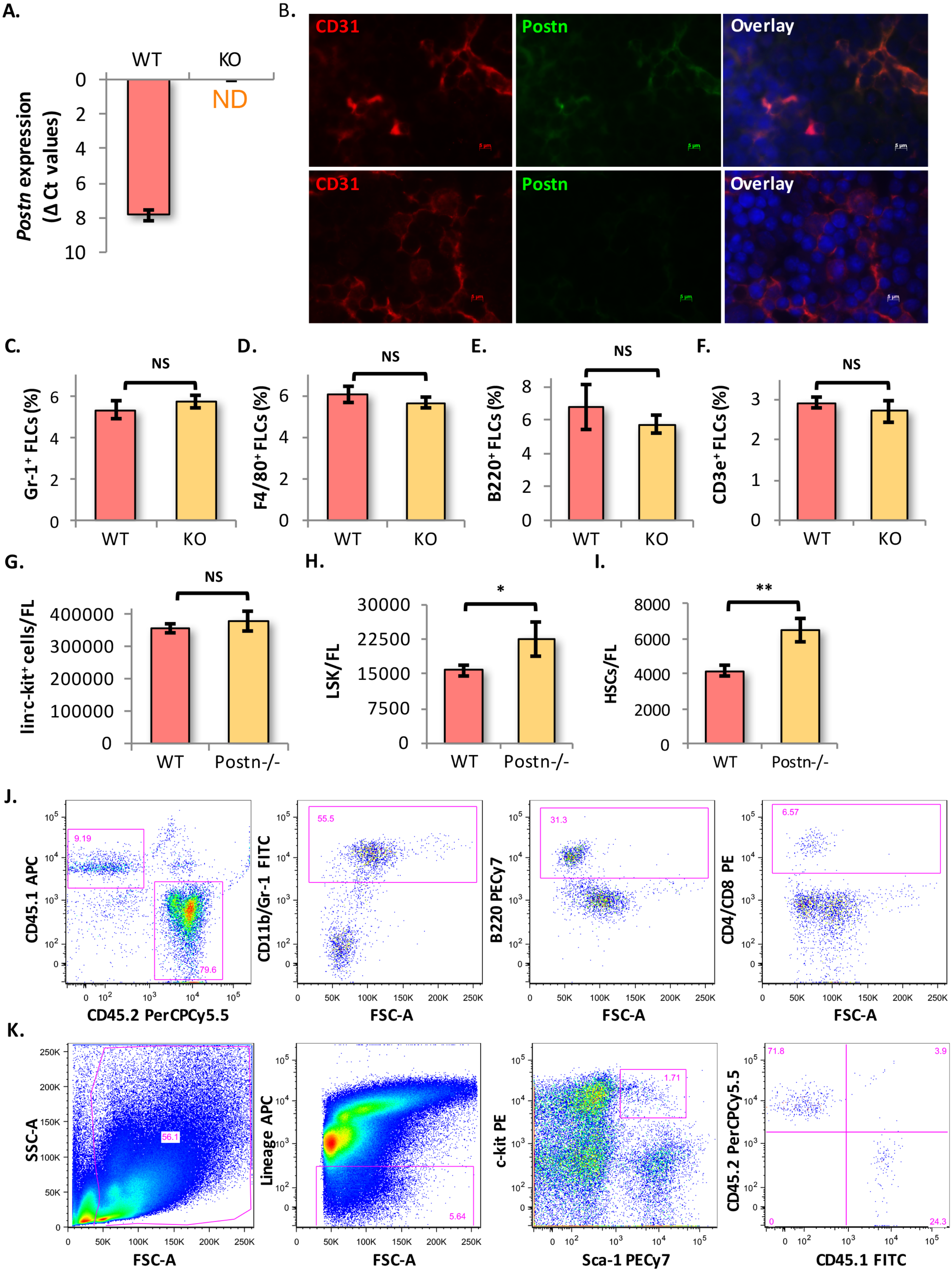
A.
WT
KO
ND
Postn expression (Δ Ct values)
B.
CD31
Postn
Overlay
C.
NS
Gr-1+ FLCs (%)
D.
F4/80+ FLCs (%)
E.
B220+ FLCs (%)
F.
CD3e+ FLCs (%)
G.
lin-c-kit+ cells/FL
Postn-/-
H.
*
LSK/FL
I.
**
HSCs/FL
J.
CD45.1 APC
CD45.2 PerCPCy5.5
CD11b/Gr-1 FITC
B220 PECy7
CD4/CD8 PE
FSC-A
K.
SSC-A
Lineage APC
c-kit PE
Sca-1 PECy7
CD45.2 PerCPCy5.5
CD45.1 FITC

Figure S4: Lineage analysis of E14.5 *Postn*$^{-/-}$ fetal liver tissues

A) Confirmation of lack of *Postn* expression in hematopoietic niche in the E14.5 fetal liver. Quantitative RT-PCR was performed on lin$^-$CD45$^-$ cells isolated from the FL tissues by MACS.

B) Postn specific antibodies were used to detect its expression in WT FL tissue at E14.5 (upper panel). Vasculature was identified by using anti-CD31 antibodies. Nuclear counterstaining was performed using Hoechst 33342. Lack of expression of Postn in FL tissues from *Postn*$^{-/-}$ embryos was also confirmed (lower panel). Scale bar=5µm

(C-F) Mononuclear cells from E14.5 fetal liver cells were analyzed for the proportion of; C) Gr-1$^+$ (granulocytes), D) F4/80$^+$ (macrophages), E) B220$^+$ cells (B-cells) and F) CD3e$^+$ cells (T-cells). (n=11-14, t-test NS $p<0.05$)

(G-I) Comparison of the total number of hematopoietic stem and progenitor cells per FL from WT, Postn$^{-/-}$ embryos at E14.5; (G) lin$^-$c-kit$^+$ cells, (H) LSK cells, (I) primitive HSCs.

J) Gating strategy used to quantify donor-derived chimerism. CD45.1$^+$ cells were identified as donor-derived cells, while CD45.2$^+$ cells were recognized as recipients' leukocytes. Multi-lineage chimerism into myeloid (CD11b/Gr-1$^+$ cells), B-cell (B220$^+$) and T-cell lineage (CD4/CD8$^+$) was examined within the donor-derived population in animals transplanted with FL cells or freshly isolated FL HSCs from WT/KO embryos.

K) Gating strategy followed to quantify donor-derived chimerism in BM LSK cell population. LSK cells was gated from within the total BM MNC population and the proportion of donor-derived cells was examined by using antibodies against CD45.1 and CD45.2.

An unpaired two tailed Student's t-test was performed. n=3, N=15-18, t test: * $p<0.03$, **$p\leq0.01$, NS indicates not significant.

Related to Figure 4

Figure S5

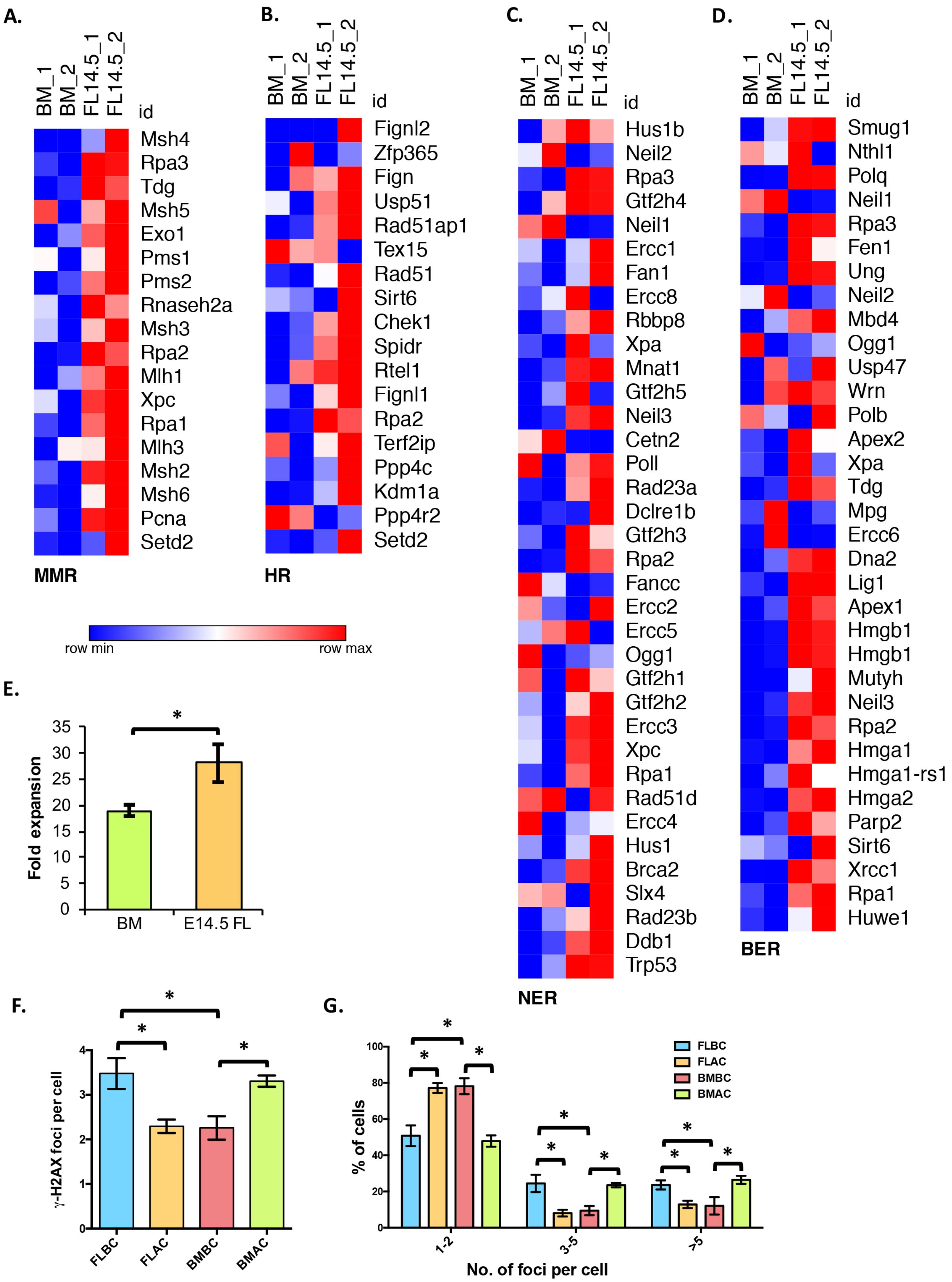

A.
BM_1
BM_2
FL14.5_1
FL14.5_2
id
Msh4
Rpa3
Tdg
Msh5
Exo1
Pms1
Pms2
Rnaseh2a
Msh3
Rpa2
Mlh1
Xpc
Rpa1
Mlh3
Msh2
Msh6
Pcna
Setd2
MMR
B.
Fignl2
Zfp365
Fign
Usp51
Rad51ap1
Tex15
Rad51
Sirt6
Chek1
Spidr
Rtel1
Fignl1
Rpa2
Terf2ip
Ppp4c
Kdm1a
Ppp4r2
Setd2
HR
row min
row max
C.
Hus1b
Neil2
Rpa3
Gtf2h4
Neil1
Ercc1
Fan1
Ercc8
Rbbp8
Xpa
Mnat1
Gtf2h5
Neil3
Cetn2
Poll
Rad23a
Dclre1b
Gtf2h3
Rpa2
Fancc
Ercc2
Ercc5
Ogg1
Gtf2h1
Gtf2h2
Ercc3
Xpc
Rpa1
Rad51d
Ercc4
Hus1
Brca2
Slx4
Rad23b
Ddb1
Trp53
NER
D.
Smug1
Nthl1
Polq
Neil1
Rpa3
Fen1
Ung
Neil2
Mbd4
Ogg1
Usp47
Wrn
Polb
Apex2
Xpa
Tdg
Mpg
Ercc6
Dna2
Lig1
Apex1
Hmgb1
Hmgb1
Mutyh
Neil3
Rpa2
Hmga1
Hmga1-rs1
Hmga2
Parp2
Sirt6
Xrcc1
Rpa1
Huwe1
BER
E.
Fold expansion
35
30
25
20
15
10
5
0
BM
E14.5 FL
*
F.
γ-H2AX foci per cell
4
3
2
1
0
FLBC
FLAC
BMBC
BMAC
*
G.
% of cells
100
80
60
40
20
0
1-2
3-5
>5
No. of foci per cell
FLBC
FLAC
BMBC
BMAC
*

Figure S5: DNA damage response pathway genes are enriched in E14.5 FL derived LT-HSCs

A-D) Heatmap showing relative transcript levels of genes from DNA damage response pathways. RPKM values were used from RNA-Seq analysis of FACS sorted HSCs from E14.5 FL and adult BM HSCs (accession number E-MTAB-4034).

A) mismatch repair (MMR), **B)** homologous recombination (HR), **C)** nucleotide excision repair (NER), and **D)** base excision repair (BER) differentially regulated in FL versus BM derived LT-HSCs identified earlier through KEGG pathway analysis.

E-G) Bone marrow and E14.5 FL derived LSK cells were cultured in serum free medium in the presence of SCF and Tpo for five days. Freshly cells were counted and immunostaining for γ-H2AX was performed on freshly isolated (before culture; BC) and cultured LSK (after culture; AC) cells from E14.5 FL and adult BM.

E) Comparison of the expansion potential of BM and E14.5 FL derived LSKs. The ratio of cells harvested after 5 days of culture with starting population was compared between the two developmental stages.

F) Quantification of γ-H2AX foci per cell in freshly isolated or cultured FL and BM derived LSK cells.

G) Comparison of proportion of cells with different levels of DNA damage based on number of γ-H2AX foci per cell.

n=4, N>173, one-way ANOVA followed by Tukey's multiple comparisons test * $p<0.05$.

Related to Figure 5 and Figure 6

Figure S6

A.

B.

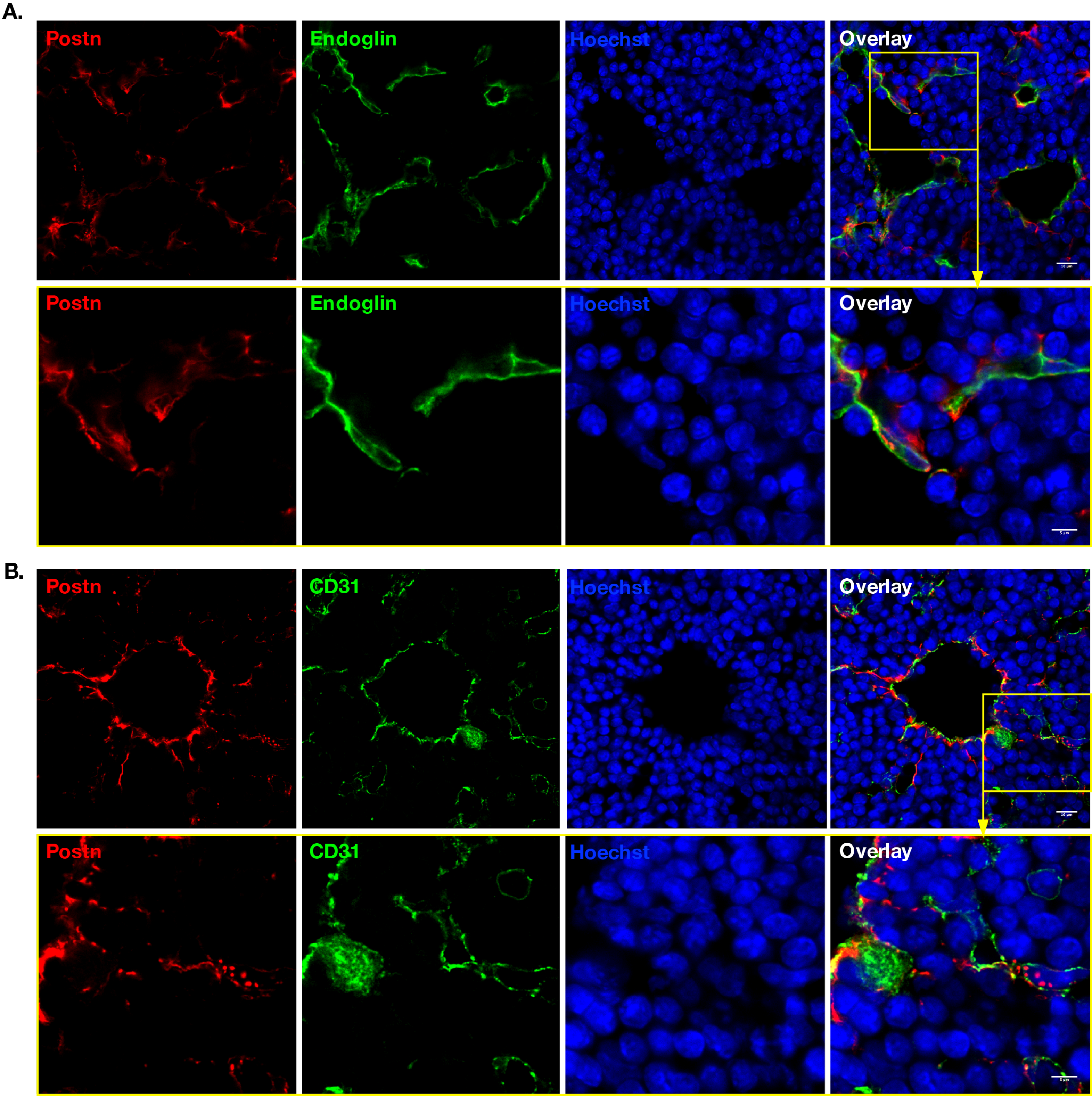

Postn
Endoglin
Hoechst
Overlay
Postn
Endoglin
Hoechst
Overlay
Postn
CD31
Hoechst
Overlay
Postn
CD31
Hoechst
Overlay

Figure S6: Postn is expressed in vascular endothelium of the fetal liver

Immuno-staining performed to identify the cells that express Postn. Vascular endothelial cells were identified by using specific markers; (A) Endoglin and (B) CD31. Immunostaining was performed using Postn specific antibodies along with the endothelial cell specific markers. Lower panels for both A and B show enlarged portion of the sections shown in the upper panel. Counterstaining for nucleus was performed using Hoechst 33342 (n=3-5, scale bar= 10µm for upper panels and 5µm for the lower panels)

Related to Figure 7

Table S1. Primer sets used in this study, related to Figure 5, 6, S1, S2, S3 and S4

S.No.	**Primer Name**	**Sequence 5'-3'**
1	Genotyping Postn-/-_F	GGT GCT TCT GTA AGG CCA TC
	Genotyping Postn-/-_R	GTG AGC CAG GAC CTT GTC ATA
	Genotyping Postn-/-_Int-as	AGC ACT GAC TGC GTT AGC AA
2	Genotyping Itgav-fl/fl_F	GGTGACTCAATCTGTGACCTTCAGC
	Genotyping Itgav-fl/fl mice_R	CACAAATCAAGGATGACCAAACTGAG
3	Genotyping Vav-iCre_F	CCATGGCACCCAAGAAGAAG
	Genotyping Vav-iCre_R	GCTTAGTTTTCCTGCAGCGG
4	Mm-Topbp1_F	AGAGGCTACTGCCCAGAACA
	Mm-Topbp1_R	CGAGGCCGTTTGACTACATT
5	Mm-Brca1_F	GGCTTGACCCCCAAAGAAGT
	Mm-Brca1_R	TGTCCGCTCACACACAAACT
6	Mm-Palb2_F	GGGAAACGAAAATCAGCCCG
	Mm-Palb2_R	AACCACGCCTCTGTTCTGAC
7	Mm-Mre11a_F	CTGGGAGCGGTTTTCTTGTG
	Mm-Mre11a_R	TGGATCTGTGGGGCTCATTT
8	Mm-Blm_F	CTTGGGAGCTGAAAGAGGTG
	Mm-Blm_R	AACGAGGAAGAAGCAGTGGA
9	Mm-Pcna_F	GAGGGTTGGTAGTTGTCGCT
	Mm-Pcna_R	CTCAAACATGGTGGCGGAGT
10	Mm-Rfc4_F	GCCAAAGCACAACTGACCAAG
	Mm-Rfc4_R	CACTGCAACCACTTCGTCCT
11	Mm-Rfc5_F	AGAACGCCTTGAGACGAGTG
	Mm-Rfc5_R	TCAGAGGGCCAAATCGGAAC
12	Mm-Rpa3_F	CGCCAGCATGTTACCACAGTA
	Mm-Rpa3_R	ATTTCCTCGTCAAGTGGCTCC
13	Mm-Ddb1_F	GTGTCTCAAGAGCCCAAAGC
	Mm-Ddb1_R	TCTCTGTGTGGCTGATTTGC
14	Mm-Cetn2_F	TGCAGTGGCTTCTTAGTTGTCC
	Mm-Cetn2_R	ATGCCACAGCAAGCACTCAT
15	Mm-Neil1_F	AAGGGGCTGGTATTTGGTGG
	Mm-Neil1_R	CTCAATGTCAAGCGCAGCTC
16	Mm-Neil3_F	CGGTGGAAAGCCAACAGAGA
	Mm-Neil3_R	ACACATCACACAGCATCCGA
17	Mm-Xrcc1_F	AAAGAGTGGGTGCTGGACTG
	Mm-Xrcc1_R	AGCTTGGGAGCTTCGTCTTC
18	Mm-Tdg_F	CCCCGATCCTGTGCTATTCTC
	Mm-Tdg_R	GTCACGGTTGCCATGTTAGG
19	Mm-Exo1_F	CCTACAGCCACTTAGACCCAA
	Mm-Exo1_R	ACAATCTTAGGCAGACAGCTCC
20	Mm-Setd2_F	CCCGACCCCTGAAGAAGAAG
	Mm-Setd2_R	CCGTCCCTGTTCCTCCAAAT
21	Mm-Ercc1_F	AAGAACTTCGCCCTTCGTGT
	Mm-Ercc1_R	GCTTCCTCTGCACTCCAGG
22	Mm-Rad51_F	TCTGTAAGTGGGAATGGGTG
	Mm-Rad51_R	TGCTGCATGTAAGACTCCTT
23	Mm-Postn_F	TGCTGCCCTGGCTATATGAG
	Mm-Postn_R	GTAGTGGCTCCCACAATGCC
24	Mm-Itgav_F	CCAGCCCATTGAGTTTGATT
	Mm-Itgav_R	TTTGACCTGCATGGAGCATA

Table S2. List of antibodies used in this study, related to Figure 1, 2, 3, 4, 5, 7, S1, S3, S4, S5, S6.

Sr. No.	Antibody	Company	Catalogue No.	Clone	Isotype
1	anti-mouse B220 FITC	BD Biosciences	553088	RA36B2	Rat IgG2a, κ
2	anti-mouse Ter119 FITC	Ebiosciences	11-5921-85	TER119	Rat IgG2b, κ
3	anti-mouse Gr1 FITC	Ebiosciences	11-5931-85	RB6-8C5	Rat IgG2b, κ
4	anti-mouse CD4 FITC	Tonbo	35-0041	GK1.5	Rat IgG2b, κ
5	anti-mouse CD8a FITC	Tonbo	35-0081	53-6.5	Rat IgG2a, κ
6	anti-mouse c-kit APC	Tonbo	20-1172	ACK2	Rat IgG2b, κ
7	anti-mouse c-kit APCCy7	Biolegend	105826	2B8	Rat IgG2b, κ
8	anti-mouse Sca-1 PerCPCy5.5	Biolegend	122524	D7	Rat IgG2a, κ
9	anti-mouse CD150 PECy7	Ebiosciences	25-1502	mShad150	Rat IgG2a, λ
10	anti-mouse CD48 APCCy7	Biolegend	103432	HM48-1	Armenian Hamster IgG
11	anti-mouse CD48 FITC	Ebiosciences	11-0481-85	HM48-1	Armenian Hamster IgG
12	anti-mouse CD51 Biotin	Ebiosciences	13-0512-82	RMV-7	Rat IgG1, κ
13	anti-mouse CD51 PE	LS Bio	Ls-C106287	RMV-7	Rat IgG1, κ
14	anti-mouse CD61 PE	Ebiosciences	12-0611-83	2C9.G3	Armenian Hamster IgG
15	anti-mouse CD45.1 APC	BD Biosciences	558701	A20	Mouse IgG2a, κ
16	anti-mouse CD45.2 PerCPCy5.5	BD Biosciences	552950	104	Mouse IgG2a, κ
17	anti-mouse F4/80 APCCy7	Biolegend	123117	BM8	Rat IgG2a, κ
18	anti-mouse CD3e	BD Biosciences	553064	145-2C11	Armenian Hamster IgG1 κ
19	anti-mouse Postn	R&D	AF2955	Polyclonal	Goat IgG
20	anti-mouse CD31	R&D	AF3628	Polyclonal	Goat IgG
21	anti-mouse endoglin	eBioscience	13-1051-82	MJ7/18	Rat IgG2a, κ
22	anti-mouse laminin	abcam	ab11575	Polyclonal	Rabbit IgG
23	anti-mouse γ-H2AX AF647	BD	560447	N1-431	Mouse BALB/c IgG1, κ
24	anti-mouse CD11b FITC	Ebiosciences	11-0112-82	M1/70	Rat IgG2b, κ
25	anti-mouse B220 PECy7	Ebiosciences	25-0452-82	RA3-6B2	Rat IgG2a, κ
26	anti-mouse CD4 PE	Tonbo	50-0041-U100	GK1.5	Rat IgG2b, κ
27	anti-mouse CD8 PE	eBioscience	12-0081-82	53-6.7	Rat IgG2a, κ
28	anti-mouse c-kit PE	eBioscience	12-1172-82	ACK2	Rat IgG2b, κ
29	anti-mouse Sca-1 PECy7	Ebiosciences	25-5981-82	D7	Rat IgG2a, κ
30	anti-mouse CD48 APC	Ebiosciences	17-0481-82	HM48-1	Armenian Hamster IgG
31	APC-Lineage antibody cocktail	BD Biosciences	558074	145-2C11	Armenian Hamster IgG1, κ
				M1/70	Rat IgG2b, κ
				RA3-6B2	Rat IgG2a, κ
				TER-119	Rat IgG2b, κ
				RB6-8C5	Rat IgG2b, κ
32	Rat IgG2a, κ FITC	BD Biosciences	553929	R35-95	
33	Rat IgG2a, κ PerCPCy5.5	BD Biosciences	550765	R35-95	
34	Rat IgG2a, κ APCCy7	BD Biosciences	552770	R35-95	
35	Rat IgG2a, λ PECy7	BD Biosciences	560721	B39-4	
36	Rat IgG2b, κ FITC	BD Biosciences	553988	A95-1	
37	Rat IgG2b, κ APC	BD Biosciences	553991	A95-1	
38	Rat IgG2b, κ PE	BD Biosciences	553989	A95-1	

39	Rat IgG2b, κ APCCy7	BD Biosciences	552773	A95-1	
40	Mouse IgG2a, κ APC	BD Biosciences	551414	G155-178	
41	Mouse IgG2a, κ PerCPCy5.5	BD Biosciences	552577	G155-178	
42	Mouse IgG2a, κ PECy7	BD Biosciences	552868	G155-178	
43	Mouse IgG2a, κ PE	BD Biosciences	553458	G155-178	
44	Armenian Hamster IgG APCCy7	Biolegend	400927	HTK888	
45	Armenian Hamster IgG FITC	Biolegend	400905	HTK888	
46	Armenian Hamster IgG PE	Biolegend	400907	HTK888	
47	Armenian Hamster IgG APC	Biolegend	400911	HTK888	
48	Donkey Anti-Goat IgG AF647	Jackson ImmunoResearch	705-607-003	Polyclonal	
49	Donkey Anti-Goat IgG AF488	Jackson ImmunoResearch	705-547-003	Polyclonal	
50	Goat Anti-Rabbit IgG AF 647	Jackson ImmunoResearch	111-607-003	Polyclonal	
51	Goat Anti-Rat IgG AF 647	Life Technologies, Invitrogen	A21247	Polyclonal	

Supplementary Videos

Video S1. Z-stack series and maximum projection of confocal images showing Postn expression in endoglin expressing vascular endothelial cells. Postn in red, endoglin in green and Hoechst 33342 in blue. Scale bar 10µm. Related to figure 6

Video S2. Z-stack series and maximum projection of zoomed-in confocal images showing Postn expression in endoglin expressing vascular endothelial cells. Postn in red, endoglin in green and Hoechst 33342 in blue. Scale bar 10µm. Related to figure 6

Video S3. Z-stack series and maximum projection of confocal images showing Postn expression in CD31 expressing vascular endothelial cells. Postn in red, CD31 in green and Hoechst 33342 in blue. Scale bar 10µm. Related to figure 6

Video S4. Z-stack series and maximum projection of zoomed-in confocal images showing Postn expression in CD31 expressing vascular endothelial cells. Postn in red, CD31 in green and Hoechst 33342 in blue. Scale bar 10µm. Related to figure 6

Video S5. Z-stack series and maximum projection of confocal images showing Postn co-localization with ECM protein laminin around CD31 expressing vascular endothelial cells. Postn in red, CD31 in green, laminin in blue and Hoechst 33342 in white. Scale bar 10µm. Related to figure 6

Video S6. Z-stack series and maximum projection of zoomed-in confocal images showing Postn co-localization with ECM protein laminin around CD31 expressing vascular endothelial cells. Postn in red, CD31 in green, laminin in blue and Hoechst 33342 in white. Scale bar 10µm. Related to figure 6

Supplemental experimental procedures

Animals: Six to eight weeks old FVB/NJ, C57BL/6J-CD45.2 (Centre d'Elevage R. Janvier, Le Genest-St Isle, *France)*, B6.SJL-PTPRCA-CD45.1 (Charles River Laboratories, Raleigh, NC), *Postn*$^{-/-}$ (generated in FVB/NJ background) [1], *Vav-iCre* [2] (from Thomas Graf, Centre for Genomic Regulation, Barcelona), *Itgav*$^{fl/fl}$ [3], *Rag2*$^{-/-}$*γC*$^{-/-}$ (from Prof. Chantal Mathieu, Clinical and Experimental Endocrinology, UZ Leuven, Leuven, Belgium) mice were used. The animals were bred at the animal facilities of KU Leuven, Rajiv Gandhi Centre for Biotechnology (RGCB) and IISER Thiruvananthapuram. During the experiments, mice were maintained in isolator cages, fed with autoclaved acidified water, and irradiated food ad libitum. At IISER TVM and RGCB, the animals were maintained as per guidelines provided by the Committee for the Purpose of Control and Supervision of Experiments on Animals (CPCSEA), Ministry of Environment and Forests, Government of India. All animal experiments were approved by the Institutional Animal Ethics Committees for the respective animal facilities.

The fetuses were isolated at E14.5 and genomic DNA from limb bud tissue was used for genotyping. Age of the fetuses was determined by considering the vaginal plug detection at E0.5. Primers used for genotyping of the *Vav-iCre; Itgav*$^{fl/fl}$ and *Postn*$^{-/-}$ lines are listed in Tabe S1.

Isolation of LT-HSC: LT-HSCs were sorted using specific antibodies to identify lin^{-}c-kit^{+}Sca-1^{+}CD150^{+}CD48^{-} cells. Fetal liver (FL) derived HSCs have been shown to express CD11b [4], therefore we removed anti-CD11b antibody from the lineage cocktail for sorting HSCs from FL tissues. The tissues were obtained from timed pregnant C57BL/6J female mice. Embryos were dissected at embryonic day (E) 14.5 (14 days after vaginal plug was observed). Mononuclear cell suspension was prepared by disrupting the tissue and repeated pipetting. The cells were washed with phosphate-buffered saline (PBS; Gibco Invitrogen, CA) containing 0.1% bovine serum albumin (BSA; Sigma). Ter-119 depletion was performed to deplete erythrocytes and erythroid progenitors by magnetic separation using MACS columns (Miltenyi Biotec, Germany). Ter-119 depleted cells were then stained with Alexa Fluor 488 conjugated Anti-lineage antibody cocktail (containing CD4, CD5, CD8a, CD45R, Ter-119, GR-1), PE conjugated anti-c-kit, APC conjugated anti-Sca-1, PE-Cy7 conjugated anti-CD150 and Alexa Fluor 488 conjugated anti-CD48. Cells were incubated on ice for 30 minutes. After staining the FL cells, HSCs were sorted by fluorescence-activated cell sorting (FACS), using a FACS ARIAIII (Becton Dickinson). Total adult bone marrow (ABM) cells, to be transplanted together with sorted HSCs for competitive repopulation assays, were flushed from femurs and tibiae of female C57BL/6J mice, washed twice with phosphate-buffered saline (PBS; Gibco Invitrogen, CA) containing 0.1% bovine serum albumin (BSA; Sigma). List of all antibodies used for flow cytometry based sorting and analysis is given in Table S2.

Cell Cycle analysis: Cell cycle analysis was performed by Hoechst 33342 (Ho) and Pyronin Y (PY) staining on cells labeled for HSPC markers as described before [5]. Fetal liver derived cells were first stained for HSPC markers (Lineage, Sca-1, c-kit) followed by Hoechst 33342 (Ho) alone or in combination with Pyronin Y (PY) staining as described before [5]. Cells were acquired on FACS Aria III (BD Biosciences) and analysed using FlowJo software (TreeStar, Ashland, OR).

Quantitative RT-PCR: Total RNA was prepared using the RNA Isolation Kit (Qiagen, Hilden, Germany), according to the manufacturer's protocol. DNase treatment of RNA was performed using Turbo DNase kit (Ambion, Austin, TX, USA). The purity and the concentration of RNA were assessed using a micro-volume spectrophotometer (Colibri, Berthold Technologies GmBH & Co. KG Germany). 100ng-1µg of RNA from each sample was used to synthesize cDNA using Superscript III First-Strand Synthesis System (Invitrogen, Carlsbad, CA) according to the manufacturer's protocol. qRT-PCR was carried out using Taqman SYBR green universal mix PCR reaction buffer (Applied Biosystems, Foster City, CA). The PCR reactions were carried out in a CFX96 detection system (Thermal-Cycler C1000, Biorad, Hercules, CA, USA). The list of primers used is given in Table S1.

Long-term repopulation assays: A single dose of 3.5 Gy (sub-lethal dose) or 10Gy (sub-lethal dose) radiation on a RAD SOURCE RS-2000 biological Irradiator (Rad Source Technologies, Alpharetta , GA) was given to the animals, a day before the test cells were transplanted. To compare the frequency of HSCs in E14.5 *Vav-Itgav*$^{+/+}$*, Vav-Itgav*$^{+/-}$ *and Vav-Itgav*$^{-/-}$ FL tissue, 10,000 FL mononuclear cells (CD45.2) along with 90,000 whole BM derived cells (CD45.1) were transplanted into lethally irradiated CD45.1 mice. For testing the function of HSC compartment, freshly sorted 100 HSCs from E14.5 *Vav-Itgav*$^{+/+}$*, Vav-Itgav*$^{+/-}$ *and Vav-Itgav*$^{-/-}$ FL tissues, along with 100,000 whole BM derived cells (CD45.1) were transplanted into lethally irradiated CD45.1 mice. For assessing the effect of Postn deletion on HSC frequency, 50,000 FL mononuclear cells derived from *Postn*$^{+/+}$ and *Postn*$^{-/-}$ embryos (CD45.1 FVB background) were transplanted into sub-lethally (3.5Gy) irradiated *Rag2*$^{-/-}$*γC*$^{-/-}$ mice (CD45.2 background). For testing the function of HSC compartment, freshly sorted 100 HSCs from *Postn*$^{+/+}$ and *Postn*$^{-/-}$ E14.5 FL tissues were transplanted into sub-lethally (3.5Gy) irradiated *Rag2*$^{-/-}$*γC*$^{-/-}$ mice. Peripheral blood chimerism analysis was performed every 4 weeks. After 12 weeks, half of the primary recipients were sacrificed; BM harvested, and 100,000 (for *Vav-Itgav*$^{-/-}$) or $1x10^6$ (for *Postn*$^{-/-}$) cells grafted in lethally or sub-lethally irradiated secondary recipients, respectively. After 3 months, chimerism in secondary recipients was evaluated. The second half of the primary recipients was followed for PB and BM chimerism for upto 24 weeks of transplantation

For chimerism analysis, the percentage of donor derived ($CD45.1^+$ or $CD45.2^+$) cells within the total leukocyte population ($CD45.1^+$ + $CD45.2^+$) was calculated. Multilineage-engraftment data was presented as the percentage of cells from myeloid, B- and T-cell lineage within the donor-derived population. For LSK chimerism in the BM, the proportion of donor-derived cells within the total LSK cell population was calculated and compared between the WT and KO groups.

Bioinformatics analysis: RNASeq analysis of the HSCs across developmental stages was previously published by us before [6]. The differential expression analysis of genes was performed using the R package DESeq2 [7] and differentially expressed genes were identified by the following thresholds: false discovery rate (FDR) < 0.05 and |log fold change| > 1.0. The gene-set enrichment test was then carried out to detect significantly enriched gene sets (also called pathways) using the R package GAGE v2.14.4 [8]. KEGG metabolic pathway maps were constructed to provide an overview of gene expression profiling and molecular interactions within a single pathway by using the R package Pathview [9]. The data can be accessed through ArrayExpress database (www.ebi.ac.uk/arrayexpress) under accession number E-MTAB-4034. This data was analyzed to examine the differential expression of all known genes for various α- and β-integrin chains and genes for pathways involved in DNA damage repair, namely mismatch repair, homologous recombination, nucleotide excision repair, and base excision repair. The lists of integrins and DNA repair pathway genes were extracted from MGI database and respective genes expression from RNA-seq was represented as heat-maps that were generated using Morpheus, https://software.broadinstitute.org/morpheus.

In vitro adhesion assays: For cell adhesion assays, the cells were first labeled with PKH26 membrane dye [10] according to the manufacturer's instructions (Sigma, St Louis, MO, USA). Freshly sorted or cultured LSK cells were harvested and washed with PBS to remove any protein content. The cells were re-suspended at $1x10^7$ per ml of Diluent C. The cell suspension was mixed with an equal volume of 4 mM PKH26 dye (in Diluent C) for 5min at room temperature. An equal volume of fetal bovine serum (FBS) was added to stop the reaction and the cells were washed with medium containing 10% FBS. $5x10^4$ ST2 cells were plated per well in 24 well plates. Ten thousand PKH-26 labeled LSK were added per well and incubated for 3h at 37°C and 5% CO_2. Non-adhered cells were removed and adhered cells were harvested along with the feeder layer. Flowcytometry was used to quantify the labeled cells to compare cell attachment [11]. Results are represented as percentage of cells adhered.

In vivo homing assay: A protocol published earlier [12] originally adapted from previously published reports [13,14] was used to examine the homing potential of BM derived HSPCs. One day prior to transplantation, 6-8 week old B6.SJL-PTPRCA-CD45.1 mice underwent total body lethal

irradiation (10Gy). Two million E14.5 FL cells from *Vav-Itgav*$^{+/+}$, *Vav-Itgav*$^{+/-}$ or *Vav-Itgav*$^{-/-}$ mice were injected intravenously into the irradiated mice. Prior to transplantation, the frequency of CFCs in the transplanted cells were quantified. 16 hours after injection, BM from transplanted mice was harvested and donor derived HSPCs homed in the recipient BM were quantified by CFU-C assay. For each experiment, a set of 6 mice irradiated but not transplanted was used to quantify left over CFCs in the BM of the irradiated mice. For CFC assays, $1x10^5$ cells were plated in 3 ml methylcellulose using MethoCult GF M3434 medium (Stem Cell Technologies). Each sample was cultured in triplicates at 37°C with 5% CO_2 in air. CFCs were scored after 9 to 12 days by light microscope. The proportion of total CFCs transplanted that homed in the total recipient BM represents homing.

Neutral comet assay: The DNA double strand break repair was assayed by neutral single-cell agarose gel electrophoresis, using a method earlier described by Olive et al. [15] and refined by Wojewódzka et al. [16]. The method was used to perform comet assay to assess DNA damage in freshly sorted and cultured hematopoietic stem cells from E14.5 FL and adult BM. Briefly, the test cells (freshly sorted or cultured) were mixed with low-gelling-temperature agarose (Sigma; type VII), and layered onto agarose-coated glass slides. Slides were maintained in the dark at 4°C to gel and for all the steps thereafter. Slides were submerged in lysis buffer (2.5 M NaCl, 0.1 M EDTA, 10 mM Trizma base, 1% Triton X-100, 10% DMSO) for 1.5 h, washed with Tris buffer, and incubated for 45 min in alkaline electrophoresis buffer (300 mM NaOH, 1 mM EDTA at pH 10) or in neutral electrophoresis buffer (300 mM sodium acetate, 100 mM Tris-HCl, 1% DMSO at pH 8.3). After electrophoresis (~40 min, 25 V), air-dried and neutralised slides were stained with 30 µl of ethidium bromide solution (20 µg/ml). Average Comet Tail Moment was scored for 100 cells/slide by using Comet Imager 1.2.10 software (MetaSystems Incorporation, Altlussheim, Germany).

Immunocytochemistry: Teflon-printed 10-well glass slides (Matsunami Glass Industry, Osaka, Japan) were pre-coated with Corning Cell-Tak™ adhesive and incubated for 2 hrs in a humidity chamber. Cell-Tak was removed and 500 of the BM derived LSK cells in 50µl PBS were added. The cells were incubated for 30 minutes at room temperature to adhere to the surface. The supernatant was carefully removed and 4% PFA was added for fixation at room temperature for 10 minutes. PFA was carefully removed and the cells were washed twice with PBS. AF647 conjugated anti-γH2AX antibody (1:100; clone N1-431; BD Pharmingen) in blocking buffer (3% BSA, 3% Goat Serum and 0.3% Triton X-100 in PBS) was added and kept in humidity chamber for overnight incubation at 4°C. The cells were washed twice using PBS and nuclei was counterstained with Hoechst 33342 (10µg/ml) for 2 mins and washed with PBS. Prolong Gold

antifade reagent (Invitrogen) was to mount the cells. The samples were air dried for 12 hrs in dark and visualised under Leica SP5 Confocal Upright Microscope (Leica Microsystems, Heidelberg, GmBH). Scoring of total γH2AX^{+} foci and the number of foci per nucleus was performed using Leica Application Suite X software.

Immunohistochemistry: E14.5 fetuses were harvested from pregnant dams, washed in PBS and fixed with 4% paraformaldehyde (PFA), on ice, for 4 hours. Paraffin blocks were made of the fixed fetuses by following gradient steps for dehydration, clearing and infiltration and finally embedding in fresh Paraplast (Sigma). Microtome (Leica RM2265) was used to cut 10μm longitudinal sections of E14.5 embryo for immunohistochemical assays on FFPE sections. Alternatively, FL tissues were dissected out from the fetuses and subjected to a 30 minute fixation with 2% PFA, on ice. Fixed tissues were subjected to overnight 30% sucrose gradient for cryo-protection prior to cryo-block preparation using Polyfreeze (Sigma). Cryotome (Thermo Scientific HM525 NX) was used to obtain 50μm thick sections of E14.5 FL for immunohistochemical assay on cryo-sections.

FFPE sections on frosted slides (VWR) or floating cryo-sections were immune-labeled using antigen specific primary antibodies; anti-Postn antibody (R&D systems, Minneapolis, MN), AF647 conjugated anti-mouse CD31 antibody (BioLegend), biotin conjugated anti-mouse Endoglin antibody (Invitrogen) and fluorescently labeled secondary antibodies (Jackson ImmunoResearch Laboratories Inc.). Hoechst 33342 (Sigma Aldrich) was used to counterstain the nuclei. Sections were mounted using Prolong Gold antifade mounting medium (Invitrogen). Fluorescence imaging was performed using Leica upright confocal microscope (Leica TCS SP5 II). Images were captured using 63X oil immersion objective (HCX PL APO CS 63.0x1.40 Oil). For imaging whole embryo section, tile scanning followed by auto-stitching was performed using LasAF software. Z-stacks were taken with 0.35μm step size and LasX software was used for analyzing the obtained images.

Statistical analysis: All data are represented as mean ± s.e.m. Normal distribution of data was tested using the Shapiro-Wilk test. The equality of group variance was tested using Brown-Forsythe test. Comparisons between samples from two groups with normally distributed data with equal variance were made using the unpaired two-tailed Student's *t-test*. Mann Whitney test was used for comparing two groups where data were non-normally distributed. For multiple comparisons of the normally distributed data with equal variance, one-way ANOVA was performed followed by Tukey Kramer post hoc test. Non-normally distributed data was analyzed by Friedman test. Chi-square test was employed for testing the goodness of fit of the observed ratios among genotypes of embryos to expected Mendelian ratio. Statistical analyses were

performed with Microsoft Excel or GraphPad Prism 6. For all analyses, p-values ≤0.05 were accepted as statistically significant.

Supplemental references

1. Malanchi I, Santamaria-Martinez A, Susanto E, et al. Interactions between cancer stem cells and their niche govern metastatic colonization. *Nature*. 2012;481(7379):85-89.

2. de Boer J, Williams A, Skavdis G, et al. Transgenic mice with hematopoietic and lymphoid specific expression of Cre. *Eur J Immunol*. 2003;33(2):314-325.

3. Lacy-Hulbert A, Smith AM, Tissire H, et al. Ulcerative colitis and autoimmunity induced by loss of myeloid alphav integrins. *Proc Natl Acad Sci U S A*. 2007;104(40):15823-15828.

4. Morrison SJ, Hemmati HD, Wandycz AM, Weissman IL. The purification and characterization of fetal liver hematopoietic stem cells. *Proc Natl Acad Sci U S A*. 1995;92(22):10302-10306.

5. Arai F, Hirao A, Ohmura M, et al. Tie2/angiopoietin-1 signaling regulates hematopoietic stem cell quiescence in the bone marrow niche. *Cell*. 2004;118(2):149-161.

6. Manesia JK, Xu ZF, Broekaert D, et al. Highly proliferative primitive fetal liver hematopoietic stem cells are fueled by oxidative metabolic pathways. *Stem Cell Research*. 2015;15(3):715-721.

7. Love MI, Huber W, Anders S. Moderated estimation of fold change and dispersion for RNA-seq data with DESeq2. *Genome Biol*. 2014;15(12):550.

8. Luo W, Friedman MS, Shedden K, Hankenson KD, Woolf PJ. GAGE: generally applicable gene set enrichment for pathway analysis. *BMC Bioinformatics*. 2009;10:161.

9. Luo W, Brouwer C. Pathview: an R/Bioconductor package for pathway-based data integration and visualization. *Bioinformatics*. 2013;29(14):1830-1831.

10. Hendrikx PJ, Martens CM, Hagenbeek A, Keij JF, Visser JW. Homing of fluorescently labeled murine hematopoietic stem cells. *Experimental hematology*. 1996;24(2):129-140.

11. Simmons PJ, Masinovsky B, Longenecker BM, Berenson R, Torok-Storb B, Gallatin WM. Vascular cell adhesion molecule-1 expressed by bone marrow stromal cells mediates the binding of hematopoietic progenitor cells. *Blood*. 1992;80(2):388-395.

12. Khurana S, Buckley S, Schouteden S, et al. A novel role of BMP4 in adult hematopoietic stem and progenitor cell homing via Smad independent regulation of integrin-alpha4 expression. *Blood*. 2013;121(5):781-790.

13. Szilvassy SJ, Meyerrose TE, Ragland PL, Grimes B. Differential homing and engraftment properties of hematopoietic progenitor cells from murine bone marrow, mobilized peripheral blood, and fetal liver. *Blood*. 2001;98(7):2108-2115.

14. Frenette PS, Subbarao S, Mazo IB, von Andrian UH, Wagner DD. Endothelial selectins and vascular cell adhesion molecule-1 promote hematopoietic progenitor homing to bone marrow.

Proceedings of the National Academy of Sciences of the United States of America. 1998;95(24):14423-14428.

15. Olive PL, Banath JP, Durand RE. Heterogeneity in Radiation-Induced DNA Damage and Repair in Tumor and Normal-Cells Measured Using the Comet Assay. *Radiation Research*. 1990;122(1):86-94.

16. Wojewodzka M, Buraczewska I, Kruszewski M. A modified neutral comet assay: elimination of lysis at high temperature and validation of the assay with anti-single-stranded DNA antibody. *Mutation Research-Genetic Toxicology and Environmental Mutagenesis*. 2002;518(1):9-20.
